# Supplementary material for: Building a Boot Camp: Pediatric Residency Preparatory Course Design Workshop and Tool Kit
Source: MedEdPORTAL. 2019 Dec 13;15:10860. doi: 10.15766/mep_2374-8265.10860 (PMC7010200; doi:10.15766/mep_2374-8265.10860)
Supplement: Supplementary file 1 — A. Boot Camp Workshop Presentation.pptx B. Review of Existing Boot Camp Literature.docx C. Institutional Needs Assessment Worksheet.docx D. Recommended Content List and Session Prioritization Worksheet.docx E. Schedule Worksheet and Sample Schedules.docx F. Module Design Worksheet and Planning Resources.docx G. Selected MedEdPORTAL Boot Camp Resources.docx H. Workshop Feedback Surveys.docx I. Facilitator Guide.docx [file mep-15-10860-s001.zip › _Educational_Summary_Report_10860.pdf]

MedEdPORTAL® | The AAMC Journal of Teaching and Learning Resources

Original Publication

Open Access

Building a Boot Camp: Pediatric Residency Preparatory Course Design Workshop and Tool Kit

Amanda Hartke, MD, PhD*, Erin Pete Devon, MD, Rebekah Burns, MD, Molly Rideout, MD

*Corresponding author: Amanda.Hartke@prismahealth.org

Abstract

Introduction: Although many medical schools are adding residency preparatory courses or boot camps to their curricula, there is little published guidance for faculty tasked with designing them. We developed a workshop and accompanying boot camp course design tool kit to assist faculty in creating a pediatric boot camp course following the initial steps of Kern's framework for curriculum development. **Methods:** Learners participated in a 2-hour workshop incorporating short didactics, guided independent reflection, and group discussions. Workshop facilitators guided faculty through the tool kit materials including a literature overview, a needs assessment worksheet, session prioritization and schedule planning worksheets, a module design worksheet, and implementation strategies. **Results:** Twenty-seven attendees at a national meeting of undergraduate pediatric educators participated in the workshop. Feedback was solicited via an anonymous electronic survey (41% completion rate), which indicated that attendees' self-assessed confidence significantly increased for each component of the tool kit. For the five tool kit components surveyed, average confidence increased 26% (range: 17.5%-37.1%) after completing the workshop. All respondents also indicated that the tool kit would be moderately helpful to very helpful as a stand-alone resource for independent faculty use, corresponding to a 3.57 out of 5 weighted average for this Likert-scale question. **Discussion:** We developed a pediatric boot camp course design workshop and tool kit to assist faculty in developing pediatric boot camps. Initial implementation was through a workshop, but the resource could be used individually and also adapted for use by other specialties.

Keywords
Boot Camp, Residency Preparatory Course, Capstone Course, Pediatrics, Curriculum Development, Tool Kit, Faculty Development, Editor's Choice

Educational Objectives

By the end of this activity, learners will be able to:

1. Discuss the literature pertaining to residency preparatory courses or boot camps.
2. Perform a boot camp needs assessment related to available resources, identified gaps, and institutional requirements.
3. Design boot camp course schedules aligned with individualized needs assessments.
4. Develop module ideas to use in boot camp courses that address core entrustable professional activities.
5. Identify barriers and strategies to successfully implement boot camp courses at home institutions.

Citation:
Hartke A, Pete Devon E, Burns R, Rideout M. Building a boot camp: pediatric residency preparatory course design workshop and tool kit. *MedEdPORTAL*. 2019;15:10860.
https://doi.org/10.15766/mep_2374-8265.10860

Introduction

Arising from an identified gap between the starting expectations for interns and the variability of fourth-year medical school curricula, boot camps (also referred to as residency preparatory courses) have gained interest in medical education as a way to further prepare students for the transition from medical school to internship.[1-3] A meta-analysis on the effectiveness of boot camps for transitions into residency concluded that completion of such a course was associated with improved clinical skills, knowledge acquisition, and perceived confidence.[1] Designing a boot camp course is complex, as there is wide variability in content, duration, and timing of courses, as well as institution-specific requirements.[4] Faculty are frequently asked to develop courses without any guidance, and clinical faculty who are developing the courses often have limited formal training in medical education.

Copyright © 2019 Hartke et al. This is an open-access publication distributed under the terms of the Creative Commons Attribution license.

The American College of Surgeons, the Association of Program Directors in Surgery, and the Association for Surgical Education have jointly developed a modular resident preparatory curriculum recommended for students prior to the beginning of surgical internship.[5] Otherwise, there are few published discipline-specific boot camp content recommendations or faculty development resources.[6,7] Although there is a recent publication on pediatric boot camp program development and evaluation, based on a conceptual framework,[8] there is no published literature for specifically designing pediatric boot camp curricula. Numerous published modular sessions could easily be incorporated into boot camps,[9-12] and there are multiple publications describing institutional experiences with boot camp implementation.[1,13]

This workshop introduced faculty to the pediatric boot camp course design tool kit, which serves as a framework for faculty tasked with boot camp course development. It was designed to guide participants through the initial stages of Kern's six steps of curriculum development[14] and to assist faculty in designing a course that addresses entrustable professional activities (EPAs) for entering residency through development of new sessions or adaptation of published modules. Participants were encouraged to prioritize course content based on the duration of boot camp, physical resources, and local expertise to design a course meeting individual institutional needs. The materials provided in the appendices include the slide presentation used in the workshop, both blank and completed sample worksheets for each section of the tool kit, and a facilitator guide. There is also a review of the existing boot camp literature for additional preparation for facilitators. The target audience of the workshop and associated tool kit is faculty tasked with designing and implementing boot camp courses that are competency driven and provide maximal preparation for students entering a pediatric or pediatric-involved residency.

Methods

Curricular Context

We developed this workshop and tool kit to guide physicians and educators involved in undergraduate medical education through the steps of designing a pediatric boot camp for fourth-year medical students. We introduced the tool kit as part of a 2-hour workshop during the Council on Medical Student Education in Pediatrics (COMSEP) Annual Meeting in April 2018. Participants primarily included pediatricians involved in undergraduate medical education with an interest in boot camps. We used Kern's six steps of curriculum development as a theoretical model for content development.[14] These steps include the following:

1. Problem identification and general needs assessment.
2. Targeted needs assessment.
3. Goals and objectives.
4. Educational strategies.
5. Implementation.
6. Evaluation and feedback.

Of note, program evaluation was not specifically addressed within the workshop or tool kit because individual institutions often have local practices for gathering course feedback. Prerequisite knowledge about objective writing,[15] basic curricular design,[16] the core EPAs,[17] and pediatric milestones[18] was beneficial but not required. This tool kit could be tailored for use by faculty in other specialties as well.

Implementation

The workshop included a mixture of short didactics, guided reflective exercises, and both small- and large-group discussions. Four facilitators with experience in boot camp development created the tool kit and facilitated the workshop. We organized tables and chairs in the conference room to allow for optimal small-group discussion. Materials included a computer and projector, a PowerPoint presentation (Appendix A), pens, and the tool kit materials (Appendices B-H). We used the PowerPoint presentation (Appendix A) throughout the workshop to emphasize teaching points, transition between exercises, and guide activities. Timing and sequence are detailed in Table 1. We also created a facilitator guide (Appendix I), which reviews the tool kit materials provided in the appendices and includes recommendations and practical instructions for planning the workshop, facilitating each of the workshop sections using these materials, adapting the workshop for participants either planning de novo boot camps or revising an existing course, and conducting the follow-up survey.

Initially, we reviewed existing background literature pertaining to the current state of the fourth year of medical school,[19-22] with particular emphasis on boot camps, both general and pediatric specific. We then guided participants in performing a needs assessment or adapting one they may have previously performed. Next, they created sample schedules tailored to the identified needs of their home institution. Participants then designed a sample module targeting specific EPAs. Finally, we discussed barriers to boot camp implementation and possible solutions. The workshop was intended to provide participants with knowledge and resources to develop pediatric boot camp–style courses de novo or improve on existing courses.

Copyright © 2019 Hartke et al. This is an open-access publication distributed under the terms of the Creative Commons Attribution license.

Table 1. Schedule of Pediatric Boot Camp Workshop During the 2018 Council on Medical Student Education in Pediatrics Conference

Duration	Activity	Format	Slides (Appendix A)	Additional Resources
15 minutes	Introduction/background	Presentation	1-14	Appendix B
5 minutes		Presentation	15-19	
10 minutes	Needs assessment	Individual reflection	20	Appendix C
10 minutes		Small group	20	
5 minutes		Presentation	21-22	
15 minutes	Sample schedule	Small group	22	Appendices D & E
10 minutes		Large group	23	
5 minutes		Presentation	24-26	Appendices F & G
20 minutes	Module development	Small group	27	Appendices F & G
10 minutes		Large group	28	Appendices F & G
5 minutes	Barriers	Large group	29-30	
5 minutes	Summary	Presentation	31	

Introduction, audience identification, and review of objectives: Initially, each of the facilitators gave a brief introduction, sharing personal experience with boot camp courses and curriculum development. We then asked participants to share their own experiences with the development and implementation of boot camps. This allowed us to gauge our audience's level of background knowledge and experience, as well as leverage participants' experiences for future discussion. Finally, one facilitator reviewed the objectives for the workshop.

Review of existing background literature: We provided background on the variability of the fourth year of medical school; perceived gaps in interns' knowledge, skills, and attitudes; and the use of boot camps for graduating medical students across different specialties, with emphasis on pediatric-specific courses. This allowed participants to understand the context of boot camps in medical education and the variability of the courses across institutions. A summary and references for this review are provided in Appendix B.

Needs assessment: The goal of this exercise was to guide the participants in planning a boot camp–specific needs assessment, taking into account institutional, trainee, and residency program director perspectives. First, we instructed participants to reflect on any boot camp needs assessment conducted previously, including the specific methods used and stakeholders involved. Using the institutional needs assessment worksheet (Appendix C), participants then created a plan for performing an assessment or improving on an existing assessment at their own institution, including strategies for soliciting input from stakeholders. Participants identified some of the basic considerations for their courses, including the number of instructional days, students needing to enroll, and faculty required. They also listed basic resources available to them, including physical space, simulation centers, standardized patients, among others. Finally, participants completed a matrix relating to core EPAs currently addressed in the curriculum to identify potential gaps that could be addressed in a boot camp. Following brief individual reflection, participants discussed ideas in small groups of three to six people. Facilitators visited tables throughout the exercise to provide additional direction as needed.

Schedule development: After a brief introduction, we asked participants to individually brainstorm general session topics identified during the needs assessment portion. Using the recommended content list and session prioritization worksheet (Appendix D), they prioritized sessions based on core EPA topics and identified educational gaps, as well as the resources and time allotment available, at their institutions. The recommended content areas represented a consensus list from our own experience considering the knowledge, skill, and behavioral gaps identified in the literature review included in the background (Appendix B). Since creation of the workshop, this content area has been reviewed, and a curriculum has been vetted through COMSEP that is now published on the COMSEP website.[23] After completing their individual prioritization lists, participants formed new groups of three to six members based on boot camp course length (3 days or less, 5 days, 10 days, more than 10 days). Each group created a draft schedule using the schedule worksheet with sample schedules from the facilitators' institutions as examples (Appendix E). In a large-group format, facilitators led a focused debriefing session about the experience of developing a course schedule.

Module development: We began this activity with a brief discussion about how to identify instructional methods to address learning objectives within practical constraints such as time and physical resources. We then assigned each small group up to three EPAs. Using the module design worksheet and planning resources (Appendix F), participants considered which educational method could best be applied to their assigned EPAs. Each group outlined a module to address one of its assigned

Copyright © 2019 Hartke et al. This is an open-access publication distributed under the terms of the Creative Commons Attribution license.

EPAs by creating learning objectives, identifying the number of learners that could be accommodated per session, describing the faculty/resource needs and time required, and identifying an instructional method. Additional resources used for this exercise were a list of relevant resources available in *MedEdPORTAL* (Appendix G). Each small group then presented its module to the large group and received feedback from participants and facilitators.

Barriers: During a large-group discussion, we asked participants to identify barriers that they had encountered or anticipated encountering while designing and implementing boot camps. Common themes surfaced, including limitations with physical and monetary resources, coordinator or administrative support, faculty volunteers, institutional support, student participation, and objective assessment of students. We facilitated a brief discussion about potential solutions.

Wrap-up: We closed the workshop with a summary of the key points and encouraged participants to seek out existing resources and to collaborate across institutions.

Assessment

Immediately following the workshop, we emailed an anonymous electronic survey (Appendix H) via SurveyMonkey (San Mateo, California) to all participants asking about their personal and institutional experience with boot camp courses, as well as their self-assessed confidence in the components covered in the workshop, including the following:

1. Discussing the literature pertaining to boot camp courses;
2. Performing a needs assessment related to available resources, identified gaps, and institutional requirements;
3. Designing a boot camp schedule aligned with individualized needs assessments;
4. Developing modules to use in a boot camp that address core EPAs; and
5. Identifying barriers and strategies to successfully implement a boot camp at their home institutions.

The survey collected retrospective pre-/postworkshop self-assessed confidence responses using visual analog scales measuring from 0 to 100 for each of the five components. The survey was open for 1 month following the workshop, and we sent one reminder email 3 weeks after the initial request. We compared the mean level of self-assessed confidence pre- and postworkshop for the five components of course planning and design addressed in the boot camp course design tool kit. Participants' change in confidence level was then compared using a paired two-tailed Student *t* test. Four months following the workshop, we emailed a second anonymous electronic survey to all participants asking about the impact of the workshop on their institution's boot camp course (Appendix H).

Results

During the COMSEP 2018 Annual Meeting, 27 of the conference attendees self-selected for participation in the workshop. There were response rates of 41% (11 out of 27) to the initial postworkshop electronic survey and 27% to the follow-up survey 4 months later. Table 2 presents demographics of course participants responding to the initial survey.

There was a statistically significant increase in self-assessed confidence for all five components of course planning and design addressed in the workshop (Table 3). The lowest preconference confidence levels were in the areas of discussing the literature around boot camps and developing modules to address EPAs. These categories were also associated with the greatest average increase in confidence following participation in the workshop.

Following is a representative sample of qualitative responses from the initial postworkshop survey:

- Prompt: Name one thing you plan to implement from this workshop in the next year.
  - "Needs assessment" (three responses).
  - "Even if institutionalized curriculum does not get up and running, I plan on trying to offer an experience for interested 4th years that may be a 1-2 week experience depending on my availability."

Table 2. Boot Camp Experience and Boot Camp Structure Among Course Participants

Survey Item (*n*)	Item Answer	Percentage of Respondents
Institutional experience (11)	Existing boot camp	45
	Planning a boot camp	55
Structure (10)	Pediatric specific	30
	General with a pediatric component	60
	Undecided	10
Duration (10)	≤1 week	20
	>1 week and <4 weeks	40
	≥4 weeks	20
	Undecided	20
Enrollment (10)	Required	20
	Elective	50
	Undecided	30
Timing (10)	Prior to match	20
	Following match	50
	Undecided	30

Copyright © 2019 Hartke et al. This is an open-access publication distributed under the terms of the Creative Commons Attribution license.

Table 3. Retrospective Mean Confidence of the Five Covered Components Pre- and Postworkshop

Survey Item (*n*)	Preworkshop *M* (*SD*)	Postworkshop *M* (*SD*)	Pre-Post Difference *M* (95% CI)	*p*
Confidence discussing the literature pertaining to boot camp courses (10)	29 (23)	57 (30)	29 (11-46)	.005
Confidence performing a needs assessment related to available resources, identified gaps, and institutional requirements (11)	52 (35)	77 (27)	25 (10-40)	.005
Confidence designing a boot camp course schedule aligned with individualized needs assessments (11)	48 (31)	71 (15)	23 (6-40)	.01
Confidence developing module ideas for a boot camp course that address core entrustable professional activities (10)	32 (27)	69 (15)	37 (17-58)	.003
Confidence identifying barriers and strategies to successfully implement a boot camp course at your home institution (11)	56 (28)	73 (17)	18 (1-34)	.04

  - "More on interdisciplinary communication and 'giving bad news.'"
  - "Choosing more engaging activities for certain existing modules."
  - "Investigate online modules such as choosing wisely to use."
  - "A boot camp!"
- Prompt: Is there any other feedback you would like to offer the presenters?
  - "Great generalized discussion, appreciate sharing resources!"
  - "It was great to hear how others approach these. All are different and walked away with some possible additions to our curriculum."
  - "Wonderful job. The literature and schedules shared were so very valuable. Sharing the barriers and some novel solutions as well as innovative teaching methods was greatly appreciated."
  - "Well done. The resources you provided are exceptional."

On the follow-up electronic survey 4 months after the workshop, all respondents who did not have a boot camp before participating ($n = 3$) reported that completing the workshop assisted them in planning or implementing a new boot camp. All respondents ($n = 7$) ranked the materials and content from the workshop as being moderately helpful or very helpful as a self-contained resource for faculty to use independently when planning a boot camp based on a 5-point Likert scale (1 = *not helpful at all*, 2 = *slightly helpful*, 3 = *moderately helpful*, 4 = *very helpful*, 5 = *extremely helpful*), with a weighted average of the responses of 3.57 and a range of 3 to 4.

Responses from participants without a preexisting boot camp ($n = 3$) included the following:

- Prompt: Which of the workshop exercises and materials were helpful in planning or implementing a new boot camp?
  - Boot camp background literature review ($n = 1$).
  - Example schedules from facilitators' institutions ($n = 2$).
  - Sample schedule worksheet exercise ($n = 1$).
  - Example *MedEdPORTAL* boot camp module list ($n = 2$).
  - Module design worksheet exercise based on selected EPAs ($n = 1$).
  - Implementation strategies and barriers discussion ($n = 2$).
- Prompt: Please specify how your participation in the workshop helped you develop a boot camp.
  - "Bootcamp was already being planned by the medical school, but I was able to bring some useful ideas to the planning group due to workshop participation."
  - "Utilizing ideas from workshop discussions in creation of bootcamp at my institution."

Responses from participants with a preexisting boot camp ($n = 4$) included the following:

- Prompt: Which of the workshop exercises and materials were helpful in making changes to your boot camp?
  - Needs assessment worksheet exercise ($n = 1$).
  - Module design worksheet exercise ($n = 1$).
- Prompt: What specific changes did you make to your existing boot camp as a result of your participation in the workshop?
  - *"For the upcoming year will incorporate more on advocacy, poverty and wellness."*

Discussion

Preresidency boot camps for graduating medical students are rapidly growing in popularity and, in some cases, are required courses. This resource aims to provide guidance to faculty so that they can develop the courses expeditiously, paying attention to the particular needs and requirements of their institutions. Workshop participants used the pediatric boot camp course design tool kit to develop new courses and refine ongoing curricula at their home institutions, guided by the initial stages of Kern's six steps for curriculum development.[14] By working

Copyright © 2019 Hartke et al. This is an open-access publication distributed under the terms of the Creative Commons Attribution license.

through a needs assessment, developing targeted goals and objectives, designing modules, and considering barriers to implementation, participants successfully used the tool kit to begin the design and improvement of pediatric boot camps. Participants reported a statistically significant increase in confidence for all five components of the workshop following participation, and several reported using components of the tool kit to continue designing and improving boot camps following the workshop. All survey respondents reported that the tool kit would be at least moderately helpful as a self-contained resource for faculty to use in designing boot camp courses.

Our findings are important because there are a very few published resources to help faculty who are designing boot camp courses. Faculty could use the materials to facilitate a similar workshop, thereby assisting others in designing pediatric boot camp courses. Alternatively, considering that the pediatric boot camp course design tool kit is a self-contained resource, faculty could use it independently to design a single course. Last, although this workshop and the associated tool kit were designed with a focus on pediatric boot camps, the steps of curriculum development are the same for any discipline, and with minor adaptations, the materials could be used to design either generalized boot camps for an entire senior medical school class or specialty-specific boot camps for students pursuing a nonpediatric residency.

The pediatric boot camp course design workshop and tool kit add to the growing literature surrounding development of preresidency boot camps. The field of surgery has a robust national curriculum being pilot tested across dozens of institutions[5]; however, other disciplines have not developed supporting materials as quickly, despite similar institutional requirements to provide training in this area. This resource is an effort to continue to build and develop coordinated resources to maximize intern preparedness.

For faculty with existing boot camps, this workshop and tool kit provide structured materials that can guide users to reassess and revise their curricula. This is especially important following initial boot camp implementation or significant institutional curricular changes that could affect stakeholder needs and content goals. Included in the survey results presented above, workshop attendees with existing boot camps planned adjustments to their courses, including incorporation of new content and revision of module design to make activities more engaging. Given the rapidly evolving literature surrounding the transition from medical school to residency, revisiting an existing curriculum through the lens of this tool kit may help users continually improve on the relevancy and efficacy of their courses.

There are some limitations of this workshop and tool kit. First, considering that the workshop was presented nationally at a single venue, the number of participants was relatively small. Although we plan on further dissemination through future workshops and faculty development sessions, we felt that it was important to publicize the work so that faculty would be able to access it. Also, the response rate for the surveys was relatively low, likely reflecting survey fatigue. In the future, we plan to provide paper surveys at the end of the workshop (not sent over email following the workshop) and also hold focused interviews about the tool kit to discern the most useful aspects. In addition, we learned several specific lessons related to the tool kit that may be helpful for people using it. During the needs assessment, we found that peer sharing helped participants identify potential needs and topics they had not considered previously. For faculty using this tool kit outside of the workshop format, it may be useful to enlist others within the institution to provide this form of peer sharing. When creating sample schedules, participants ranked most choices in the highest, need-this category, making it difficult for them to prioritize content, especially in the shorter boot camps. An additional step of dividing content into general and pediatric specific could help participants with shorter boot camps to prioritize pediatric content. Our workshop did not specifically address whether boot camps should be an elective or required course, although the topic was discussed during the barriers and solutions segment of the presentation. This status varies within our own institutions and remains an area for additional exploration in the literature. Finally, the workshop could be led by a single facilitator; however, having multiple facilitators allowed for more interaction and guidance during the small-group exercises.

By implementing this workshop and disseminating the tool kit for widespread use, it is our hope that educators will be able to efficiently develop pediatric boot camps that address the specific requirements and needs of individual institutions while simultaneously uniformly preparing students for the crucial transition to intern physician. It is also our hope that through continued collaboration, educators will develop and share resources related to residency preparedness so as to build robust learning resources for use by all. Future work includes collaborating with both COMSEP and the Association of Pediatric Program Directors to establish standard resources for programs to use in pediatric residency preparatory courses.

Copyright © 2019 Hartke et al. This is an open-access publication distributed under the terms of the Creative Commons Attribution license.

Appendices

A. Boot Camp Workshop Presentation.pptx

B. Review of Existing Boot Camp Literature.docx

C. Institutional Needs Assessment Worksheet.docx

D. Recommended Content List and Session Prioritization Worksheet.docx

E. Schedule Worksheet and Sample Schedules.docx

F. Module Design Worksheet and Planning Resources.docx

G. Selected MedEdPORTAL Boot Camp Resources.docx

H. Workshop Feedback Surveys.docx

I. Facilitator Guide.docx

All appendices are peer reviewed as integral parts of the Original Publication.

Amanda Hartke, MD, PhD: Assistant Clinical Professor, Department of Pediatrics, Prisma Health Upstate/University of South Carolina School of Medicine Greenville

Erin Pete Devon, MD: Assistant Professor of Clinical Pediatrics, Department of Pediatrics, Children's Hospital of Philadelphia

Rebekah Burns, MD: Associate Professor, Department of Pediatrics, University of Washington School of Medicine

Molly Rideout, MD: Associate Professor, Department of Pediatric Hospital Medicine, Robert Larner, M.D. College of Medicine at the University of Vermont

Disclosures
None to report.

Funding/Support
None to report.

Prior Presentations
Rideout M, Burns R, Pete Devon E, Hartke A. Will they be ready? Designing effective and engaging pediatric residency preparatory courses (boot camps) for fourth year students. Workshop presented at: COMSEP Annual Meeting; April 13, 2018; Denver, CO.

Ethical Approval
Reported as not applicable.

References

1. Blackmore C, Austin J, Lopushinsky SR, Donnon T. Effects of postgraduate medical education "boot camps" on clinical skills, knowledge, and confidence: a meta-analysis. *J Grad Med Educ*. 2014;6(4):643-652. https://doi.org/10.4300/JGME-D-13-00373.1
2. Teo AR, Harleman E, O'Sullivan PS, Maa J. The key role of a transition course in preparing medical students for internship. *Acad Med*. 2011;86(7):860-865. https://doi.org/10.1097/ACM.0b013e31821d6ae2
3. Reddy ST, Chao J, Carter JL, et al. Alliance for Clinical Education perspective paper: recommendations for redesigning the "final year" of medical school. *Teach Learn Med*. 2014;26(4):420-427. https://doi.org/10.1080/10401334.2014.945027
4. Pete Devon E, Ronan J, Tenney-Soeiro R, Balmer D. Current state of the intern preparatory course: findings from a national survey of pediatric clerkship directors. *J Fam Med Community Health*. 2017;4(6):1124-1128.
5. ACS/APDS/ASE Resident Prep Curriculum. American College of Surgeons website. https://www.facs.org/education/program/resident-prep. Accessed September 26, 2019.
6. Lamba S, Wilson B, Natal B, Nagurka R, Anana M, Sule H. A suggested emergency medicine boot camp curriculum for medical students based on the mapping of core Entrustable Professional Activities to Emergency Medicine Level 1 milestones. *Adv Med Educ Pract*. 2016;7:115-124. https://doi.org/10.2147/AMEP.S97106
7. Lerner V, Higgins EE, Winkel A. Re-boot: simulation elective for medical students as preparation bootcamp for obstetrics and gynecology residency. *Cureus*. 2018:10(6):e2811. https://doi.org/10.7759/cureus.2811
8. Pete Devon E, Tenney-Soeiro R, Ronan J, Balmer DF. A pediatric preintern boot camp: program development and evaluation informed by a conceptual framework. *Acad Pediatr*. 2019;19(2):165-169. https://doi.org/10.1016/j.acap.2018.08.006
9. Burns R, Mangold K, Adler M, Trainor J. Pediatric Boot Camp Series: obtaining a consult, discussing difficult news. *MedEdPORTAL*. 2016;12:10437. https://doi.org/10.15766/mep_2374-8265.10437
10. Burns R, Nicholson A, Mangold K, Adler M, Trainor J. Pediatric Boot Camp Series: assessment and plans, task prioritization, answering pages, handoffs. *MedEdPORTAL*. 2015;11:10310. https://doi.org/10.15766/mep_2374-8265.10310
11. Metz J, Stone K, Reid J, Burns R. Pediatric Boot Camp Series: infant with altered mental status and seizure—a case of child abuse. *MedEdPORTAL*. 2017;13:10552. https://doi.org/10.15766/mep_2374-8265.10552
12. Smith S, Hayward K, Kronman M. Interactive pediatric cases for senior capstone courses. *MedEdPORTAL*. 2009;5:7743. https://doi.org/10.15766/mep_2374-8265.7743
13. Burns R, Adler M, Mangold K, Trainor J. A brief boot camp for 4th-year medical students entering into pediatric and family medicine residencies. *Cureus*. 2016;8(2):e488. https://doi.org/10.7759/cureus.488
14. Thomas PA, Kern DE, Hughes MT, Chen BY, eds. *Curriculum Development for Medical Education: A Six-Step Approach*. 3rd ed. Baltimore, MD: Johns Hopkins University Press; 2016.

Copyright © 2019 Hartke et al. This is an open-access publication distributed under the terms of the Creative Commons Attribution license.

15. Anderson LW, Krathwohl DR, eds. *A Taxonomy for Learning, Teaching, and Assessing: A Revision of Bloom's Taxonomy of Educational Objectives*. New York, NY: Longman; 2001.
16. Prideaux D. Curriculum design. *BMJ*. 2003;326:268. https://doi.org/10.1136/bmj.326.7383.268
17. *Core Entrustable Professional Activities for Entering Residency: Curriculum Developers' Guide*. Washington, DC: Association of American Medical Colleges; 2014.
18. The Pediatrics Milestone Project: a joint initiative of the Accreditation Council for Graduate Medical Education and the American Board of Pediatrics. Accreditation Council for Graduate Medical Education website. https://acgme.org/Portals/0/PDFs/Milestones/PediatricsMilestones.pdf. Published July 2017. Accessed July 6, 2018.
19. Lyss-Lerman P, Teherani A, Aagaard E, Loeser H, Cooke M, Harper GM. What training is needed in the fourth year of medical school? Views of residency program directors. *Acad Med*. 2009;84(7):823-829. https://doi.org/10.1097/ACM.0b013e3181a82426
20. Walling A, Merando A. The fourth year of medical education: a literature review. *Acad Med*. 2010;85(11):1698-1704. https://doi.org/10.1097/ACM.0b013e3181f52dc6
21. Goldfarb S, Morrison G. The 3-year medical school—change or shortchange? *N Engl J Med*. 2013;369(12):1087-1089. https://doi.org/10.1056/NEJMp1306457
22. Kanter SL. How to win an argument about the senior year of medical school. *Acad Med*. 2009;84(7):815-816. https://doi.org/10.1097/ACM.0b013e3181ae1f13
23. Rideout M, Burns R, Pete Devon E, Hartke A. COMSEP pediatric residency preparatory curriculum. Council on Medical Student Education in Pediatrics (COMSEP) website. https://www.comsep.org/pediatric-residency-preparatory-curriculum/. Accessed May 17, 2019.

Received: January 23, 2019
Accepted: July 25, 2019
Published: December 13, 2019

Copyright © 2019 Hartke et al. This is an open-access publication distributed under the terms of the Creative Commons Attribution license.
