# Supplementary material for: Building a Boot Camp: Pediatric Residency Preparatory Course Design Workshop and Tool Kit
Source: MedEdPORTAL. 2019 Dec 13;15:10860. doi: 10.15766/mep_2374-8265.10860 (PMC7010200; doi:10.15766/mep_2374-8265.10860)
Supplement: Supplementary file 1 — A. Boot Camp Workshop Presentation.pptx B. Review of Existing Boot Camp Literature.docx C. Institutional Needs Assessment Worksheet.docx D. Recommended Content List and Session Prioritization Worksheet.docx E. Schedule Worksheet and Sample Schedules.docx F. Module Design Worksheet and Planning Resources.docx G. Selected MedEdPORTAL Boot Camp Resources.docx H. Workshop Feedback Surveys.docx I. Facilitator Guide.docx [file mep-15-10860-s001.zip › A. Boot Camp Workshop Presentation.pptx]

## Slide 1
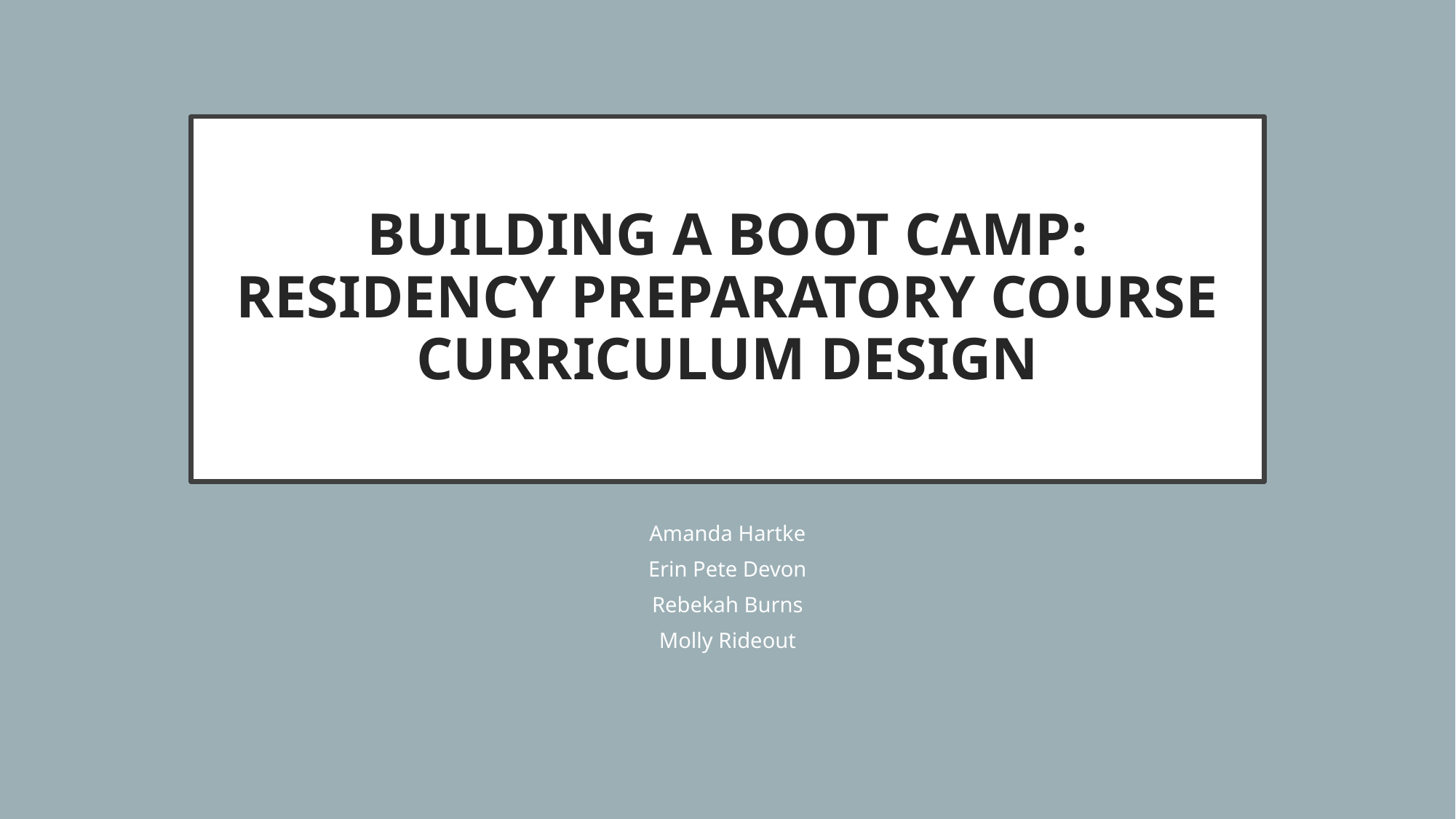

# BUILDING A BOOT CAMP: RESIDENCY PREPARATORY COURSE CURRICULUM DESIGN
Amanda Hartke
Erin Pete Devon
Rebekah Burns
Molly Rideout

## Slide 2
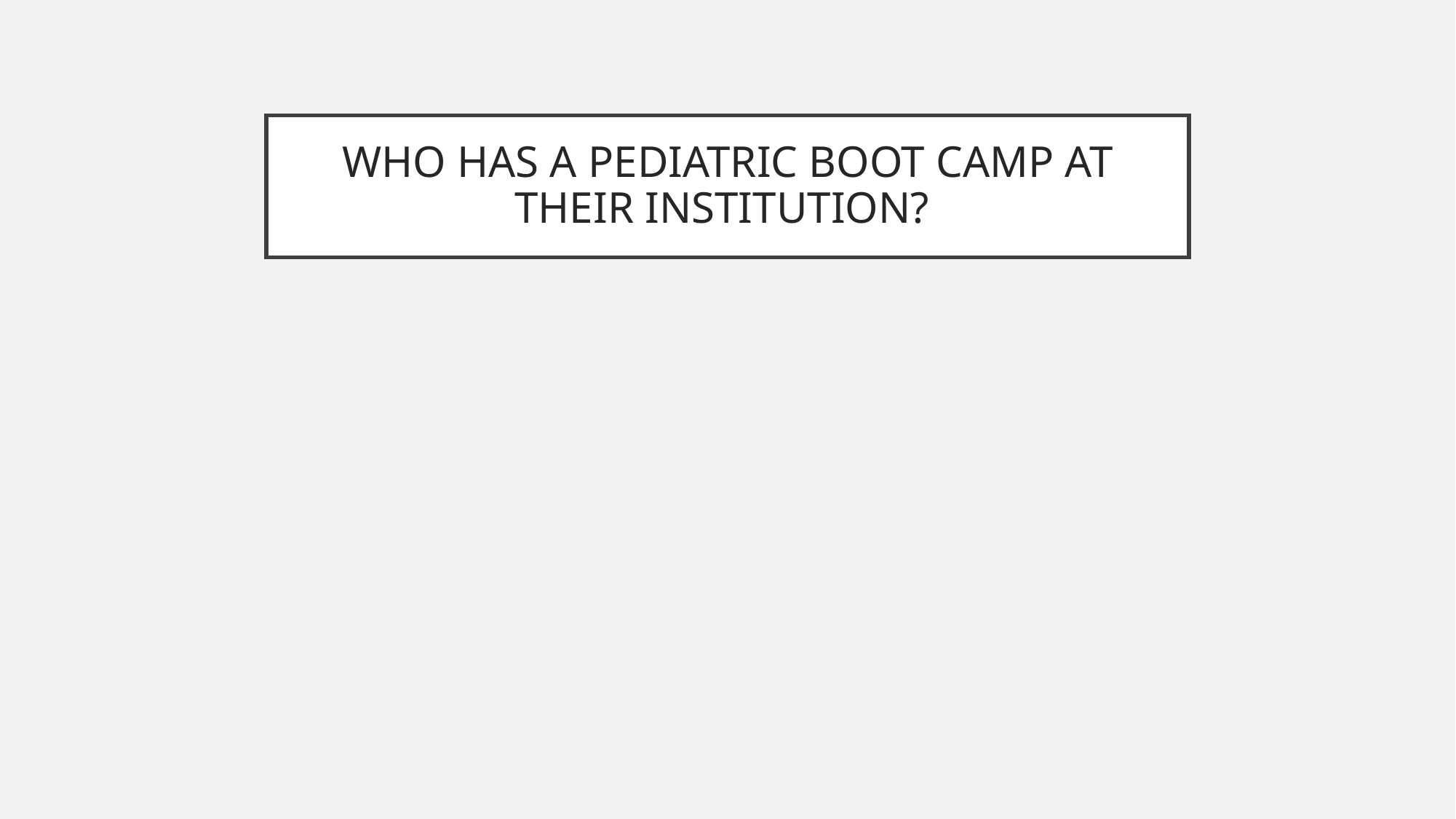

# WHO HAS A PEDIATRIC BOOT CAMP AT THEIR INSTITUTION?

## Slide 3
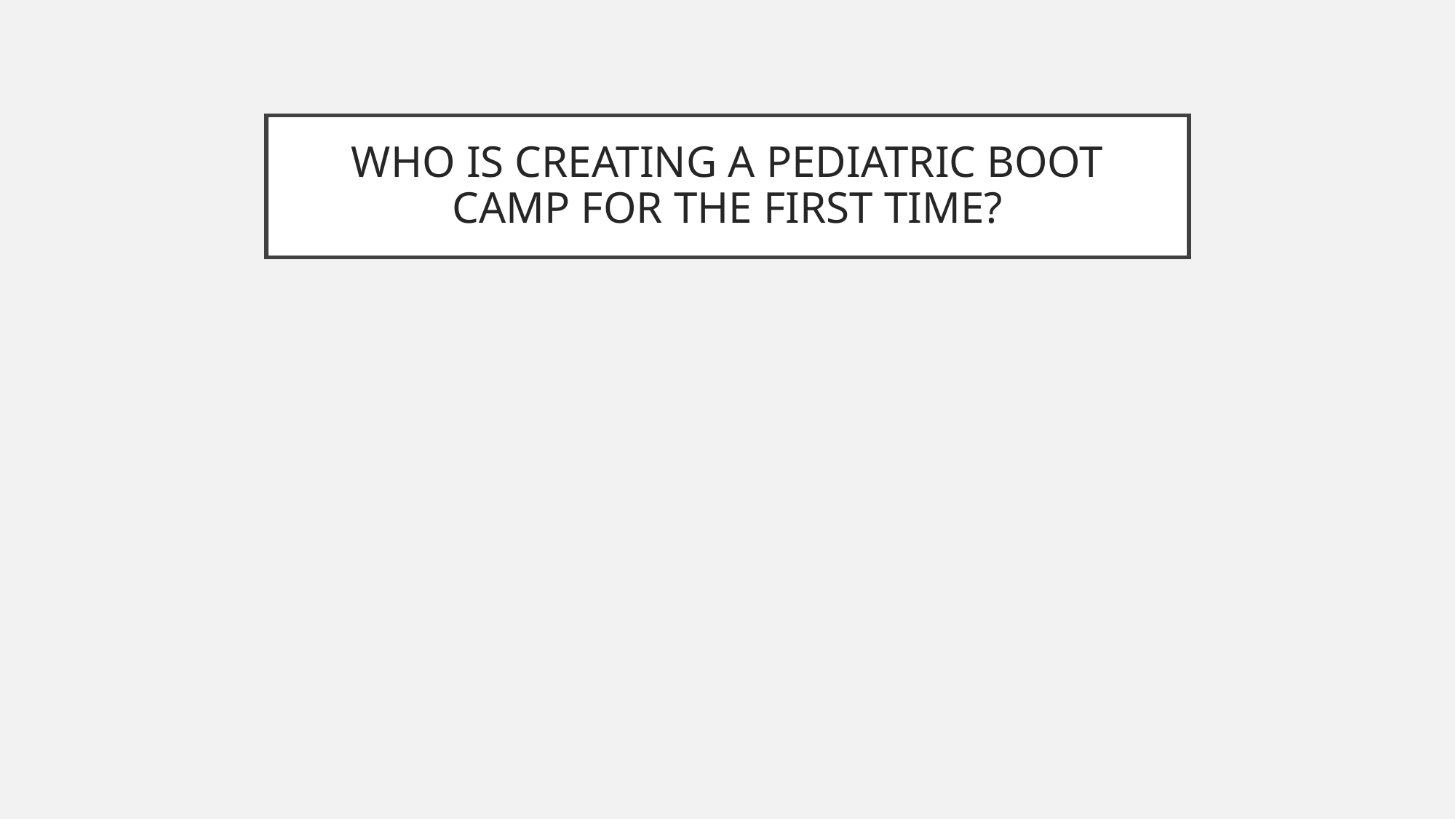

# WHO IS CREATING A PEDIATRIC BOOT CAMP FOR THE FIRST TIME?

## Slide 4
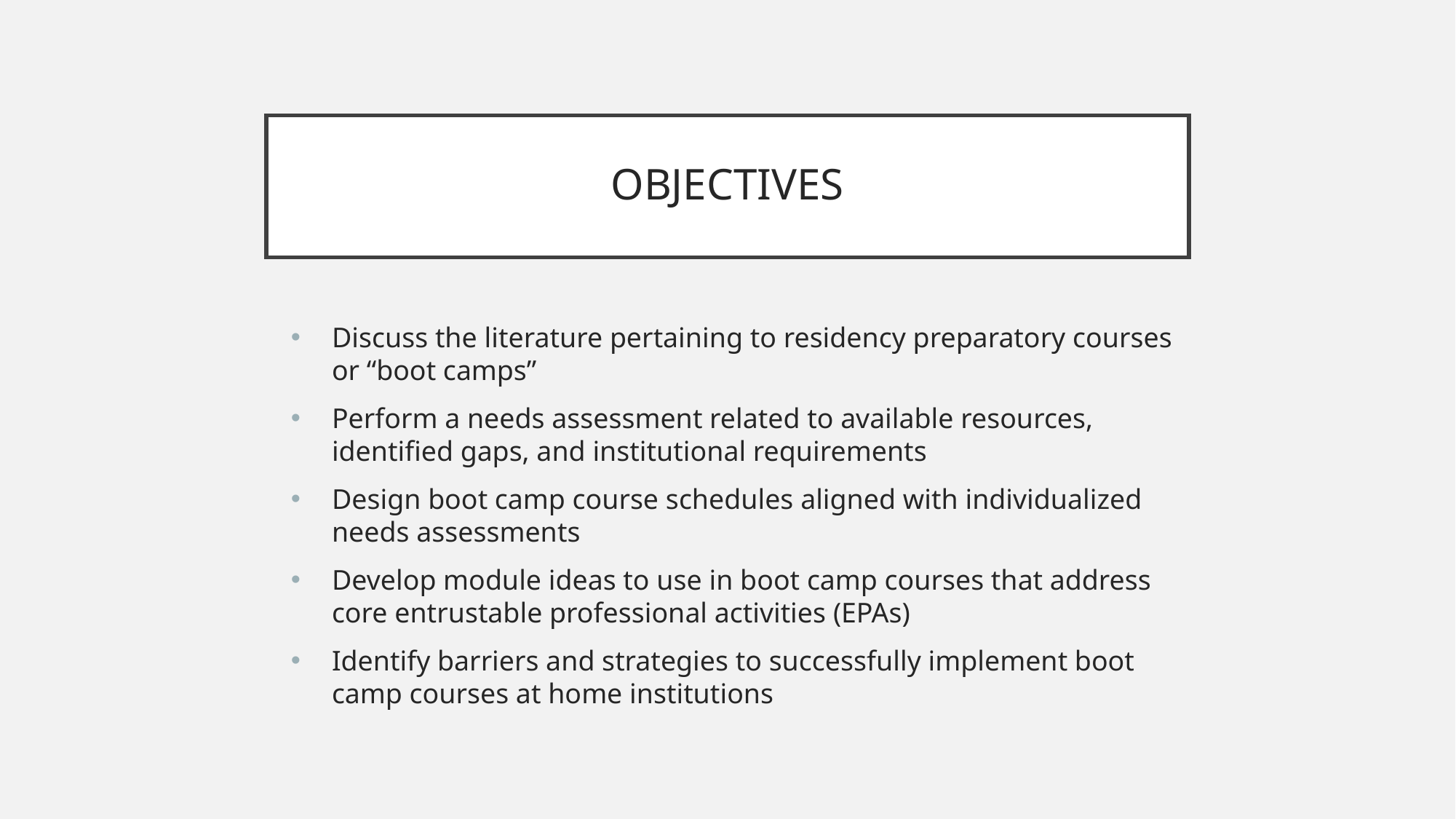

# OBJECTIVES
Discuss the literature pertaining to residency preparatory courses or “boot camps”
Perform a needs assessment related to available resources, identified gaps, and institutional requirements
Design boot camp course schedules aligned with individualized needs assessments
Develop module ideas to use in boot camp courses that address core entrustable professional activities (EPAs)
Identify barriers and strategies to successfully implement boot camp courses at home institutions

## Slide 5
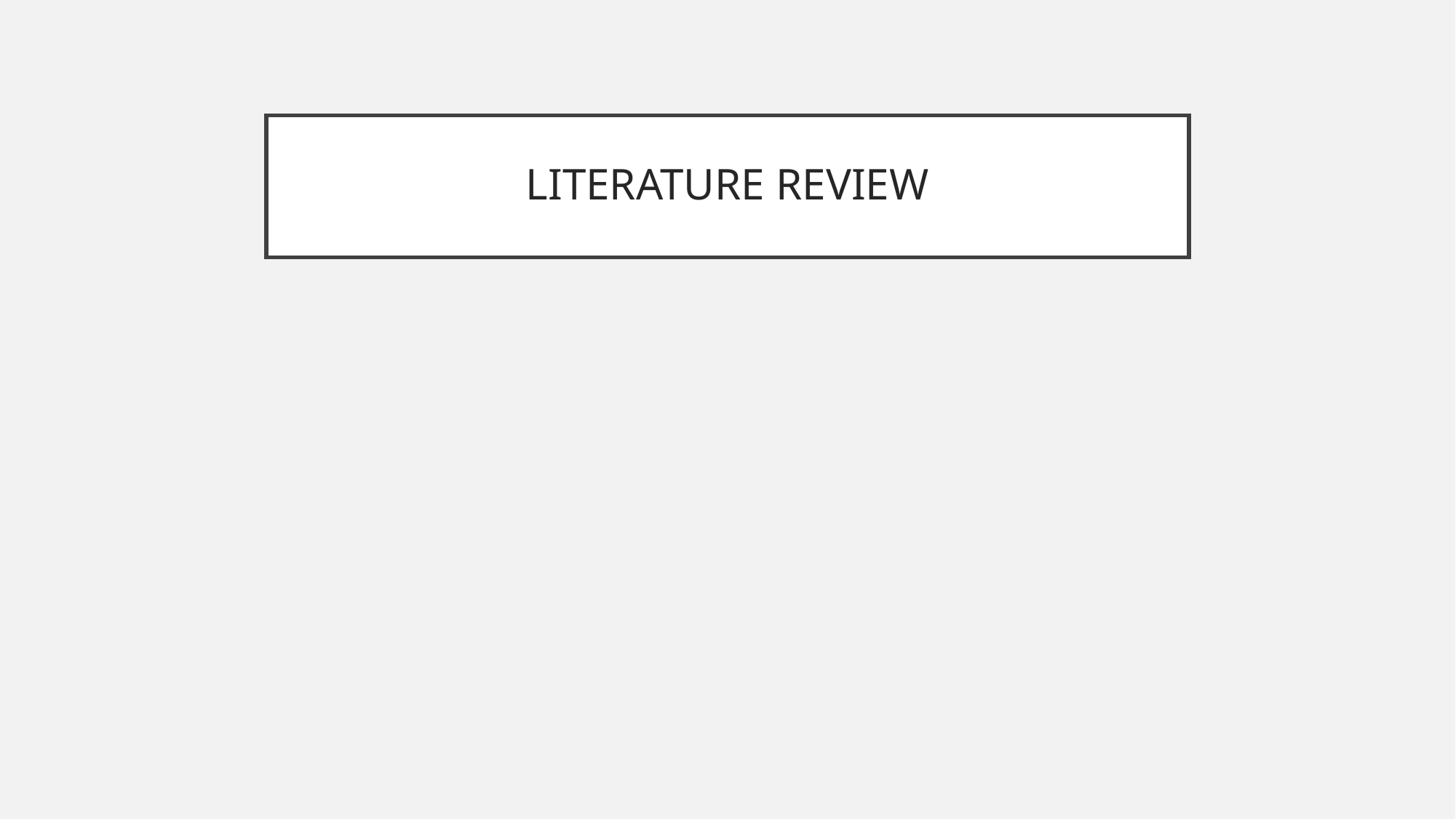

# LITERATURE REVIEW

## Slide 6
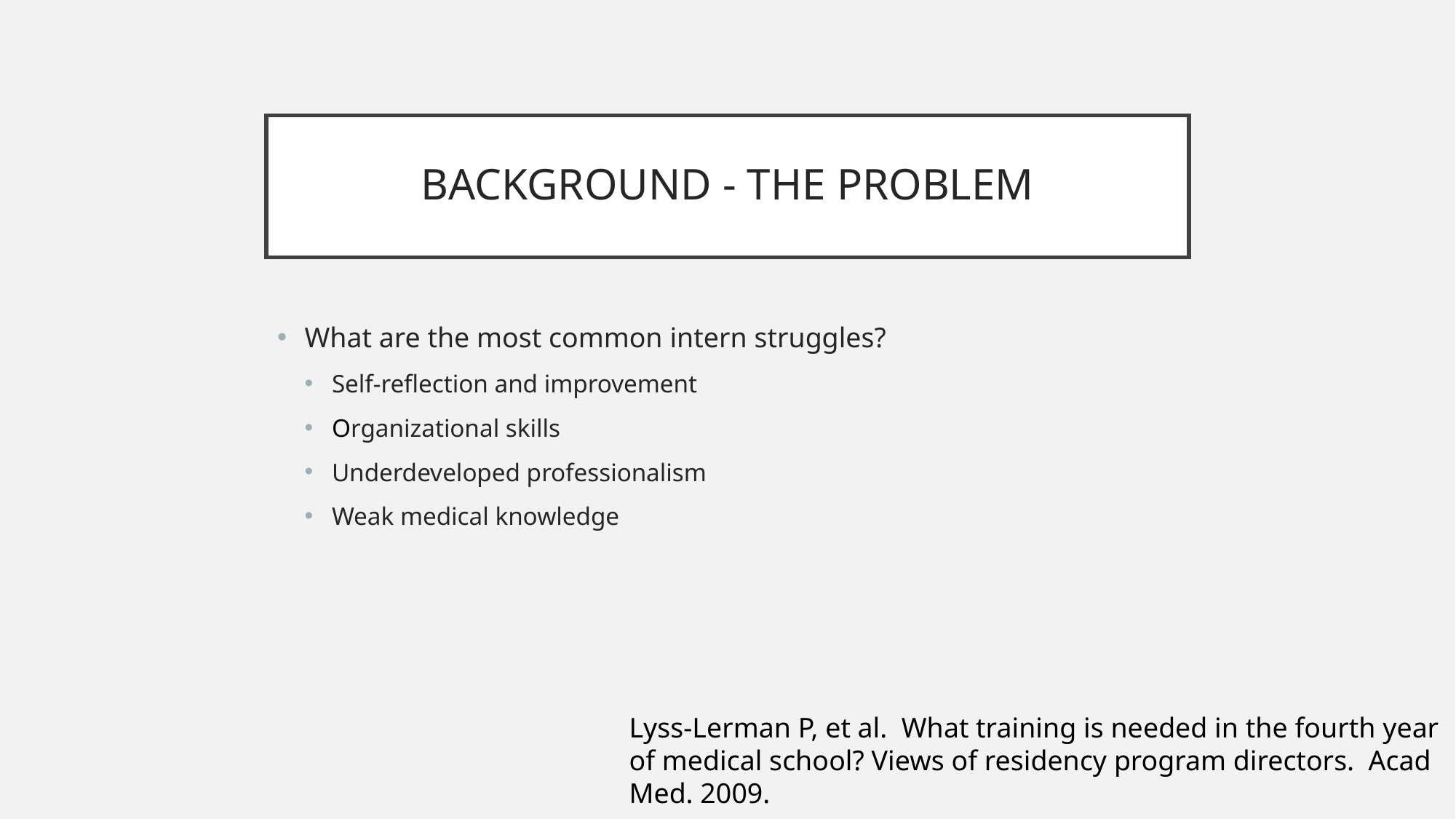

# BACKGROUND - THE PROBLEM
What are the most common intern struggles?
Self-reflection and improvement
Organizational skills
Underdeveloped professionalism
Weak medical knowledge
Lyss-Lerman P, et al. What training is needed in the fourth year of medical school? Views of residency program directors. Acad Med. 2009.

## Slide 7
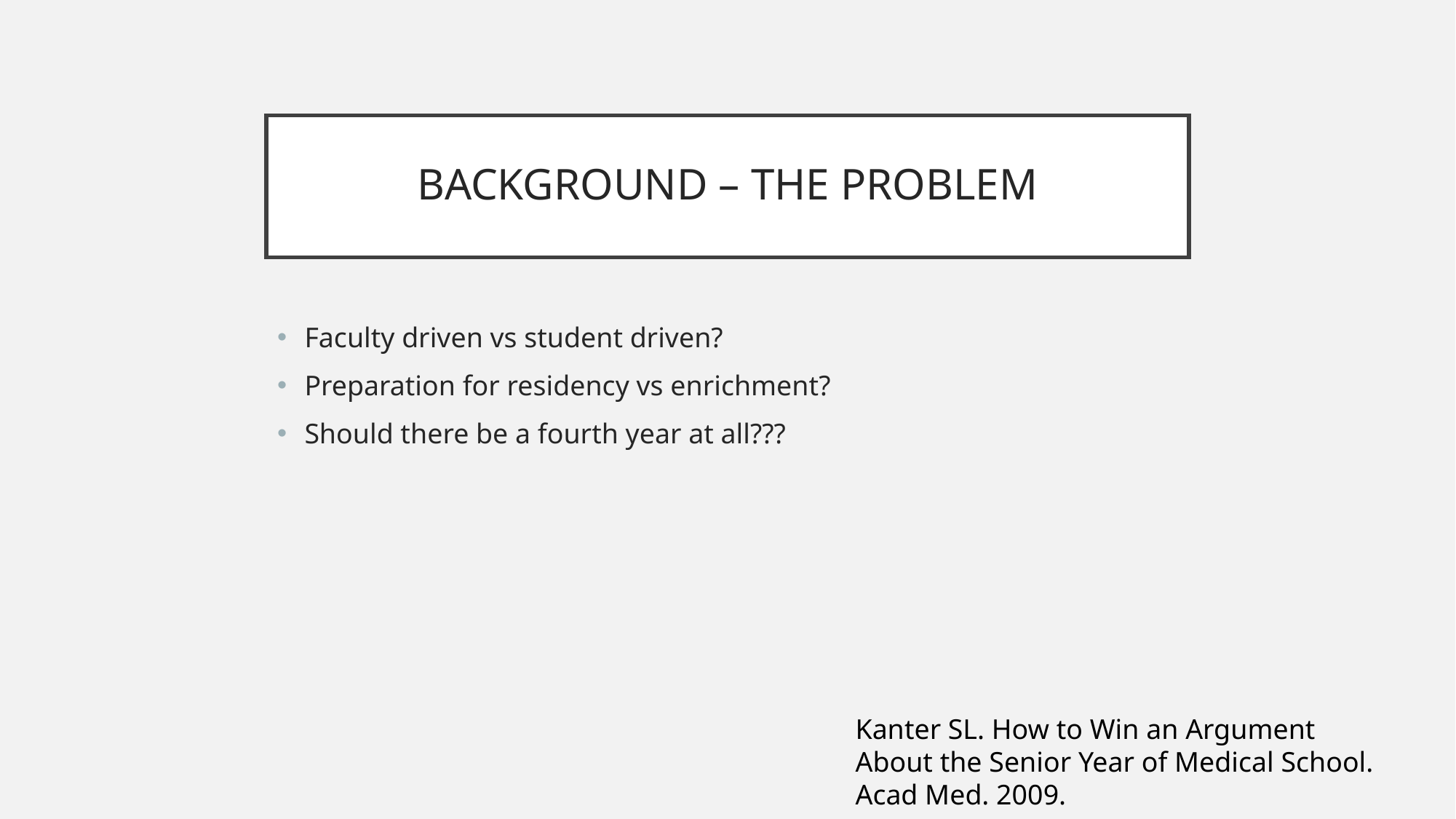

# BACKGROUND – THE PROBLEM
Faculty driven vs student driven?
Preparation for residency vs enrichment?
Should there be a fourth year at all???
Kanter SL. How to Win an Argument About the Senior Year of Medical School. Acad Med. 2009.

## Slide 8
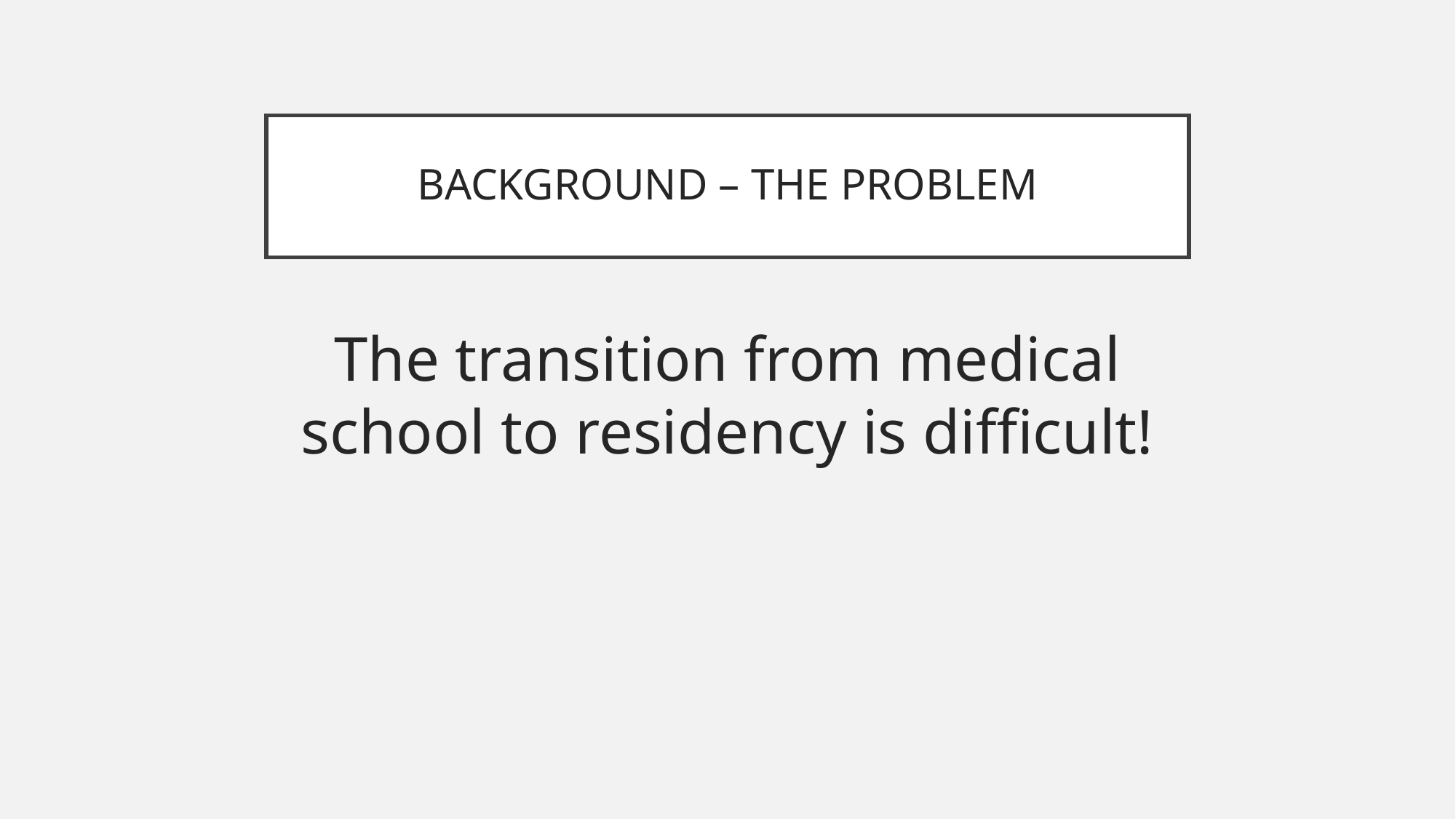

# BACKGROUND – THE PROBLEM
The transition from medical school to residency is difficult!

## Slide 9
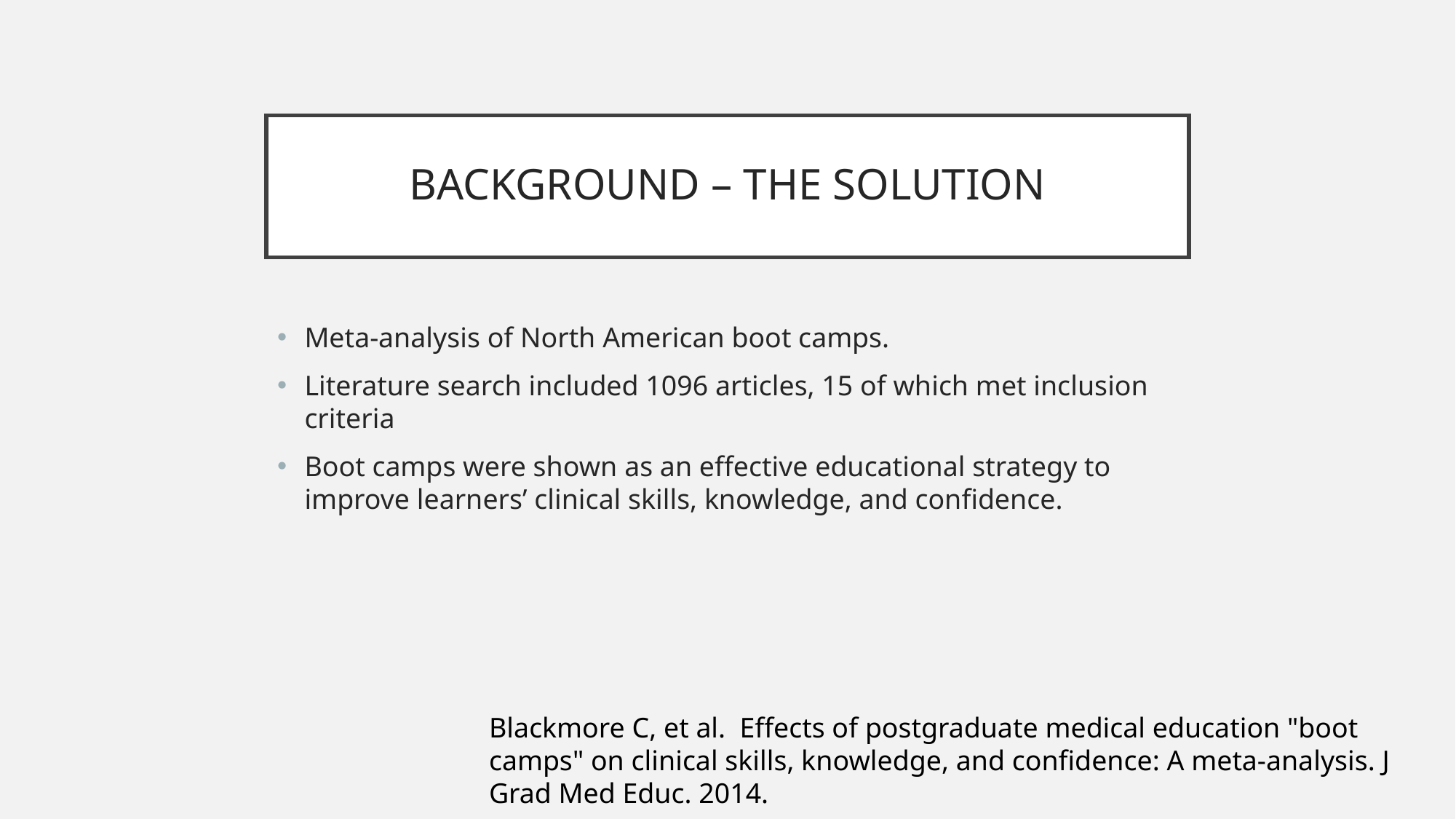

# BACKGROUND – THE SOLUTION
Meta-analysis of North American boot camps.
Literature search included 1096 articles, 15 of which met inclusion criteria
Boot camps were shown as an effective educational strategy to improve learners’ clinical skills, knowledge, and confidence.
Blackmore C, et al. Effects of postgraduate medical education "boot camps" on clinical skills, knowledge, and confidence: A meta-analysis. J Grad Med Educ. 2014.

## Slide 10
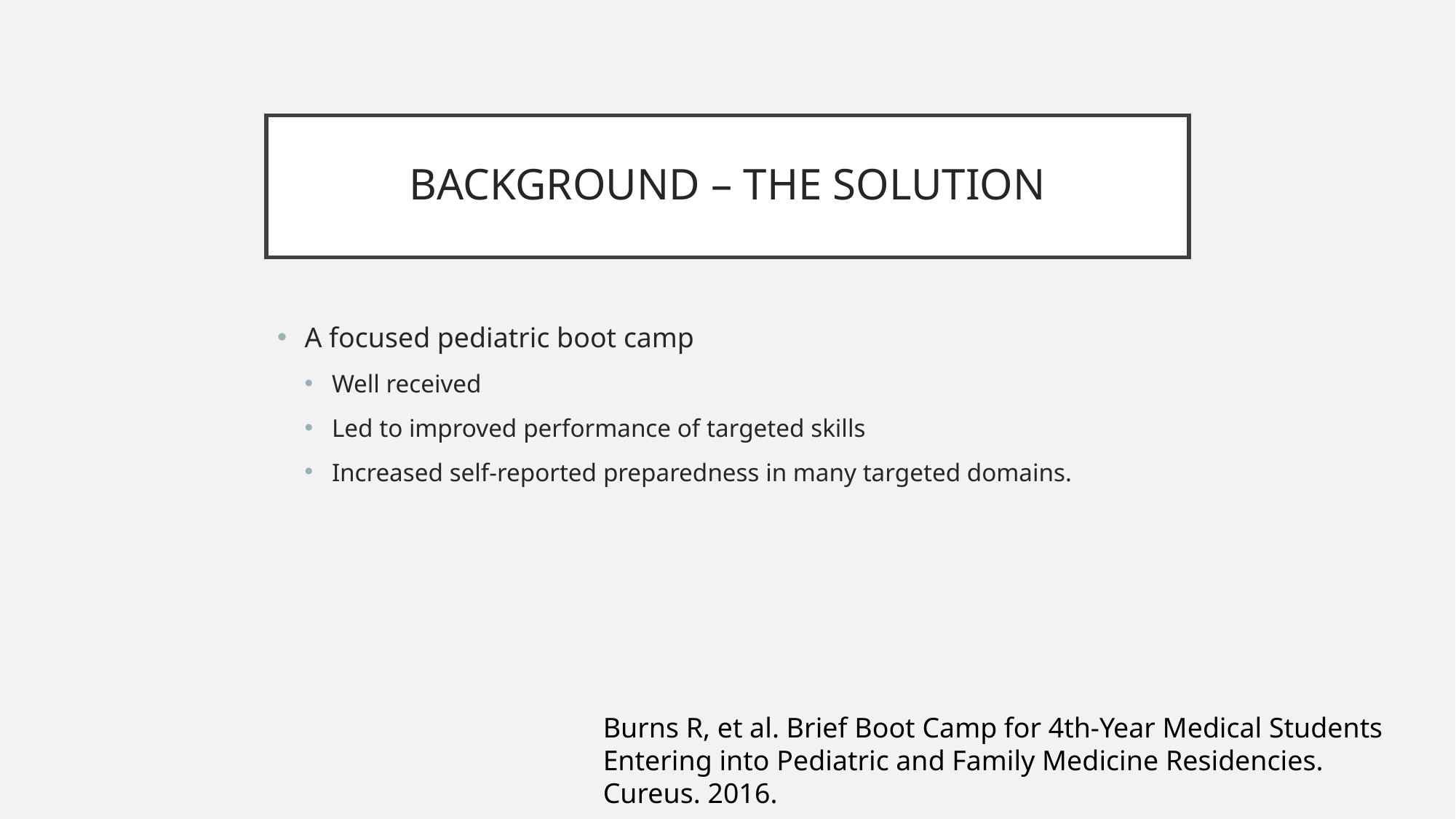

# BACKGROUND – THE SOLUTION
A focused pediatric boot camp
Well received
Led to improved performance of targeted skills
Increased self-reported preparedness in many targeted domains.
Burns R, et al. Brief Boot Camp for 4th-Year Medical Students Entering into Pediatric and Family Medicine Residencies. Cureus. 2016.

## Slide 11
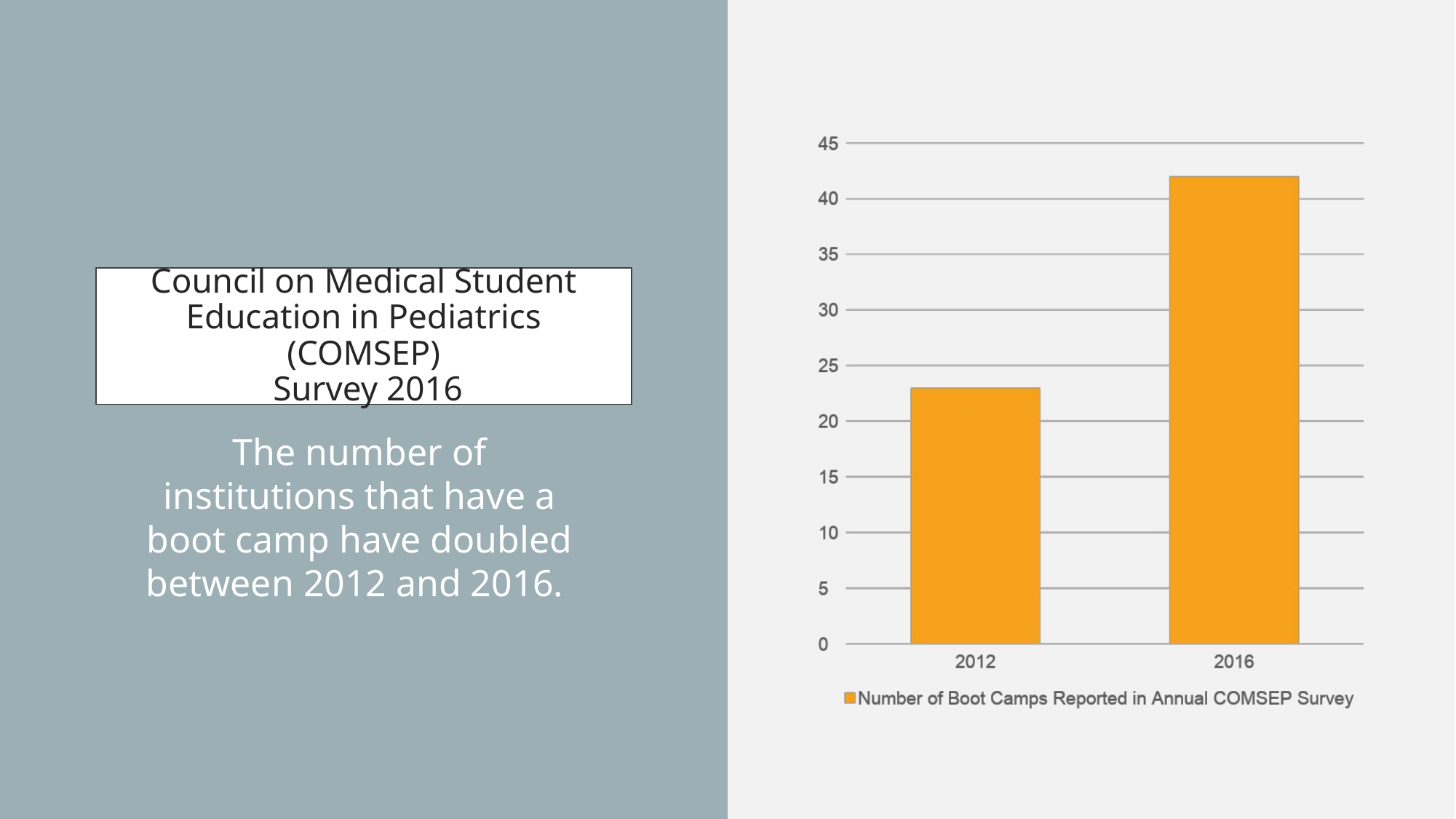

# Council on Medical Student Education in Pediatrics (COMSEP) Survey 2016
The number of institutions that have a boot camp have doubled between 2012 and 2016.

## Slide 12
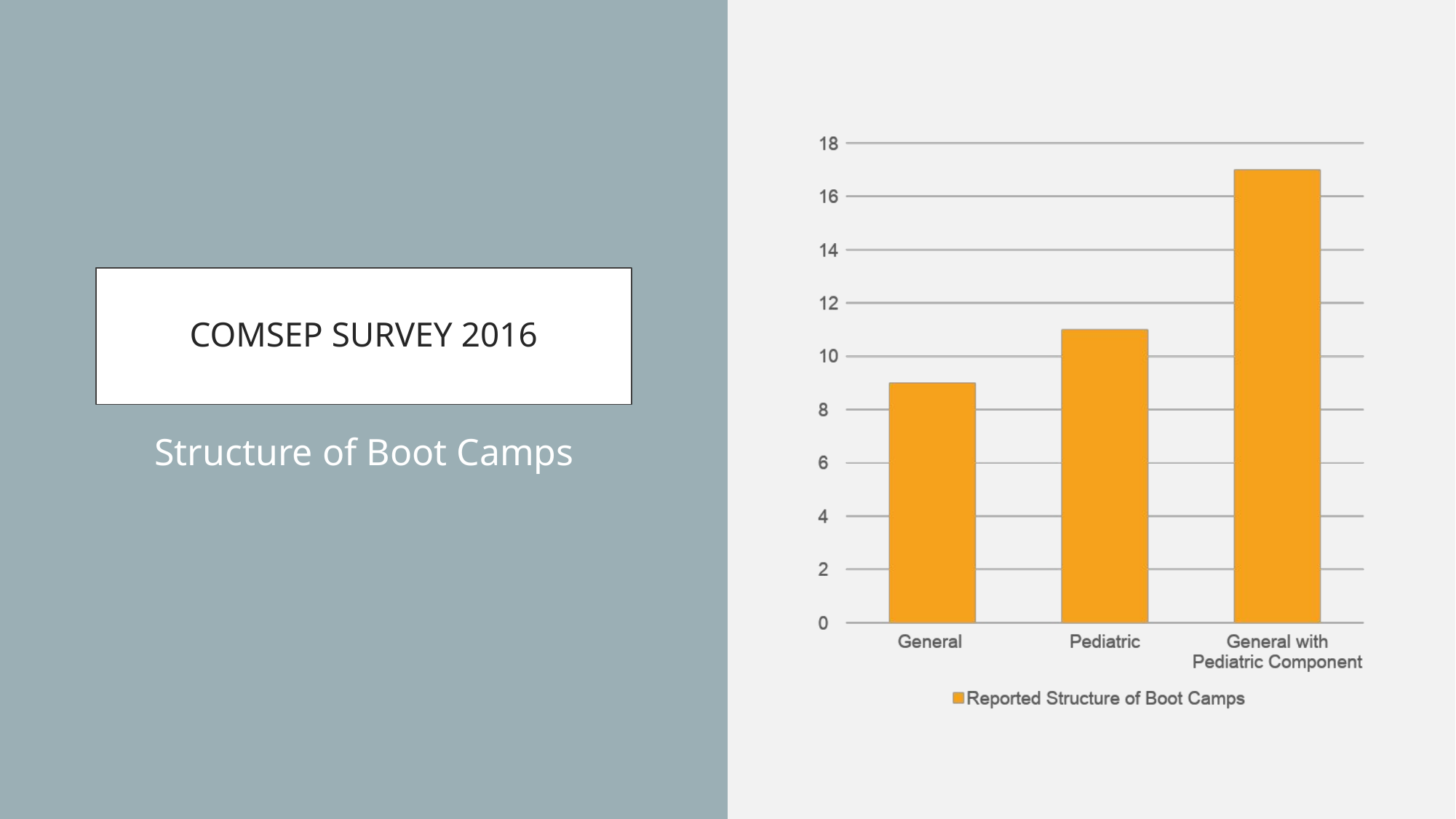

# COMSEP SURVEY 2016
 Structure of Boot Camps

## Slide 13
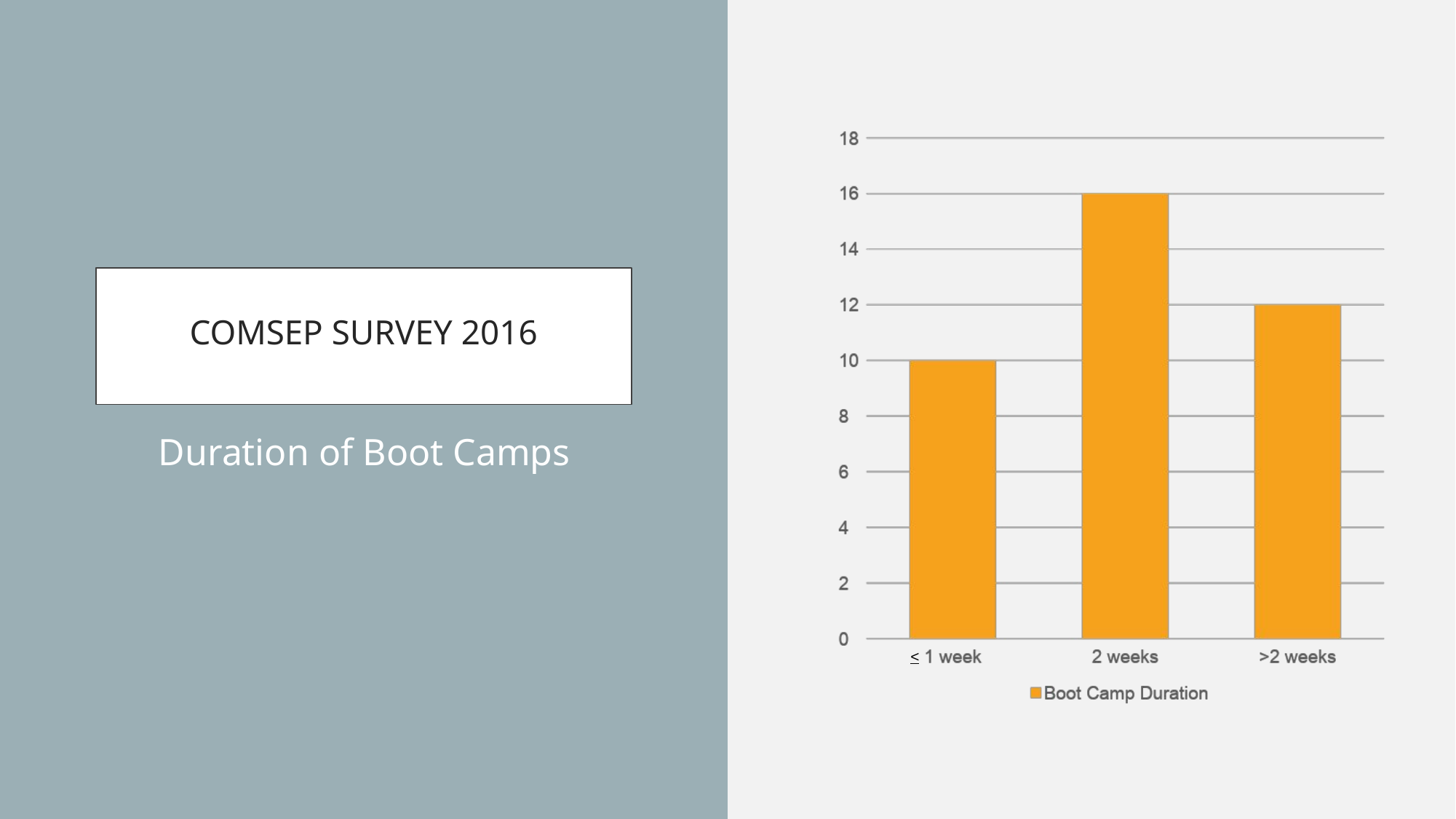

<
# COMSEP SURVEY 2016
 Duration of Boot Camps

## Slide 14
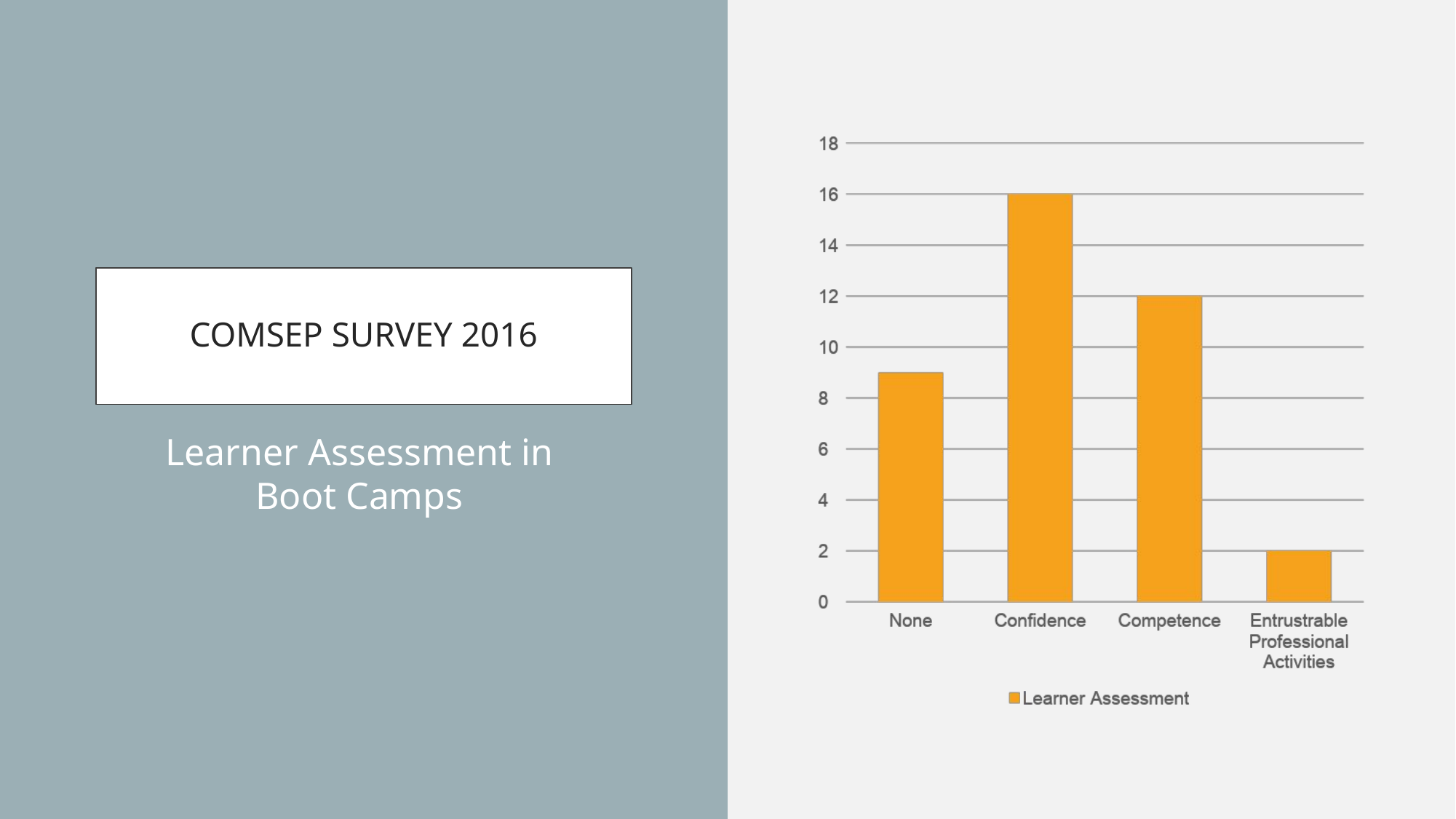

# COMSEP SURVEY 2016
Learner Assessment in Boot Camps

## Slide 15
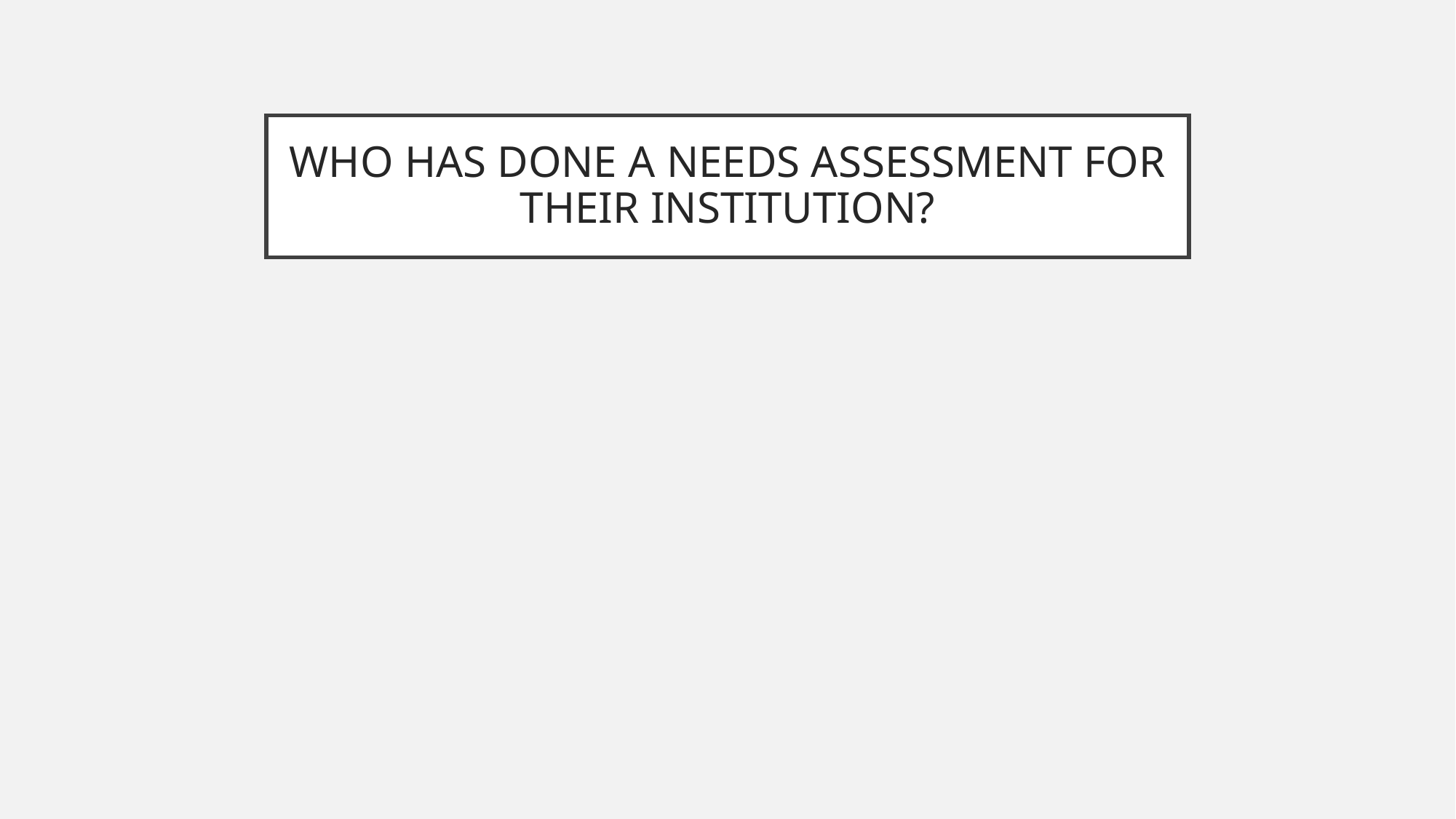

# WHO HAS DONE A NEEDS ASSESSMENT FOR THEIR INSTITUTION?

## Slide 16
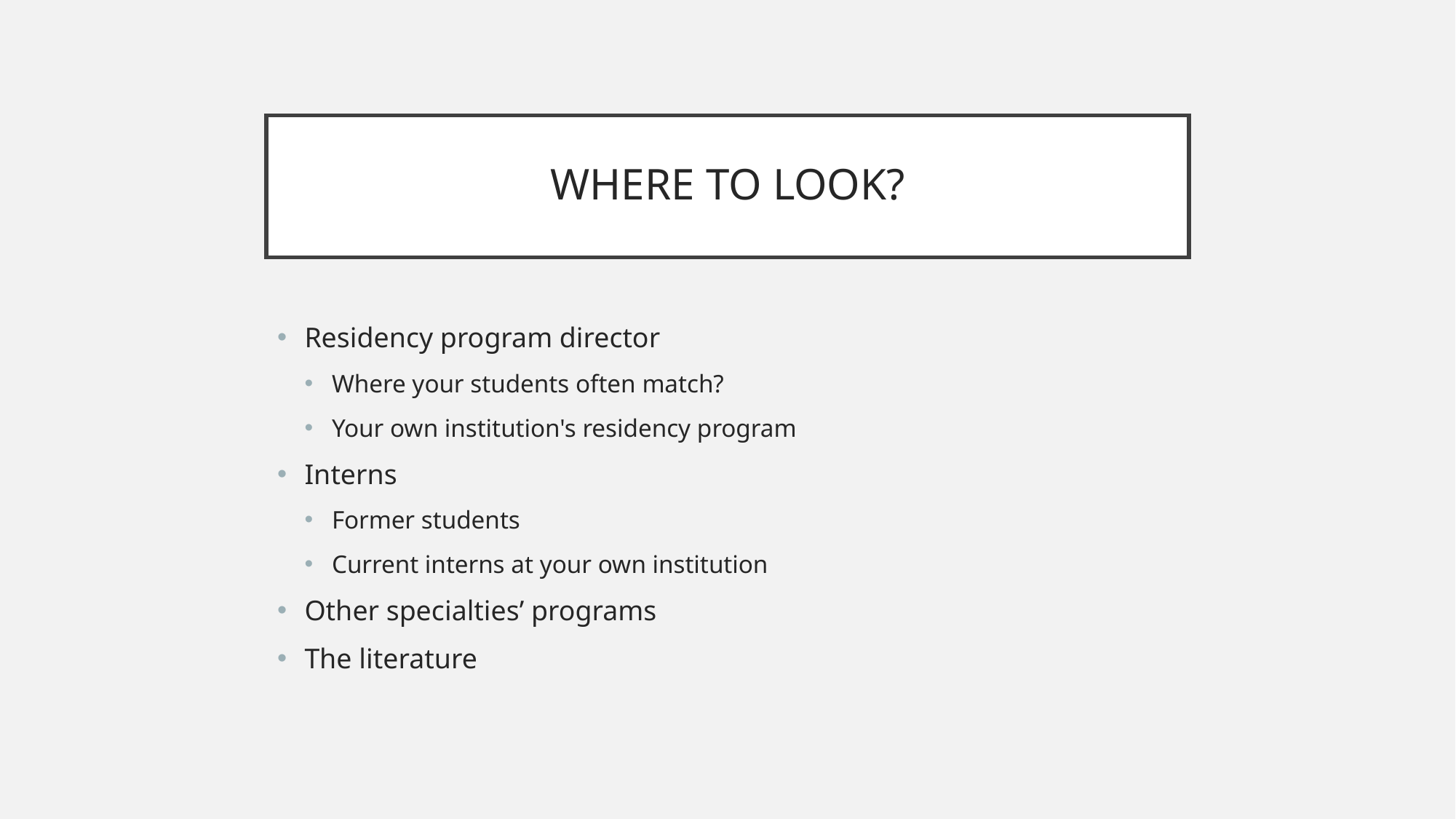

# WHERE TO LOOK?
Residency program director
Where your students often match?
Your own institution's residency program
Interns
Former students
Current interns at your own institution
Other specialties’ programs
The literature

## Slide 17
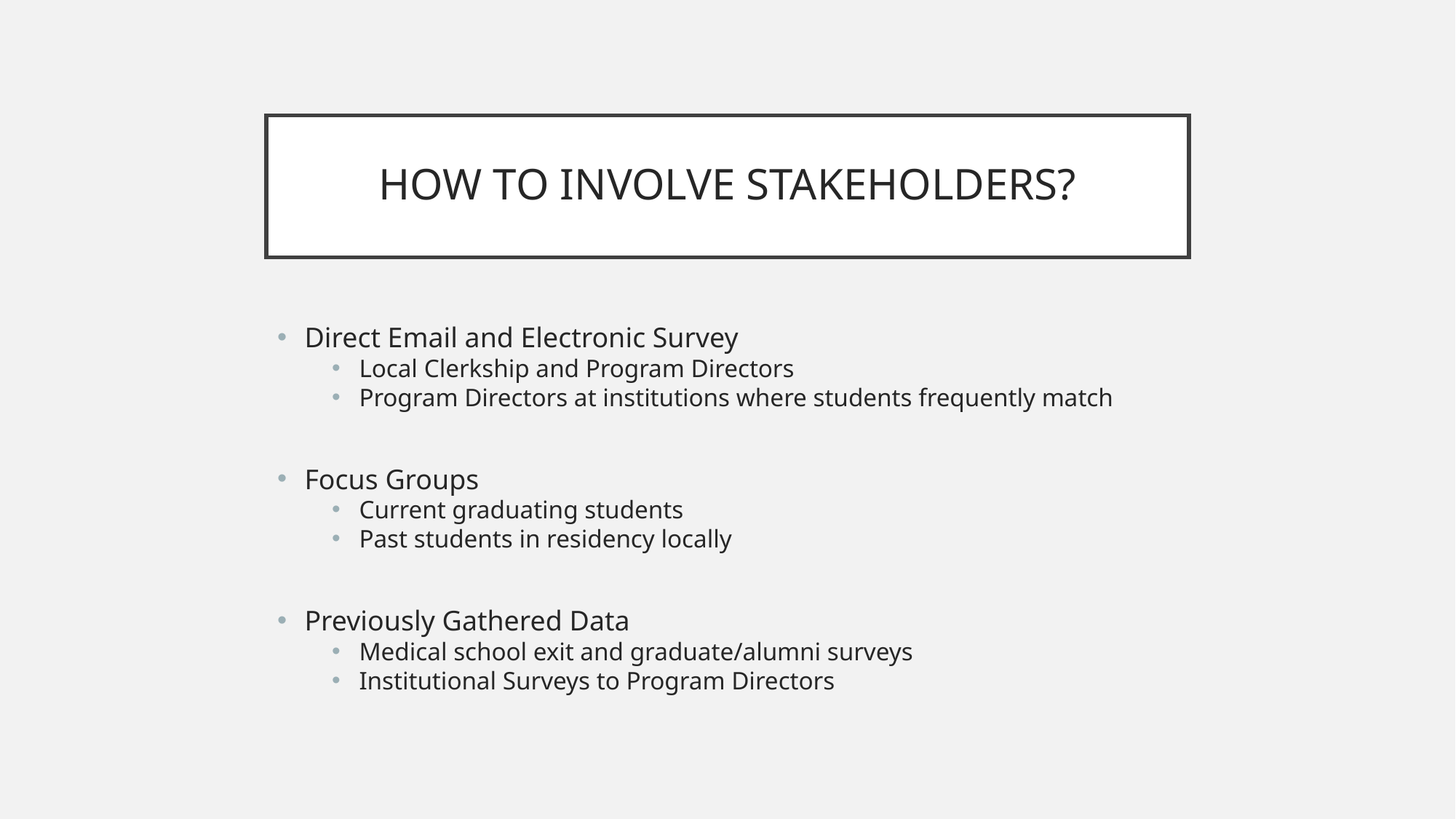

# HOW TO INVOLVE STAKEHOLDERS?
Direct Email and Electronic Survey
Local Clerkship and Program Directors
Program Directors at institutions where students frequently match
Focus Groups
Current graduating students
Past students in residency locally
Previously Gathered Data
Medical school exit and graduate/alumni surveys
Institutional Surveys to Program Directors

## Slide 18
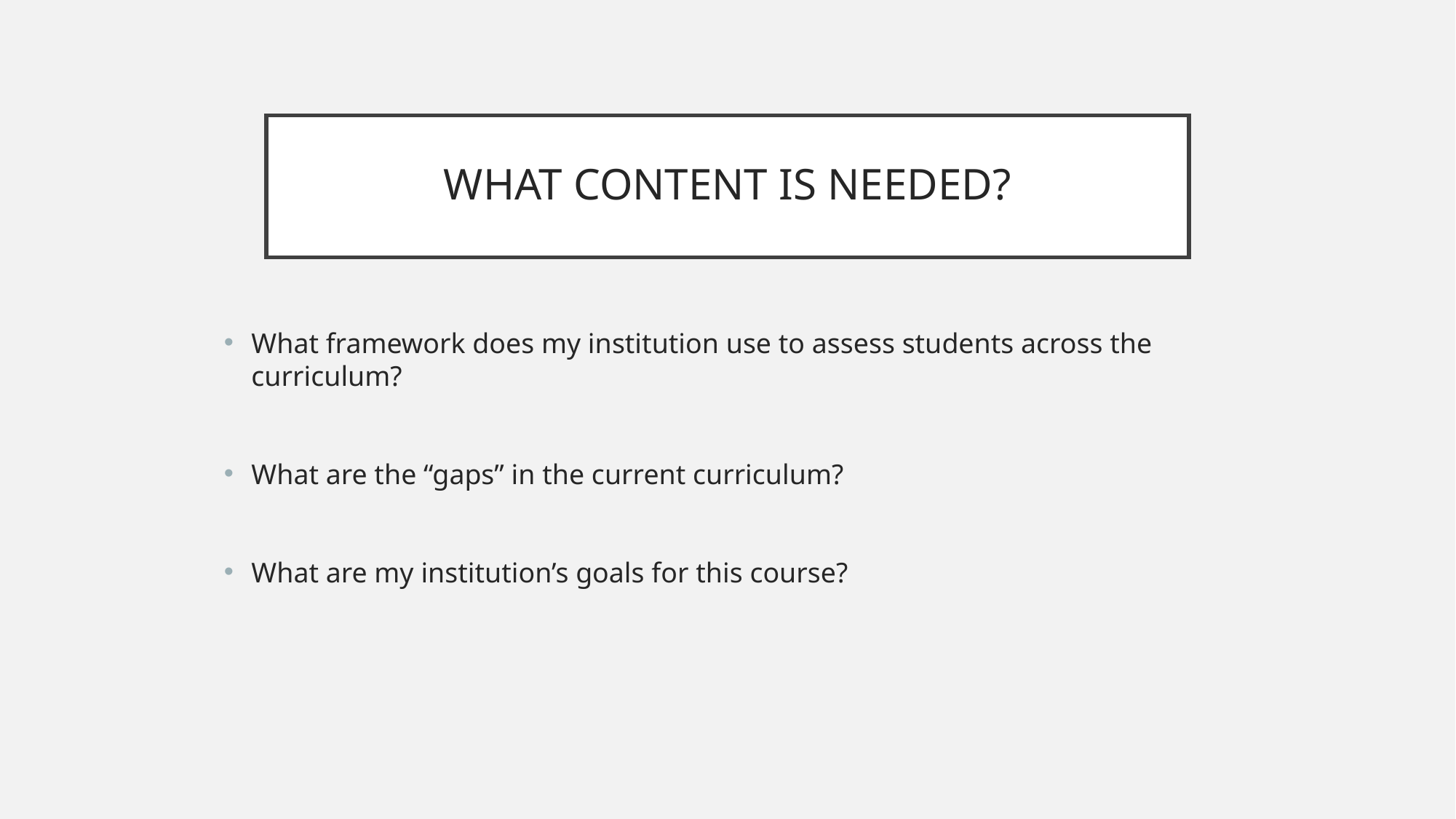

# WHAT CONTENT IS NEEDED?
What framework does my institution use to assess students across the curriculum?
What are the “gaps” in the current curriculum?
What are my institution’s goals for this course?

## Slide 19
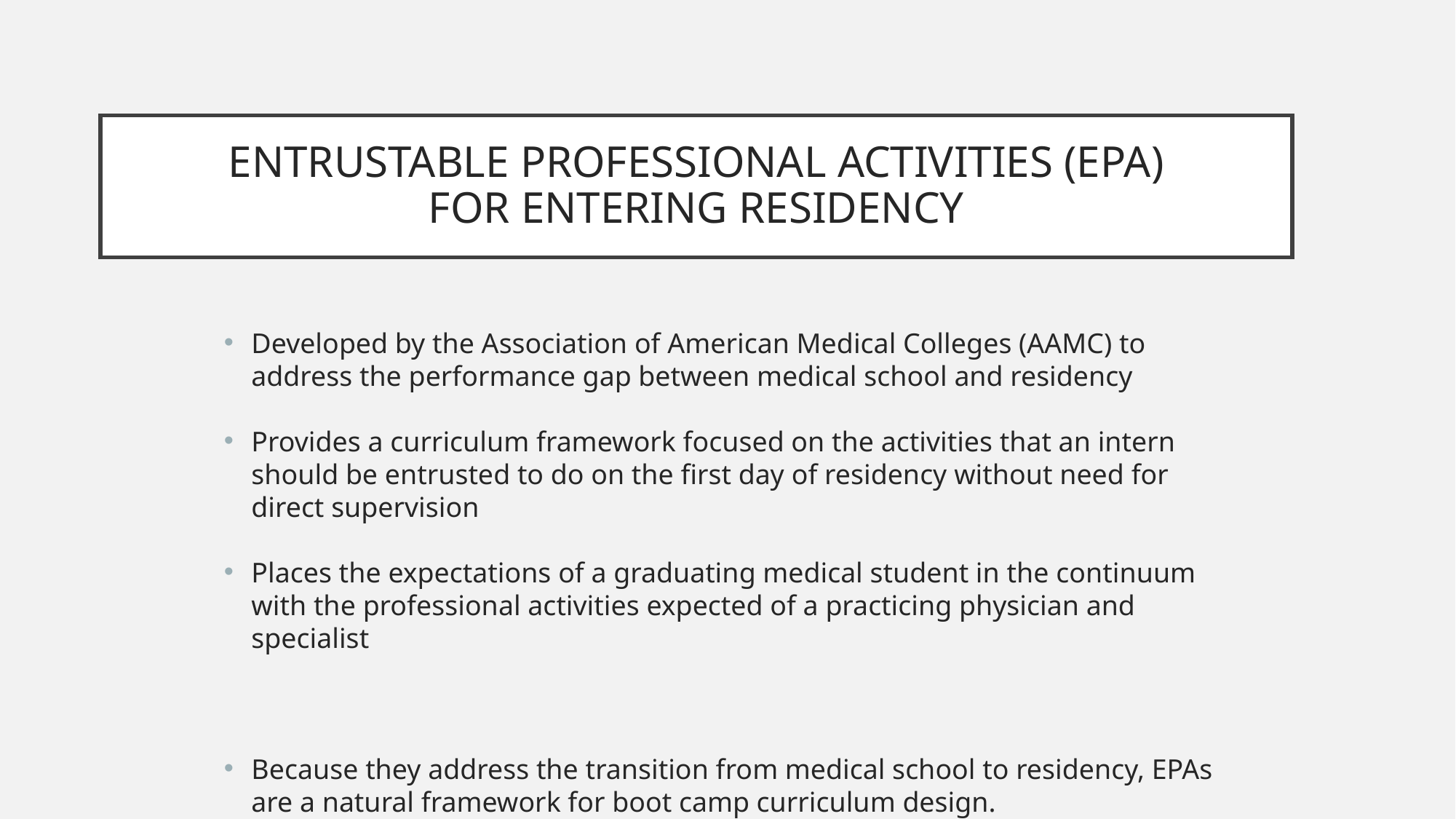

# ENTRUSTABLE PROFESSIONAL ACTIVITIES (EPA) FOR ENTERING RESIDENCY
Developed by the Association of American Medical Colleges (AAMC) to address the performance gap between medical school and residency
Provides a curriculum framework focused on the activities that an intern should be entrusted to do on the first day of residency without need for direct supervision
Places the expectations of a graduating medical student in the continuum with the professional activities expected of a practicing physician and specialist
Because they address the transition from medical school to residency, EPAs are a natural framework for boot camp curriculum design.

## Slide 20
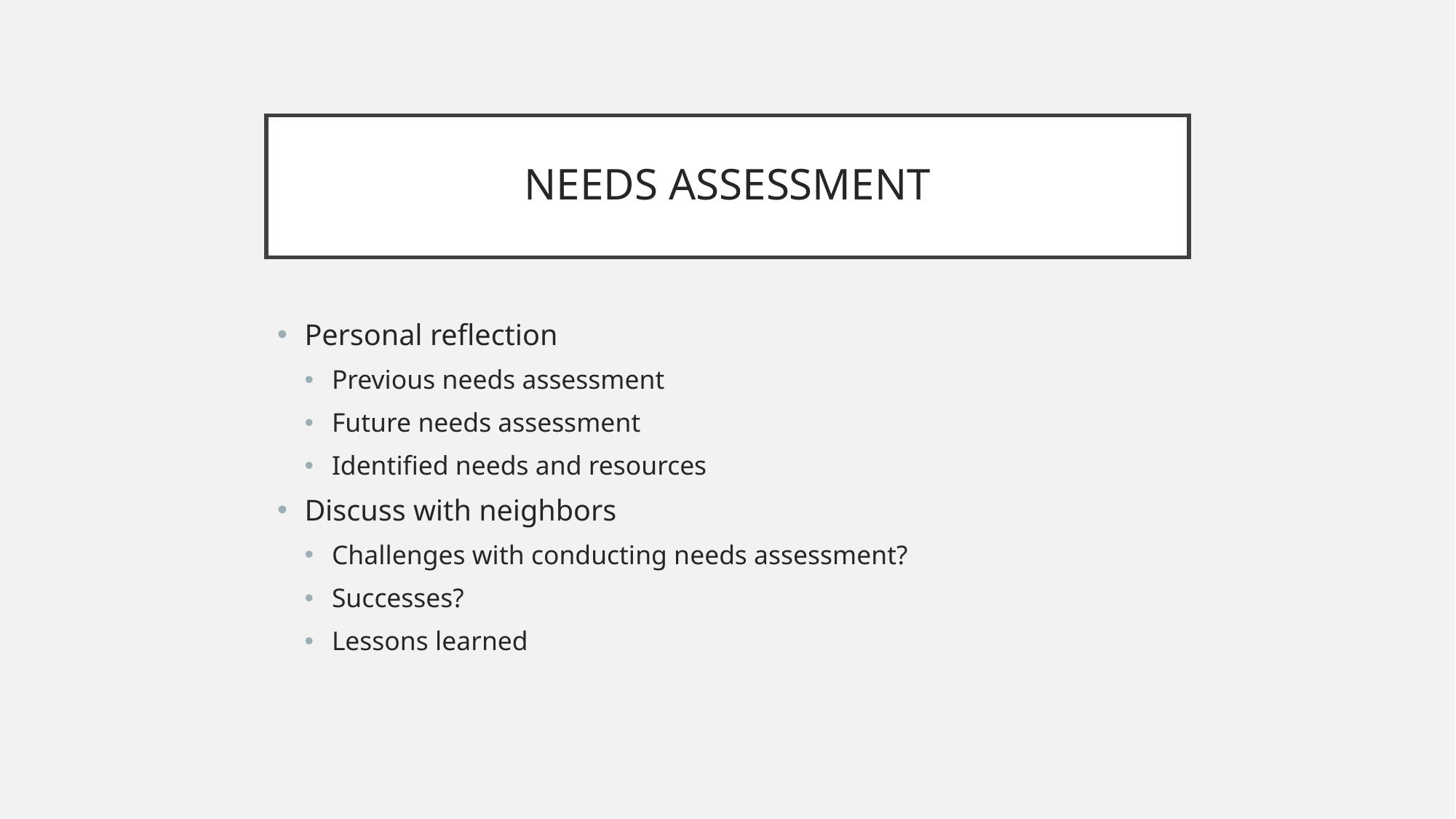

# NEEDS ASSESSMENT
Personal reflection
Previous needs assessment
Future needs assessment
Identified needs and resources
Discuss with neighbors
Challenges with conducting needs assessment?
Successes?
Lessons learned

## Slide 21
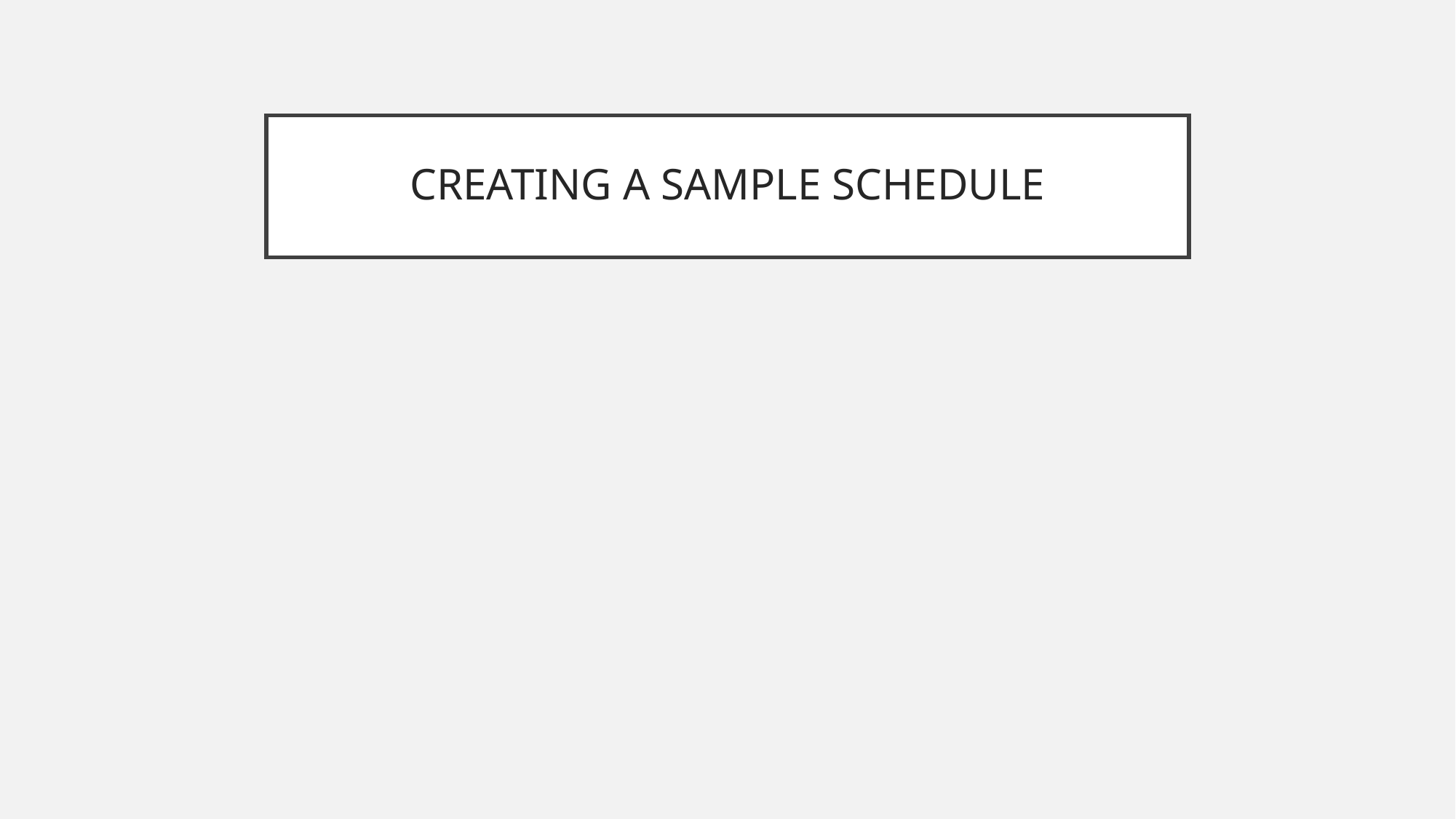

# CREATING A SAMPLE SCHEDULE

## Slide 22
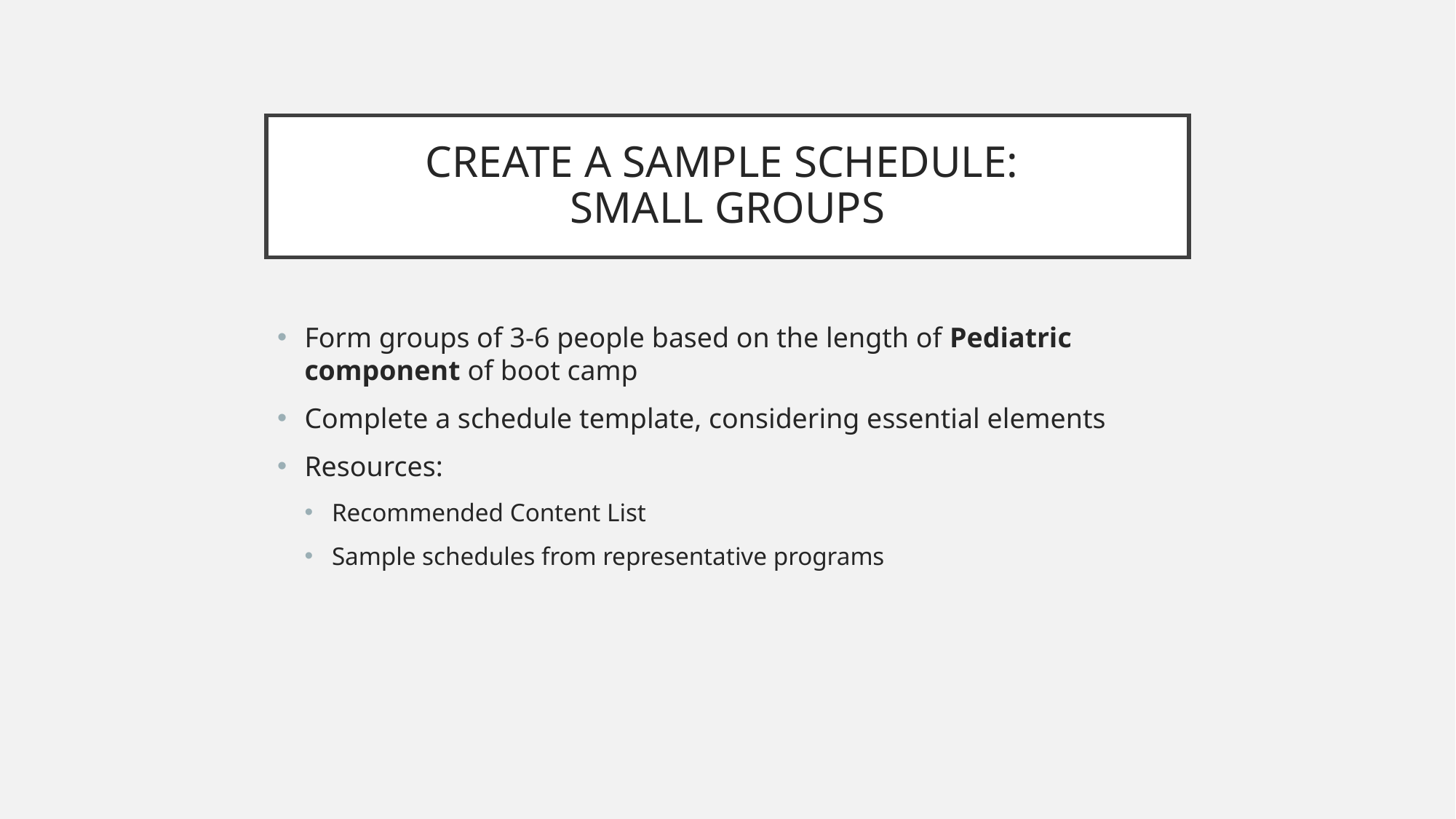

# CREATE A SAMPLE SCHEDULE: SMALL GROUPS
Form groups of 3-6 people based on the length of Pediatric component of boot camp
Complete a schedule template, considering essential elements
Resources:
Recommended Content List
Sample schedules from representative programs

## Slide 23
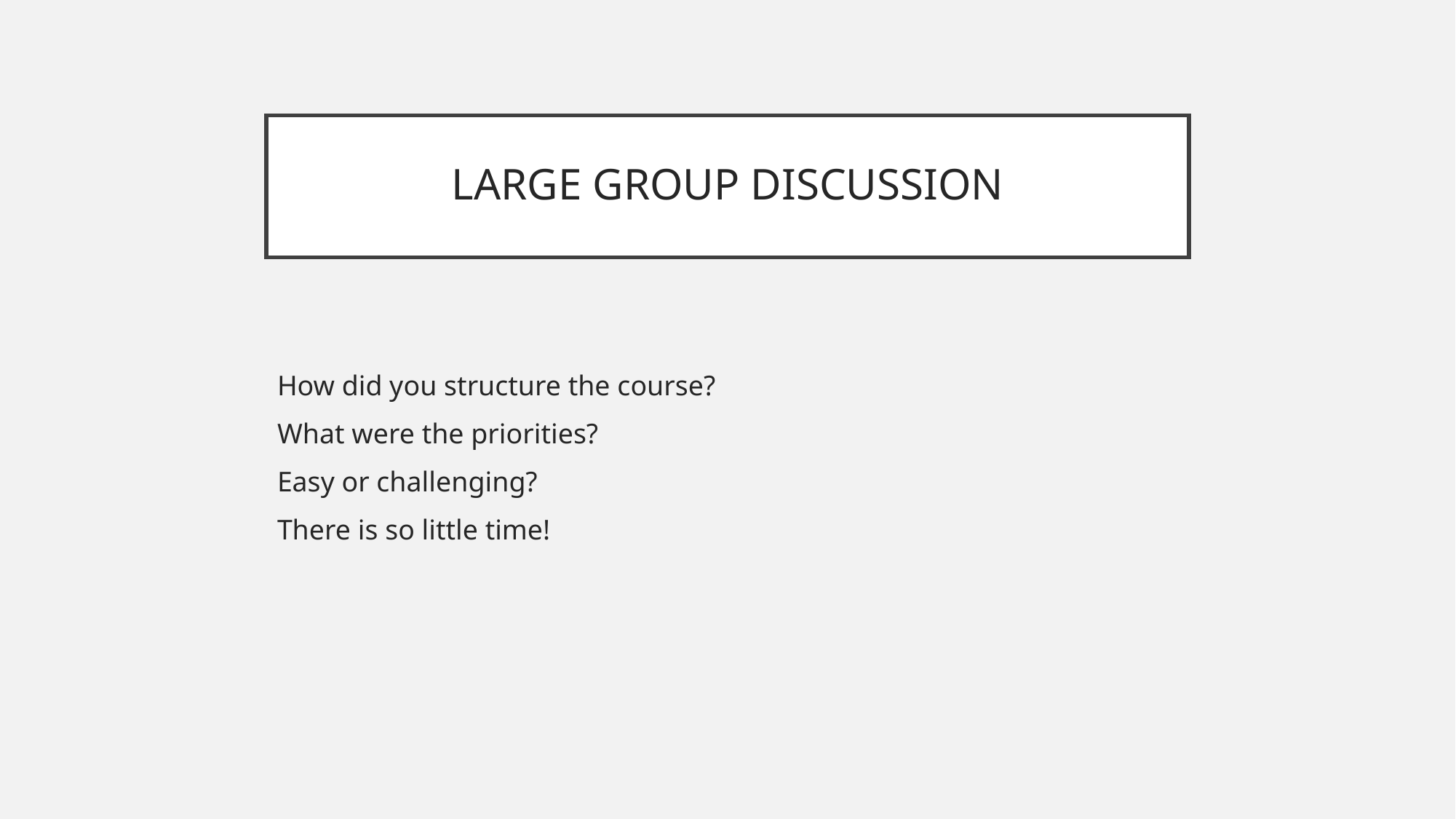

# LARGE GROUP DISCUSSION
How did you structure the course?
What were the priorities?
Easy or challenging?
There is so little time!

## Slide 24
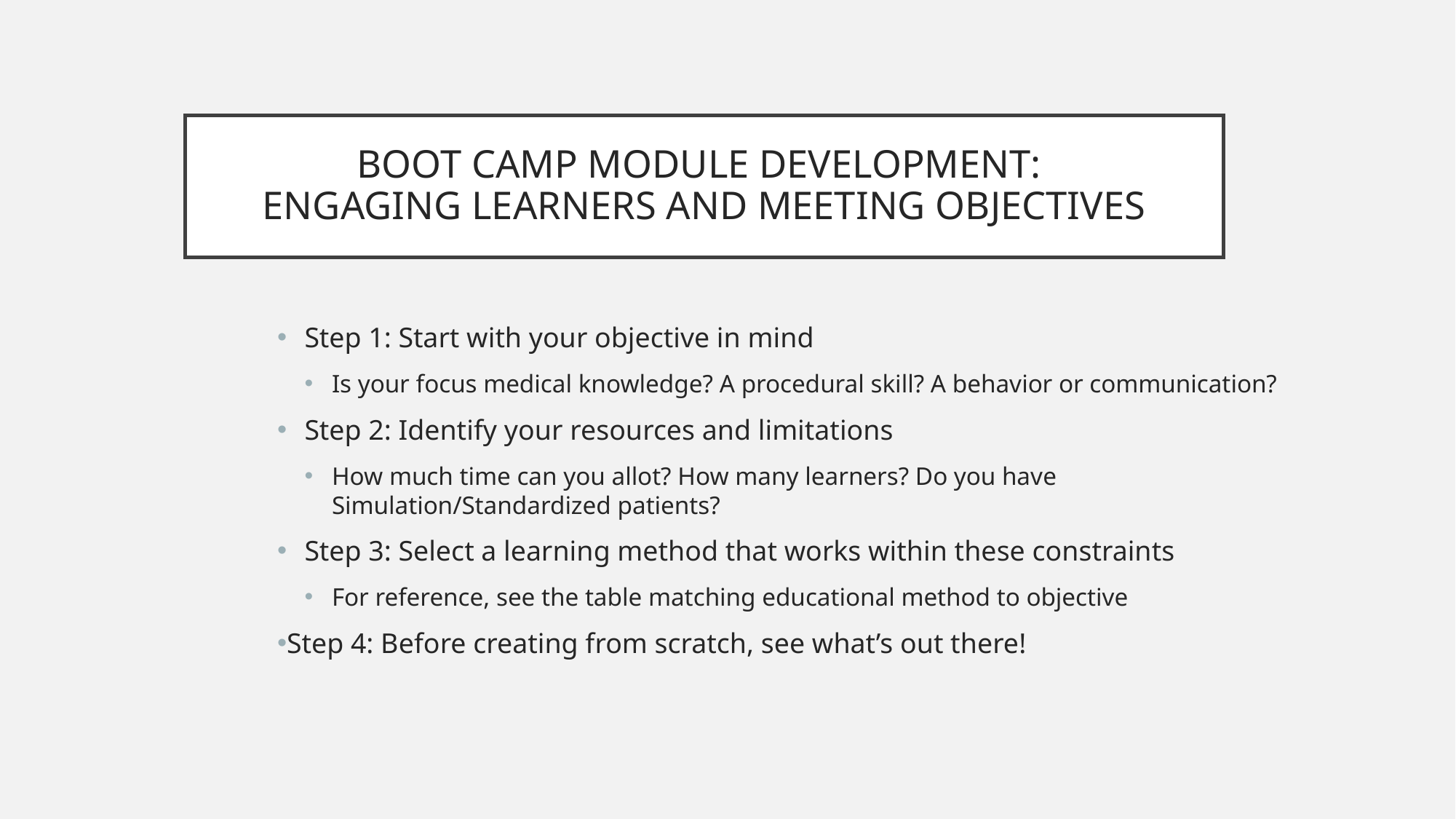

# BOOT CAMP MODULE DEVELOPMENT: ENGAGING LEARNERS AND MEETING OBJECTIVES
Step 1: Start with your objective in mind
Is your focus medical knowledge? A procedural skill? A behavior or communication?
Step 2: Identify your resources and limitations
How much time can you allot? How many learners? Do you have Simulation/Standardized patients?
Step 3: Select a learning method that works within these constraints
For reference, see the table matching educational method to objective
Step 4: Before creating from scratch, see what’s out there!

## Slide 25
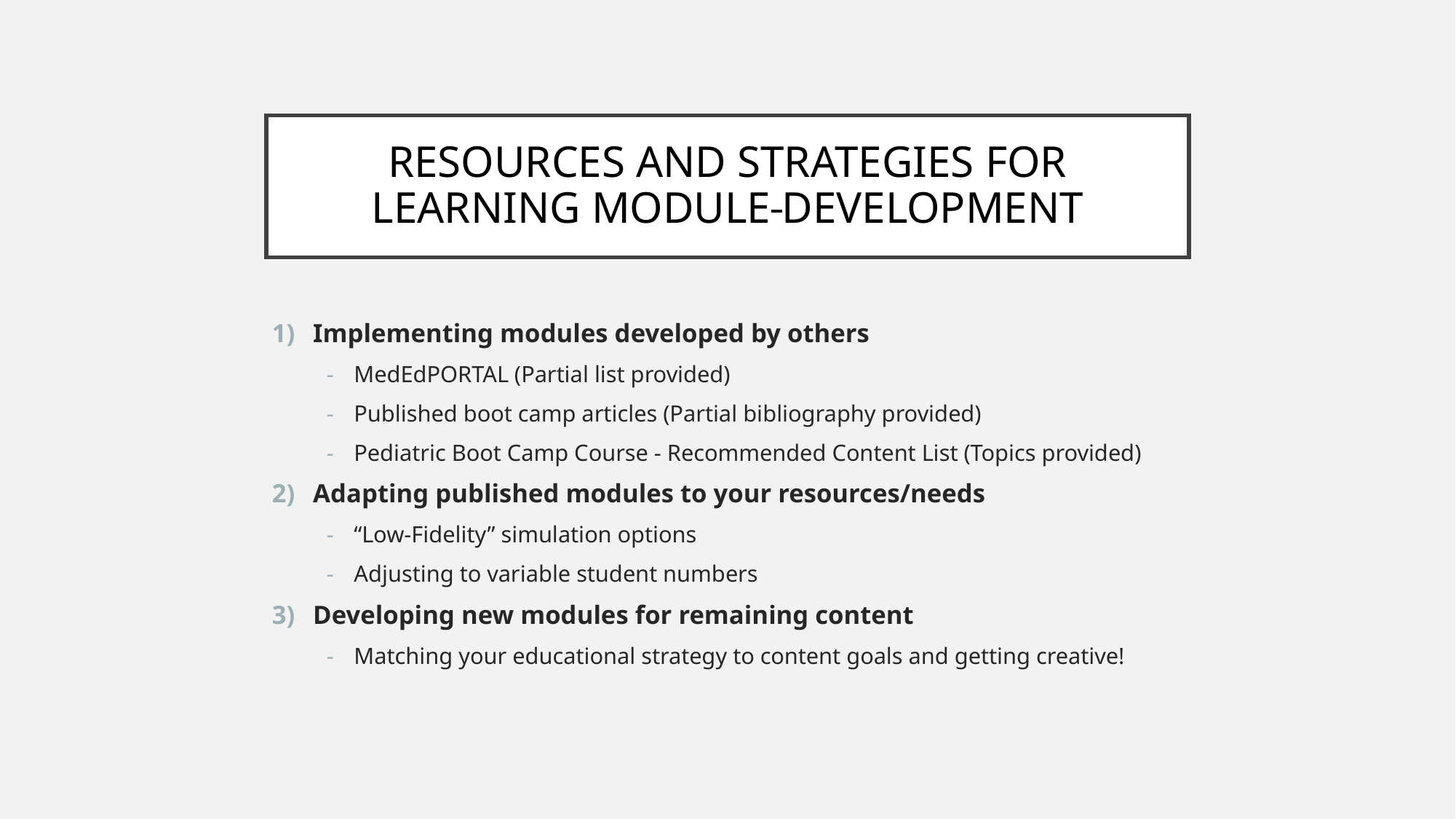

# RESOURCES AND STRATEGIES FOR LEARNING MODULE DEVELOPMENT
Implementing modules developed by others
MedEdPORTAL (Partial list provided)
Published boot camp articles (Partial bibliography provided)
Pediatric Boot Camp Course - Recommended Content List (Topics provided)
Adapting published modules to your resources/needs
“Low-Fidelity” simulation options
Adjusting to variable student numbers
Developing new modules for remaining content
Matching your educational strategy to content goals and getting creative!

## Slide 26
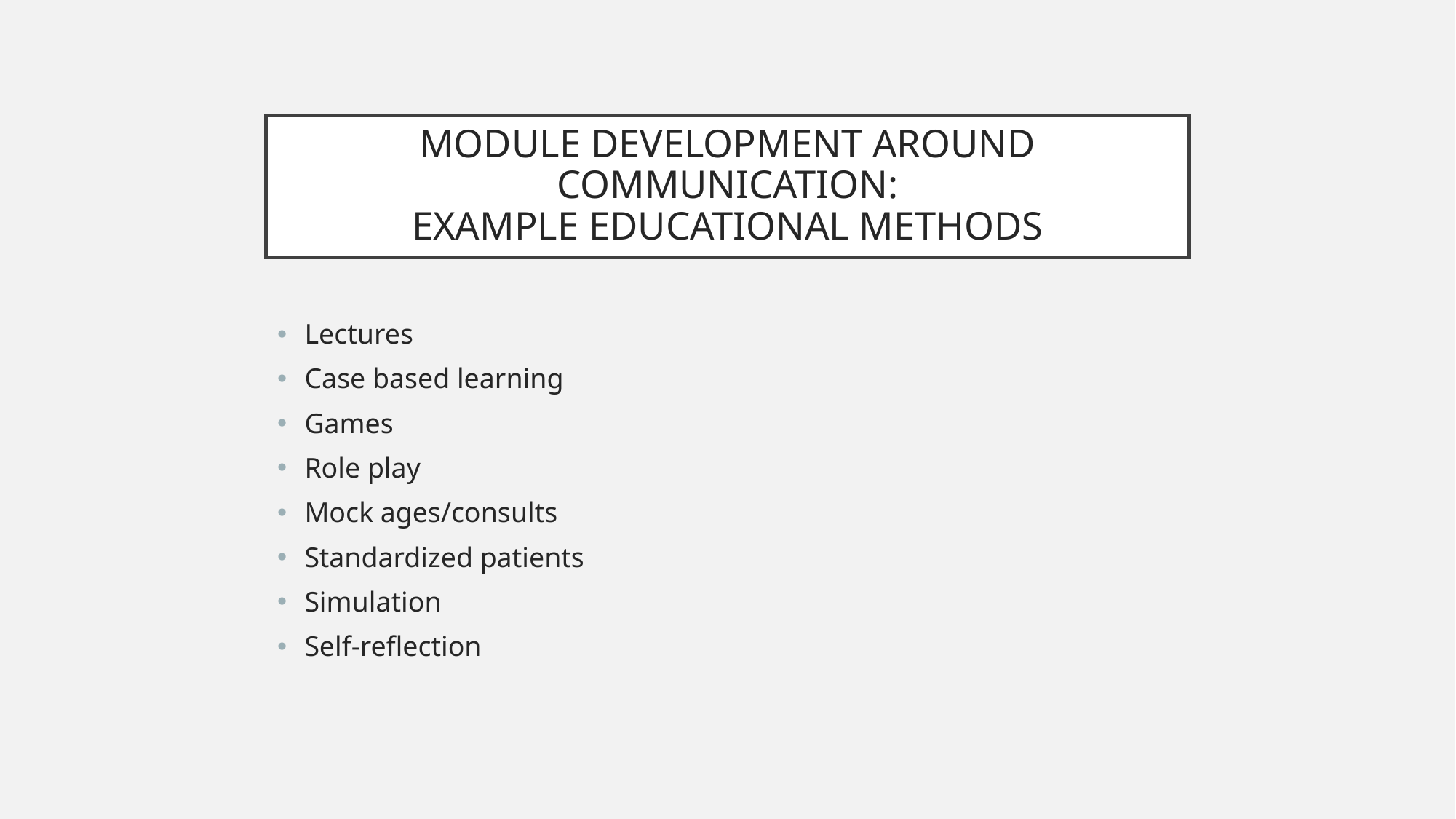

# MODULE DEVELOPMENT AROUND COMMUNICATION: EXAMPLE EDUCATIONAL METHODS
Lectures
Case based learning
Games
Role play
Mock ages/consults
Standardized patients
Simulation
Self-reflection

## Slide 27
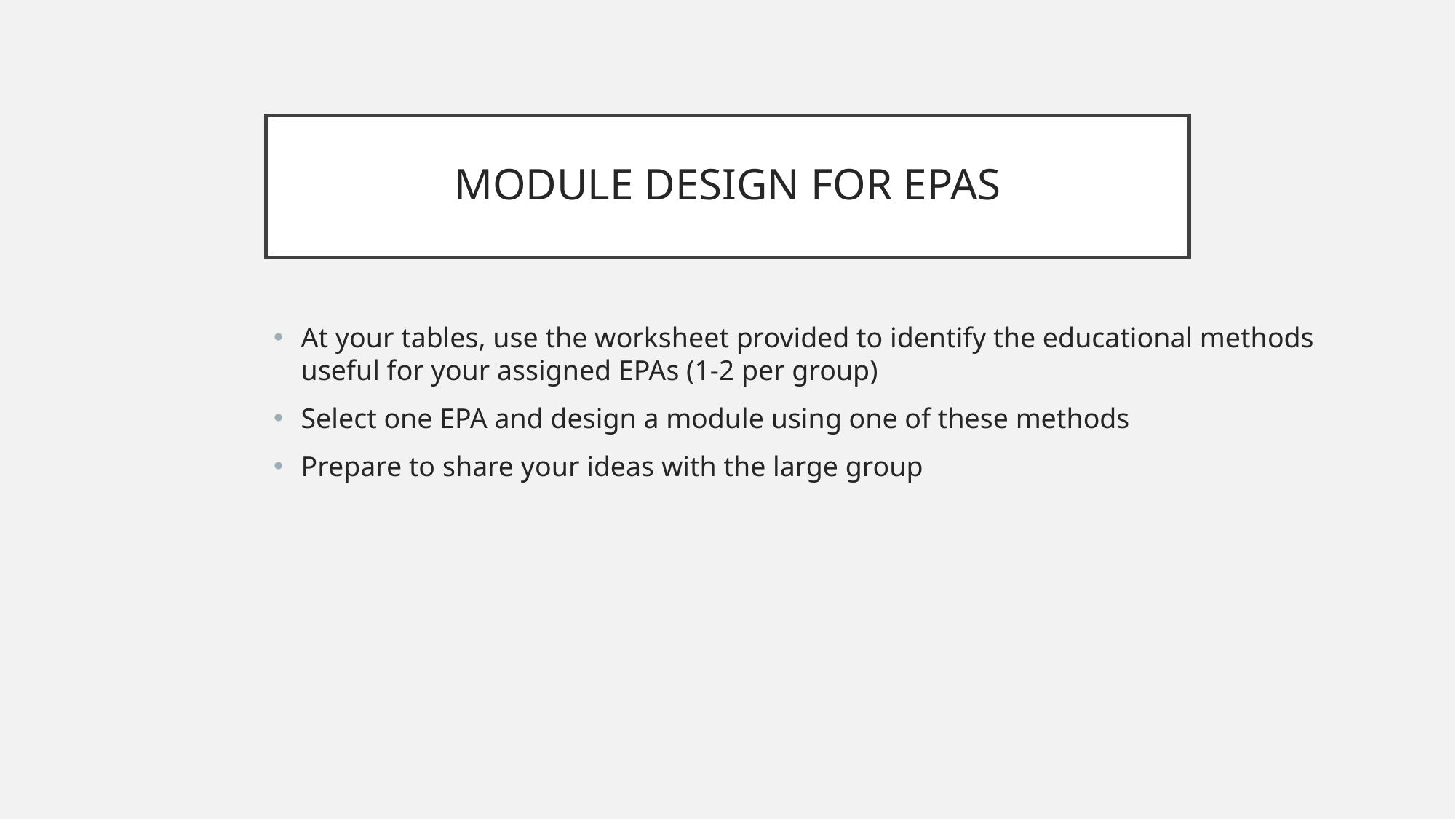

# MODULE DESIGN FOR EPAS
At your tables, use the worksheet provided to identify the educational methods useful for your assigned EPAs (1-2 per group)
Select one EPA and design a module using one of these methods
Prepare to share your ideas with the large group

## Slide 28
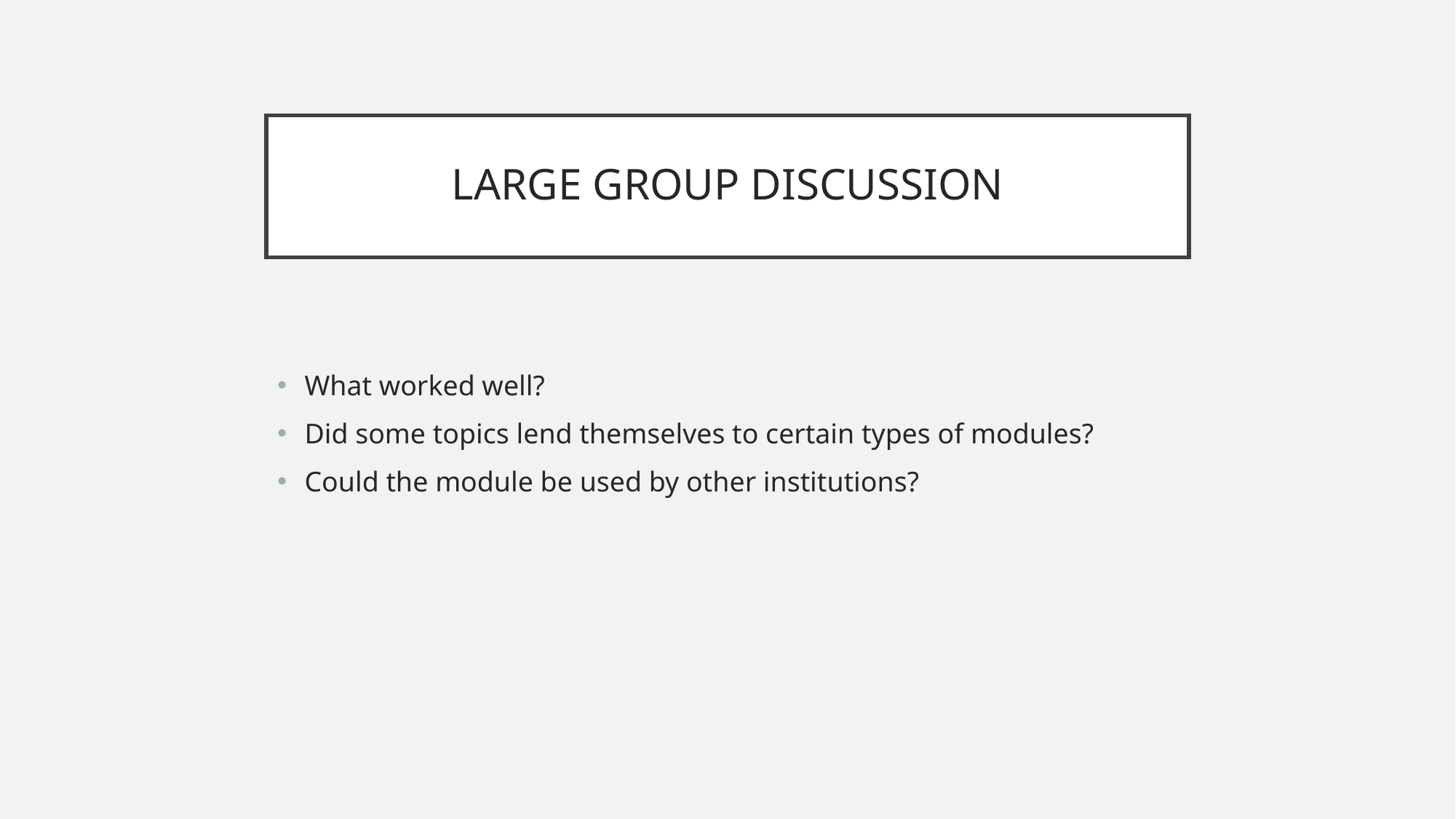

# LARGE GROUP DISCUSSION
What worked well?
Did some topics lend themselves to certain types of modules?
Could the module be used by other institutions?

## Slide 29
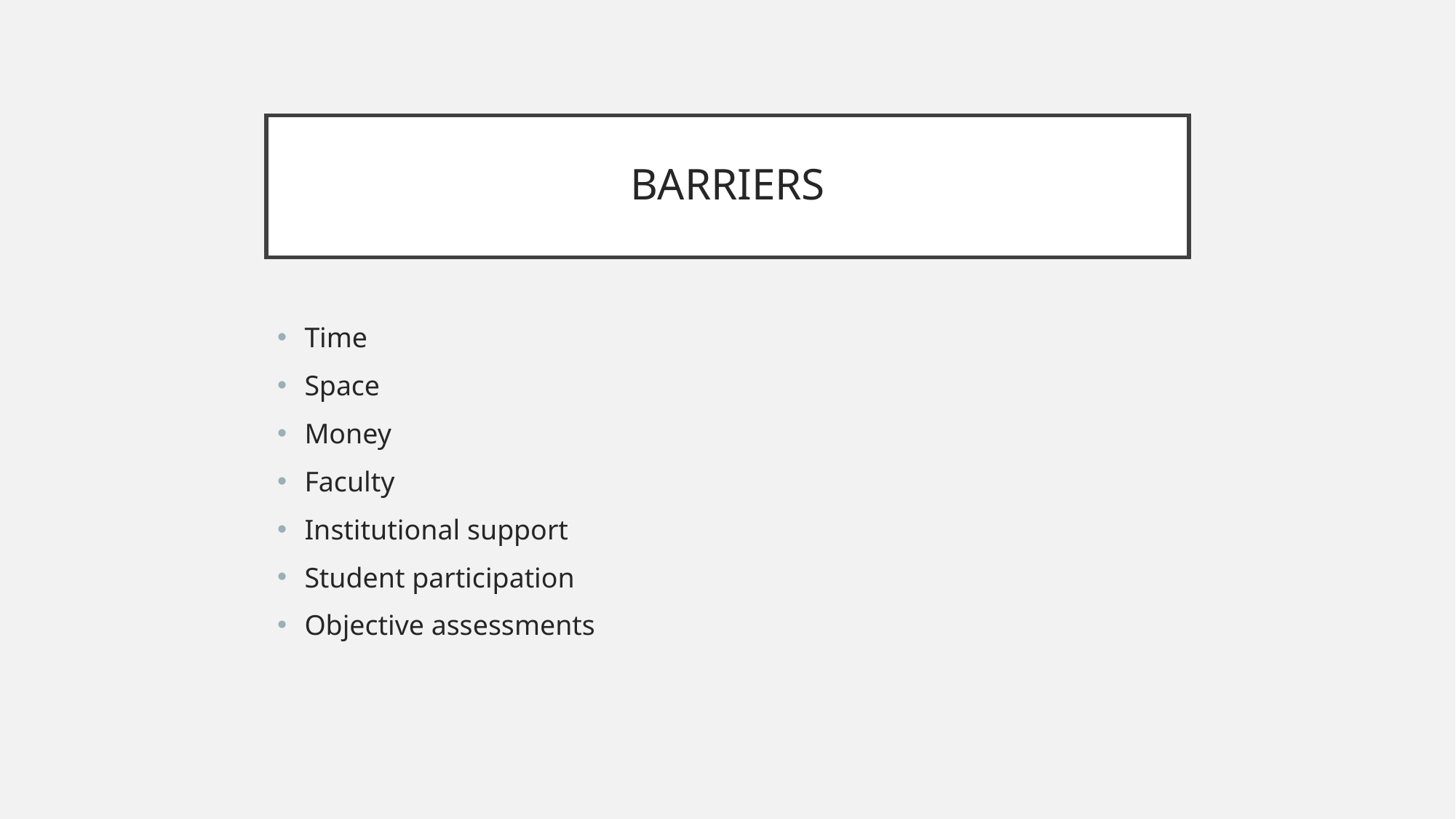

# BARRIERS
Time
Space
Money
Faculty
Institutional support
Student participation
Objective assessments

## Slide 30
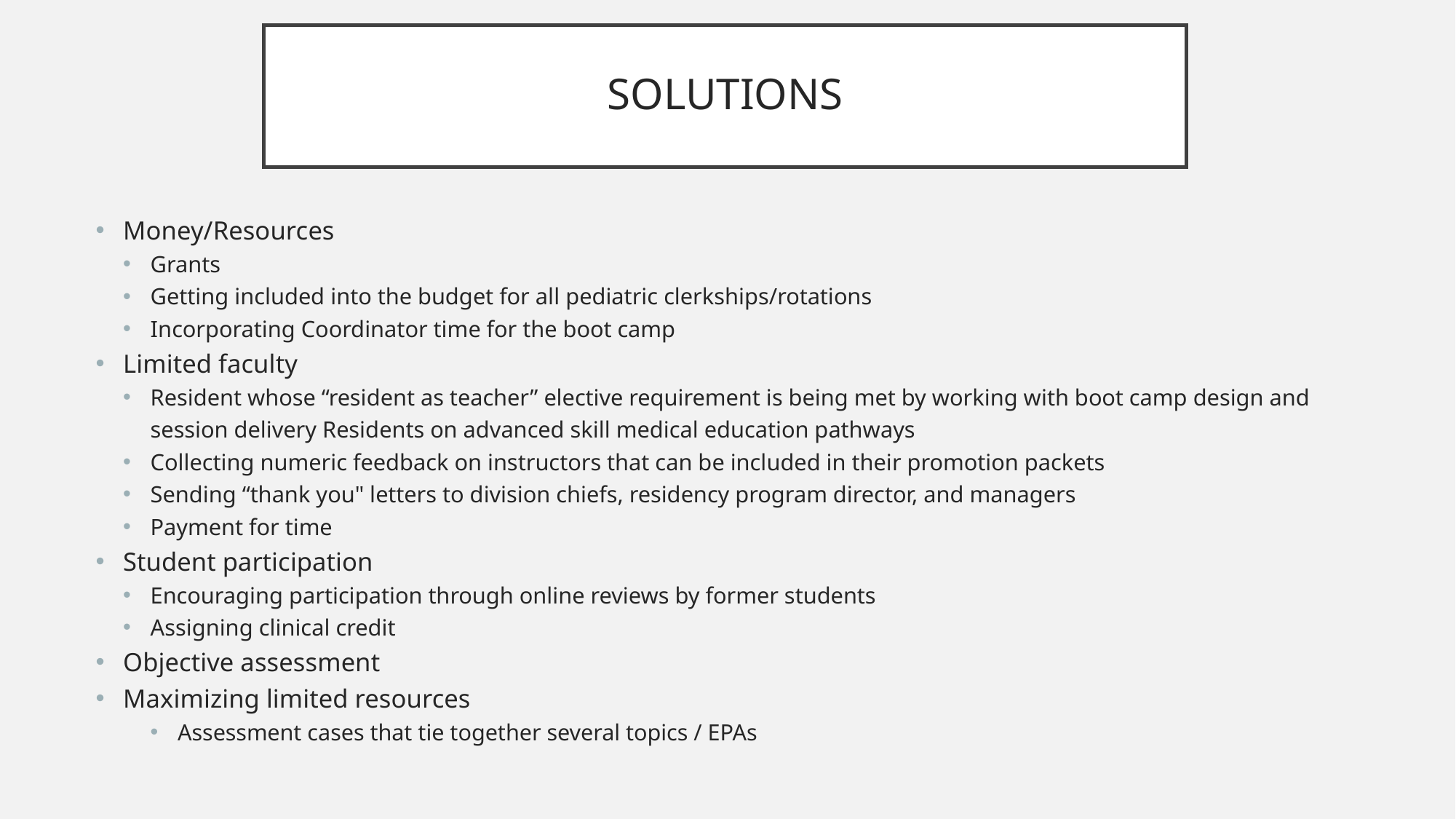

# SOLUTIONS
Money/Resources
Grants
Getting included into the budget for all pediatric clerkships/rotations
Incorporating Coordinator time for the boot camp
Limited faculty
Resident whose “resident as teacher” elective requirement is being met by working with boot camp design and session delivery Residents on advanced skill medical education pathways
Collecting numeric feedback on instructors that can be included in their promotion packets
Sending “thank you" letters to division chiefs, residency program director, and managers
Payment for time
Student participation
Encouraging participation through online reviews by former students
Assigning clinical credit
Objective assessment
Maximizing limited resources
Assessment cases that tie together several topics / EPAs

## Slide 31
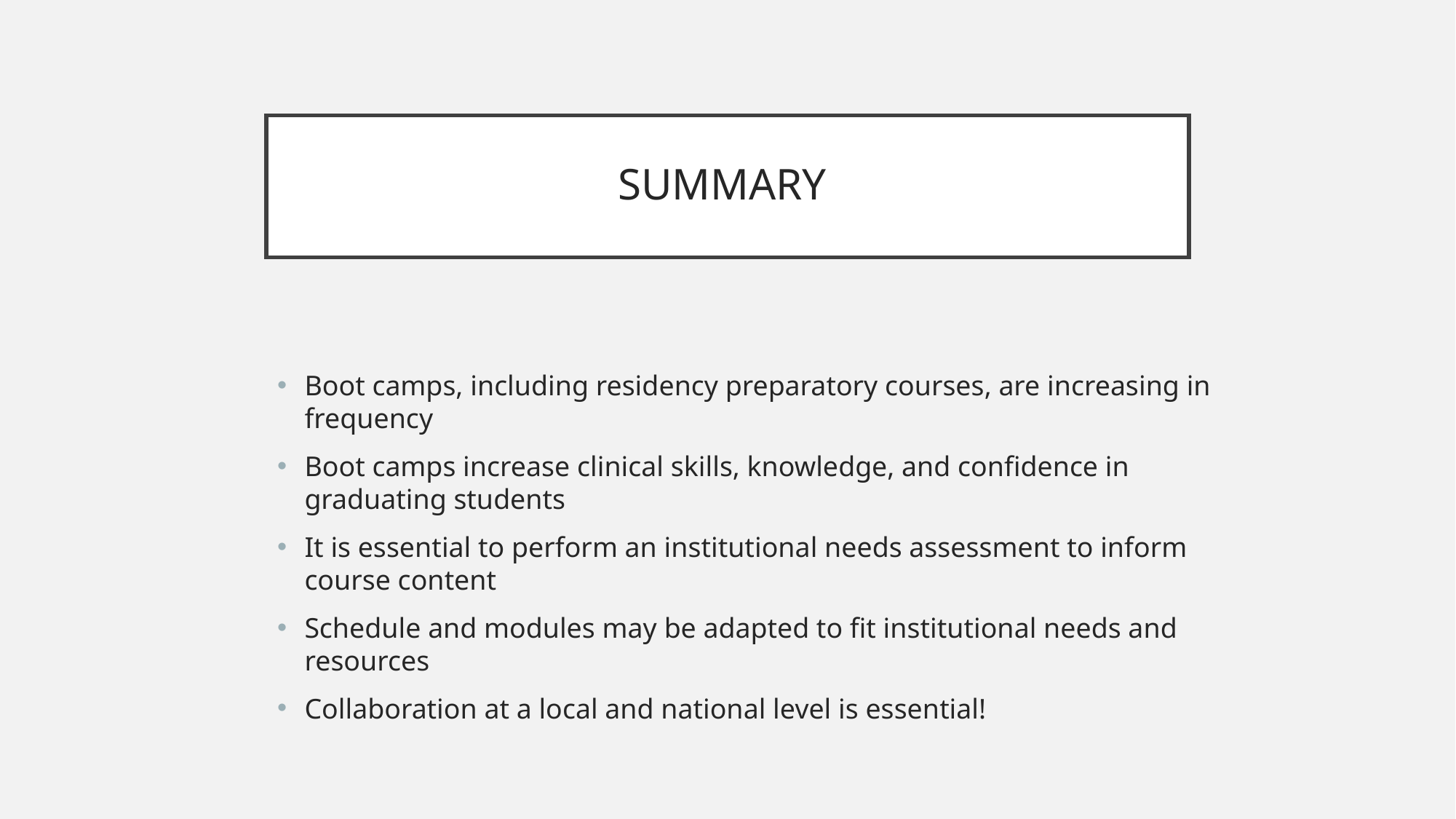

# SUMMARY
Boot camps, including residency preparatory courses, are increasing in frequency
Boot camps increase clinical skills, knowledge, and confidence in graduating students
It is essential to perform an institutional needs assessment to inform course content
Schedule and modules may be adapted to fit institutional needs and resources
Collaboration at a local and national level is essential!
