# Supplementary material for: Building a Boot Camp: Pediatric Residency Preparatory Course Design Workshop and Tool Kit
Source: MedEdPORTAL. 2019 Dec 13;15:10860. doi: 10.15766/mep_2374-8265.10860 (PMC7010200; doi:10.15766/mep_2374-8265.10860)
Supplement: Supplementary file 1 — A. Boot Camp Workshop Presentation.pptx B. Review of Existing Boot Camp Literature.docx C. Institutional Needs Assessment Worksheet.docx D. Recommended Content List and Session Prioritization Worksheet.docx E. Schedule Worksheet and Sample Schedules.docx F. Module Design Worksheet and Planning Resources.docx G. Selected MedEdPORTAL Boot Camp Resources.docx H. Workshop Feedback Surveys.docx I. Facilitator Guide.docx [file mep-15-10860-s001.zip › B. Review of Existing Boot Camp Literature.docx]

Among the many difficult transitions in medical training, that from medical student to intern is one of the most challenging, likely related to an increase in responsibility and independence.^1,2^ In a survey of program directors, common perceived struggles of interns were lack of self-reflection and improvement, poor organizational skills, underdeveloped professionalism and weak medical knowledge.^3^ Additionally, program directors expressed that the Accreditation Council for Graduate Medical Education (ACGME) competencies of patient care, problem-based learning and improvement, interpersonal and communication skills, and professionalism were fundamental to gain prior to residency.^3^ Despite this gap between expectations and performance of interns, the fourth year of medical school is not designed to build skill or autonomy in a students’ chosen field or remediate deficient competencies. The fourth-year curriculum varies widely across medical schools, has been noted to lack clarity, organization and quality control,^4^ and usually provides an opportunity for clinical and scholarly electives driven more by students’ interests than by graduation requirements or preparation for internship.^4,5^ This forms the foundation for the debate about the fourth year of medical school; should it be student-driven or faculty-driven, should it focus on preparation for residency or on enrichment activities, and should there be a fourth year at all?^6^ All this being said, overnight, students-turned-interns are expected to “hit the ground running” with baseline cognitive, psychomotor, and affective tools to effectively and efficiently care for patients.^7^

Rooted in the variability of the fourth year of medical school and the starting expectations of interns, boot camps, also referred to as capstone courses or intern preparatory courses, have gained interest in medical education as a way to further prepare students for the transition from medical school to internship.^1^ A meta-analysis on the effectiveness of boot camps for transitions into residency concluded that boot camp completion was associated with improved clinical skills, knowledge acquisition, and perceived confidence.^1^ Surgical boot camps are the earliest and most heavily reported on in the literature, and the field of general surgery has developed a national residency preparatory curriculum to be delivered prior to the beginning of surgical internship.^8^ Many other fields have introduced intern preparatory courses during medical school^9,10^ and there are also boot camp courses to prepare residents for fellowships.^11,12^ Specifically related to pediatrics, Burns et al, reported on a five-day pediatric elective boot camp for students entering pediatrics, internal medicine-pediatrics, and family medicine residences. They found that the course was well-received and led to improved performance of assessed targeted skills and increased self-reported preparedness for many targeted domains.^13^ Further contributing to the literature, a program evaluation based on a conceptual framework discussed the learner appreciation of “hands on” and interactive activities and how a framework of self-efficacy could be a useful lens to develop and evaluate a boot camp.^14^ An increasing number of residency programs are also beginning to introduce *in vivo* boot camps at the start of internship.^15^

As educators in undergraduate medical education, the authors’ goal has been to improve graduating medical students’ transition to residency. The authors hope that this background knowledge will be a valuable resource to educators in pediatrics as well as other disciplines as they embark on a boot camp course design workshop, independently use the toolkit to create their own boot camp, or revise and refine existing boot camp courses.

Resources

1. Blackmore C, Austin J, Lopushinsky SR, Donnon T. Effects of postgraduate medical education "boot camps" on clinical skills, knowledge, and confidence: A meta-analysis. J Grad Med Educ. 2014; 6:643–52.
2. Teo AR, Harleman E, O'Sullivan PS, Maa J. The key role of a transition course in preparing medical students for internship. Acad Med. 2011; 86:860–65.
3. Lyss-Lerman P, Teherani A, Aagaard E, Loeser H, Cooke M, Harper GM. What training is needed in the fourth year of medical school? Views of residency program directors. Acad Med. 2009; 84:823–29.
4. Walling A, Merando A: The fourth year of medical education. A literature review. Acad Med. 2010; 85:1698–1704.
5. Goldfarb S, Morrison G. The 3-year medical school--change or shortchange? N Engl J Med. 2013; 369(12): 1087-9.
6. Kanter SL. How to win an argument about the senior year of medical school. Acad Med. 2009. 84(7): 815-816.
7. Reddy ST, Chao J, Carter JL, Drucker R, Katz NT, Nesbit R, et al. Alliance for clinical education perspective paper: Recommendations for redesigning the “final year” of medical school. Teach Learn Med. 2014; 26(4): 420-427.
8. American College of Surgeons (ACS)/Association of Program Directors in Surgery (APDS)/Association for Surgical Education (ASE) Resident Prep Curriculum: <https://www.facs.org/education/program/resident-prep>.
9. Lamba S, Wilson B, Natal B, Nagurka R, Anana M, Sule H. A suggested emergency medicine boot camp curriculum for medical students based on the mapping of Core Entrustable Professional Activities to Emergency Medicine Level 1 milestones. Adv Med Educ Pract. 2016; 7: 115-124.
10. Lerner, V, Higgins EE, Winkel A. Re-boot: Simulation Elective for Medical Students as Preparation Boot Camp for Obstetrics and Gynecology Residency. Cureus. 2018. 10(6): e2811
11. Maskatia SA, Cabrera AG, Morris SA, Altman CA. The pediatric echocardiography Boot Camp: Four-year experience and impact on clinical performance. Echocardiography. 2017; 34(10): 1486-1494.
12. Nishisaki A, Hales R, Biagas K, Cheifetz I, Corriveau C, Garber N, et al. A multi-institutional high-fidelity simulation “boot camp” orientation and training program for first year pediatric critical care fellows. Pediatr Crit Care Med. 2009; 10(2): 157-162.
13. Burns R, Adler M, Mangold K, Trainor J. Brief Boot Camp for 4th-Year Medical Students Entering into Pediatric and Family Medicine Residencies. Cureus. 2016; 8(2): e488.
14. Pete Devon, E; Tenney-Soeiro, R; Ronan J; Balmer D. A Pediatric Preintern Boot Camp: Program Development and Evaluation Informed by a Conceptual Framework. Acad Ped; 2018.
15. Winn, AS; Marcus CH; Williams K; Smith GC; Gorbounova I; Sectish TC; Landrigan CP. Development, Implementation, and Assessment of the Intensive Clinical Orientation for Residents (ICOR) Curriculum: A Pilot Intervention to Improve Intern Clinical Preparedness. Academic Pediatrics. 2018; 18: 140-144.
16. Pete Devon E, Ronan J, Tenney-Soeiro R, Balmer D. Current state of the intern preparatory course: findings from a national survey of pediatric clerkship directors. Journal of Family Medicine & Community Health. 2017; 4(6): 1124-1128.
