# Supplementary material for: Building a Boot Camp: Pediatric Residency Preparatory Course Design Workshop and Tool Kit
Source: MedEdPORTAL. 2019 Dec 13;15:10860. doi: 10.15766/mep_2374-8265.10860 (PMC7010200; doi:10.15766/mep_2374-8265.10860)
Supplement: Supplementary file 1 — A. Boot Camp Workshop Presentation.pptx B. Review of Existing Boot Camp Literature.docx C. Institutional Needs Assessment Worksheet.docx D. Recommended Content List and Session Prioritization Worksheet.docx E. Schedule Worksheet and Sample Schedules.docx F. Module Design Worksheet and Planning Resources.docx G. Selected MedEdPORTAL Boot Camp Resources.docx H. Workshop Feedback Surveys.docx I. Facilitator Guide.docx [file mep-15-10860-s001.zip › C. Institutional Needs Assessment Worksheet.docx]

**Needs Assessment Worksheet - Part 1**

∙If you have conducted a needs assessment, what was done? ___________________________________

_____________________________________________________________________________________

_____________________________________________________________________________________

∙What gaps/needs were identified? ________________________________________________________

_____________________________________________________________________________________

_____________________________________________________________________________________

∙How were they identified? ______________________________________________________________

_____________________________________________________________________________________

_____________________________________________________________________________________

∙Which stakeholders can contribute to your needs assessment? How can you involve them?*

Local clerkship directors: ________________________________________________________________

_____________________________________________________________________________________

Program directors where your students often match: _________________________________________

_____________________________________________________________________________________

Current interns at your own program: ­­­_____________________________________________________

_____________________________________________________________________________________

Current graduating students: ____________________________________________________________

_____________________________________________________________________________________

Former students: ______________________________________________________________________

_____________________________________________________________________________________

Other: _______________________________________________________________________________

*Surveys, focus groups, individual interviews, previously collected data, etc.

**Needs Assessment Worksheet - Part 2**

∙Total days available: ___________________________________________________________________

∙Pediatric specific boot camp or general boot camp? __________________________________________

If general, how much time allotted to Pediatrics topics? _________________________________

∙Required or elective course? _____________________________________________________________

∙Number of students that could be or need to be accommodated: _______________________________

∙Number of faculty needed: _____________________________________________________________

∙Number of faculty available: ­­­­­­_____________________________________________________________

∙Facility resources available: ______________________________________________________________

|  | **Where is the EPA Addressed in your curriculum?** | | | | |
| --- | --- | --- | --- | --- | --- |
| **Entrustable Professional Activities**  **For Entering Residency** | **Preclinical Curriculum** | **Clerkship** | **Sub-I / Senior Course** | **Other** | **Not Addressed/ Needs Coverage in Boot Camp** |
| EPA 1: Gather a history and perform a physical examination |  |  |  |  |  |
| EPA 2: Prioritize a differential diagnosis following a clinical encounter |  |  |  |  |  |
| EPA 3: Recommend and interpret common diagnostic and screening tests |  |  |  |  |  |
| EPA 4: Enter and discuss orders and prescriptions |  |  |  |  |  |
| EPA 5: Document a clinical encounter in the patient record |  |  |  |  |  |
| EPA 6: Provide an oral presentation of a clinical encounter |  |  |  |  |  |
| EPA 7: Form clinical questions and retrieve evidence to advance patient care |  |  |  |  |  |
| EPA 8: Give or receive a patient handover to transition care responsibly |  |  |  |  |  |
| EPA 9: Collaborate as a member of an interprofessional team |  |  |  |  |  |
| EPA 10: Recognize a patient requiring urgent or emergent care and initiate evaluation and management |  |  |  |  |  |
| EPA 11: Obtain informed consent for tests and/or procedures |  |  |  |  |  |
| EPA 12: Perform general procedures of a physician |  |  |  |  |  |
| EPA 13: Identify system failures and contribute to a culture of safety and improvement |  |  |  |  |  |

**Example Needs Assessment Worksheet - Part 1**

∙If you have conducted a needs assessment, what was done? *At our institution, we sent a survey to current first and second year residents with questions related to what knowledge and skills they wish they had for entering residency. We also discussed perceived knowledge, skills, and attitudes deficiencies with our residency program director. Also, since the boot camp creators are program directors of undergraduate medical education curricula, we evaluated gaps in our curriculum.*

∙What gaps/needs were identified? *We identified a wide variety of perceived needs from electrolyte management and review of asthma and bronchiolitis to interprofessional communication and time management skills to lumbar punctures, performing EKGs and phlebotomy skills.*

∙How were they identified? *Survey Monkey, curriculum review, discussions with leadership*

∙Which stakeholders can contribute to your needs assessment? How can you involve them?*

Local clerkship directors: *Our local clerkship directors helped to create the boot camp curriculum, so they were highly invested.*

Program directors where your students often match: *We only spoke to the residency program director at our own institution where we usually have multiple students match.*

Current interns at your own program: ­­ *We did take this approach via a survey. This didn’t necessarily tell us gaps in our own curriculum but did let us know what current interns and residents were experiencing and need to know.*

Current graduating students: *We did not survey graduating students, but I think small focus groups would be an effective way to gather high yield information from an invested group and would round out the perspectives*.

Former students: *We didn’t reach out to our previously graduated students (now interns), but this would have been particularly helpful. While we didn’t specifically survey all recently graduated students, we did capture a number of them when we surveyed our current interns and residents.*

Other: _______________________________________________________________________________

**The authors have found that individual emails or emailed survey links work well for soliciting input from former students, local clerkship directors and residency program directors. In addition to emails/electronic surveys, focus groups and post-boot camp evaluation surveys (for existing boot camps) provide additional stakeholder input. Other sources include previously gathered data including institutional surveys of prior graduates or their program directors that comment on areas of student readiness to enter internship.*

**Example Needs Assessment Worksheet - Part 2**

∙Total days available: *5*-*we thought about this from both our time, students’ time, finances, and space availability.*

∙Pediatric specific boot camp or General boot camp? *Pediatric specific*

If general, how much time allotted to Pediatrics topics? *N/A*

∙Required or elective course? *Elective*

∙Number of students that could be or need to be accommodated: *We decided this based on rate limiting step, which was our simulation space. 12 students*

∙Number of faculty needed: *15 if no one did more than 1 day.* *Resource intense. In addition to faculty, consider other staff that are needed; i.e., simulation operators or respiratory therapists. Also consider, who else can help such as residents and fellows.*

∙Number of faculty available: *N/A*

∙Facility resources available: *Simulation space for 3 hours on 2 days. Classrooms or meeting rooms large enough to accommodate 3 small groups of 4 for the remaining sessions. Need to book far in advance for optimal space availability. For particular spaces that are needed, plan around that availability; for us, it was the simulation space.*

**Example Needs Assessment Worksheet - Part 2**

|  | **Where is the EPA Addressed in your curriculum?** | | | | |
| --- | --- | --- | --- | --- | --- |
| **EPA** | **Preclinical Curriculum** | **Clerkship** | **Sub-I / Senior Course** | **Other** | **Not Addressed/ Needs Coverage in Boot Camp** |
| EPA 1: Gather a history and perform a physical examination | X | X | X |  |  |
| EPA 2: Prioritize a differential diagnosis following a clinical encounter | X | X | X |  |  |
| EPA 3: Recommend and interpret common diagnostic and screening tests | X | X | X |  | X |
| EPA 4: Enter and discuss orders and prescriptions |  |  | X |  |  |
| EPA 5: Document a clinical encounter in the patient record |  | X | X |  | X |
| EPA 6: Provide an oral presentation of a clinical encounter |  | X | X |  |  |
| EPA 7: Form clinical questions and retrieve evidence to advance patient care |  | X | X |  |  |
| EPA 8: Give or receive a patient handover to transition care responsibly |  |  | X |  | X |
| EPA 9: Collaborate as a member of an interprofessional team |  | X | X |  | X |
| EPA 10: Recognize a patient requiring urgent or emergent care and initiate evaluation and management |  | X | X |  | X |
| EPA 11: Obtain informed consent for tests and/or procedures |  |  |  |  | X |
| EPA 12: Perform general procedures of a physician |  |  |  |  | X |
| EPA 13: Identify system failures and contribute to a culture of safety and improvement |  | X |  |  | X |
