# Supplementary material for: Building a Boot Camp: Pediatric Residency Preparatory Course Design Workshop and Tool Kit
Source: MedEdPORTAL. 2019 Dec 13;15:10860. doi: 10.15766/mep_2374-8265.10860 (PMC7010200; doi:10.15766/mep_2374-8265.10860)
Supplement: Supplementary file 1 — A. Boot Camp Workshop Presentation.pptx B. Review of Existing Boot Camp Literature.docx C. Institutional Needs Assessment Worksheet.docx D. Recommended Content List and Session Prioritization Worksheet.docx E. Schedule Worksheet and Sample Schedules.docx F. Module Design Worksheet and Planning Resources.docx G. Selected MedEdPORTAL Boot Camp Resources.docx H. Workshop Feedback Surveys.docx I. Facilitator Guide.docx [file mep-15-10860-s001.zip › D. Recommended Content List and Session Prioritization Worksheet.docx]

**Recommended Content:**

**1) Patient Care / Medical Knowledge and Skills**

Knowledge

- Interpreting diagnostics - EKG, ABG/VBG, CXR, CSF, AXR
- Pain management
- Lines (PIV, CVL, UVC, UAC, etc) and Tubes (ETT, enteral, etc)
- Writing a prescription
- Parenting / Mommy Call topics
- Respiratory care
- Basic Fluids and Nutrition
- Bugs and Drugs
- Emergency management / Initial Assessment
- Common outpatient/ED/inpatient cases

Skills

- Bag mask ventilation and Supplemental Oxygen Delivery Systems
- Lumbar puncture
- Venipuncture, Arterial puncture, IV/IO access
- Suturing
- Urinary catheterization
- Nasogastric Tube placement

**2) Professionalism**

- Wellness
- Organizational tools

**3) Interpersonal and Communication Skills**

- Handoffs
- Calling a consult
- Interprofessional communication
- Discussing difficult news
- Informed consent
- Documentation
- Answering pages
- Resident as Teacher

**4) Practice Based Learning**

- Lifelong learning
- Feedback (Giving and Receiving)
- Landmark studies in pediatrics/AAP policies/EBM/Resources

**5) System Based Learning**

- High value care
- Discharge planning

**Session Prioritization Worksheet**

Use results of the Needs Assessment, the Recommended Content List, and the Sample Schedules provided to prioritize what to include in your sample boot camp schedule. You may consider categorizing content as Knowledge, Procedures and Attitudes.

| **Absolutely necessary** | **Maybe** | **No time for this ☹** |
| --- | --- | --- |
|  |  |  |

**Session Prioritization Worksheet Example**

Use results of the Needs Assessment, the Recommended Content List, and the Sample Schedules provided to prioritize what to include in your sample boot camp schedule. You may consider categorizing content as Knowledge, Procedures and Attitudes.

| **Absolutely necessary** | **Maybe** | **No time for this ☹** |
| --- | --- | --- |
| **Knowledge**  Reading CXRs  Lines and tubes  **Procedures**  Phlebotomy  Lumbar punctures  Performing EKGs  Informed consent  **Attitudes**  Giving and receiving feedback  Difficult communication techniques  Interprofessional communication | **Knowledge**  **Procedures**  Placing a peripheral intravenous line  Placing orders  **Attitudes** | **Knowledge**  A lot of medical knowledge  Parent questions  **Procedures**  Calling a consult  **Attitudes**  Evidence based medicine review  Specific physical exam techniques  Medical errors |
