# Supplementary material for: Building a Boot Camp: Pediatric Residency Preparatory Course Design Workshop and Tool Kit
Source: MedEdPORTAL. 2019 Dec 13;15:10860. doi: 10.15766/mep_2374-8265.10860 (PMC7010200; doi:10.15766/mep_2374-8265.10860)
Supplement: Supplementary file 1 — A. Boot Camp Workshop Presentation.pptx B. Review of Existing Boot Camp Literature.docx C. Institutional Needs Assessment Worksheet.docx D. Recommended Content List and Session Prioritization Worksheet.docx E. Schedule Worksheet and Sample Schedules.docx F. Module Design Worksheet and Planning Resources.docx G. Selected MedEdPORTAL Boot Camp Resources.docx H. Workshop Feedback Surveys.docx I. Facilitator Guide.docx [file mep-15-10860-s001.zip › E. Schedule Worksheet and Sample Schedules.docx]

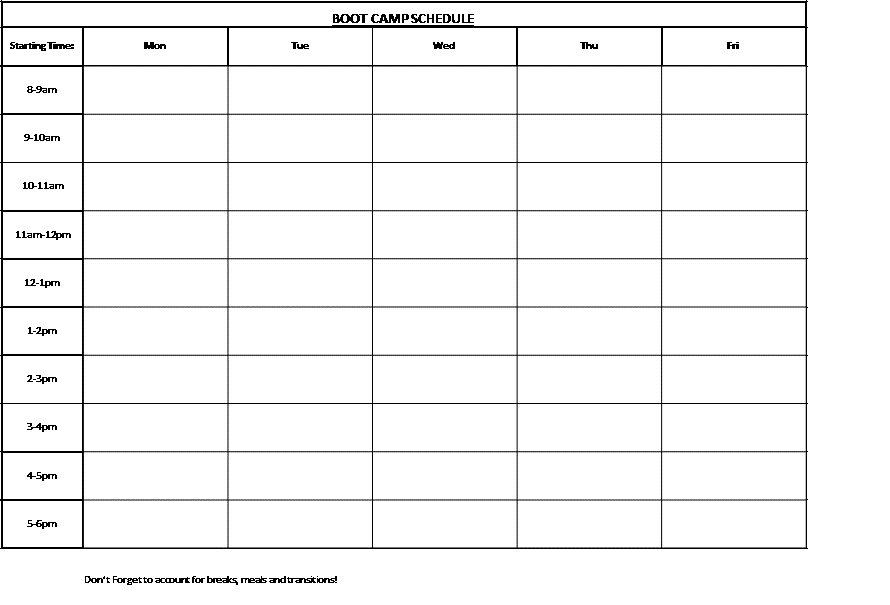
 **Bootcamp Schedule Worksheet**

**
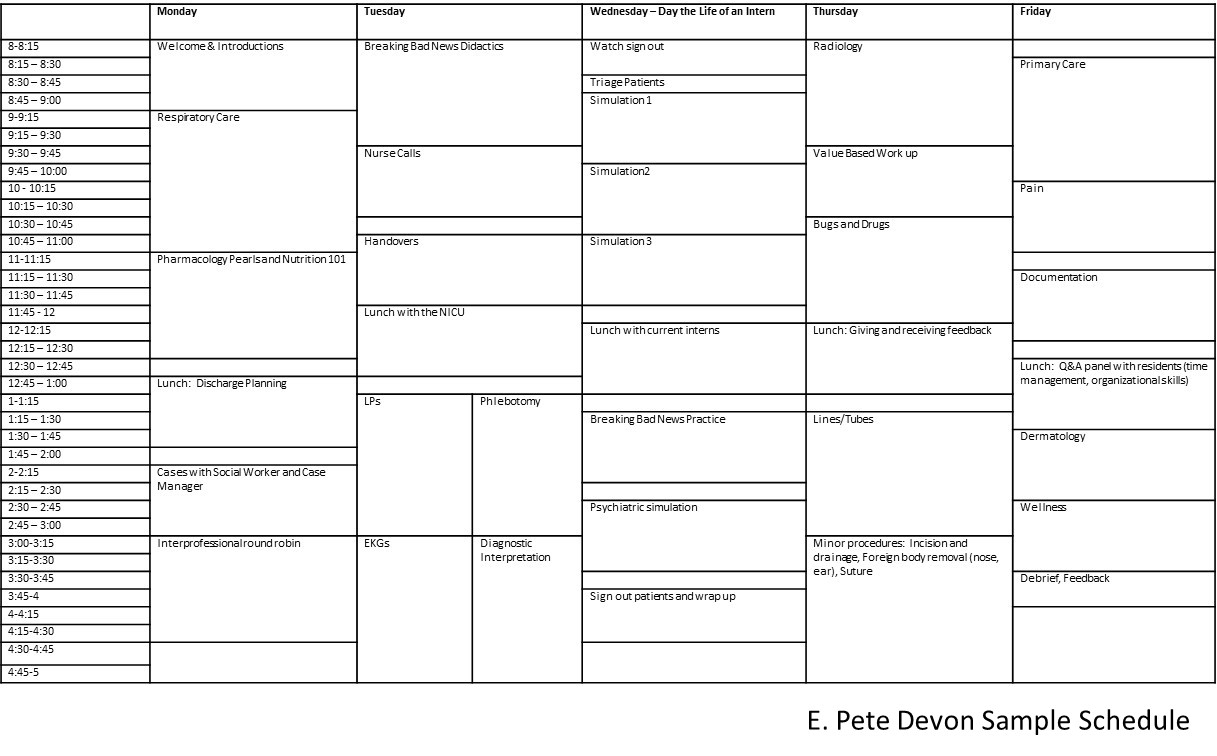
Example Boot Camp Schedule – One Week Duration**

**Example Boot Camp Schedule – Two Week Duration (example #1)**


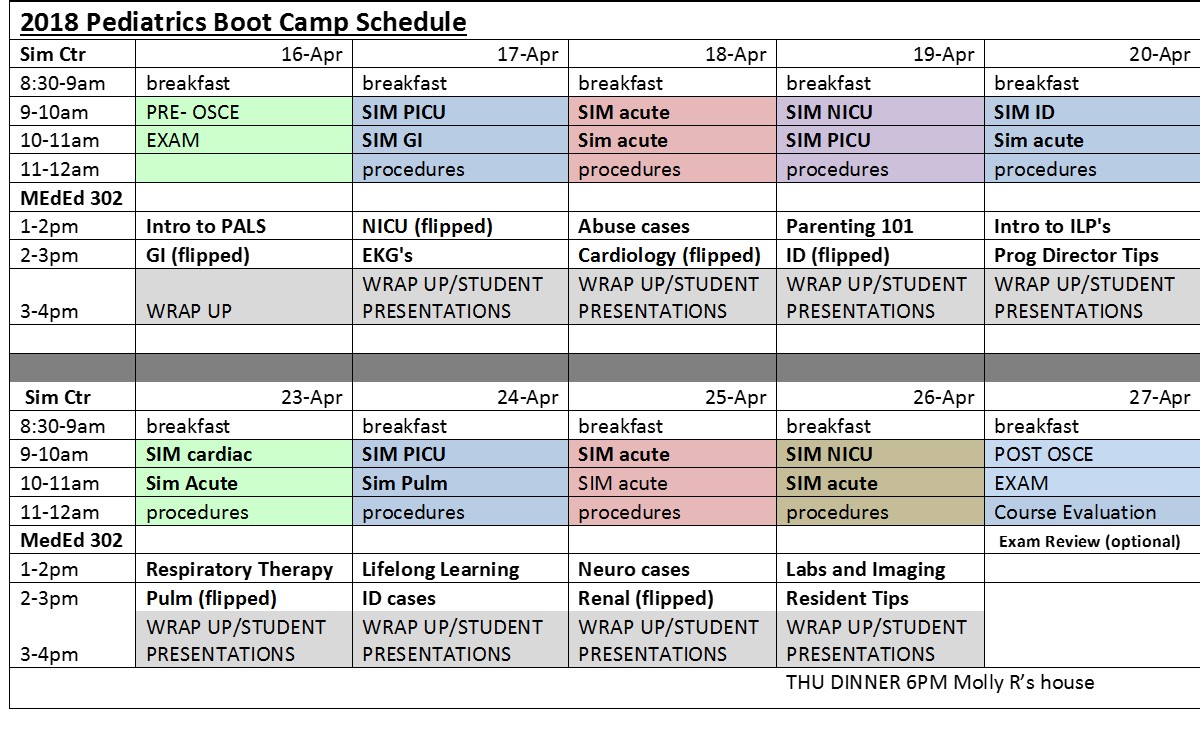


M. Rideout Sample Schedule

*Abbreviations: OSCE-Objective Structured Clinical Examination; PALS: Pediatric Advanced Life Support, PICU: Pediatric ICU, NICU: Neonatal ICU, ILP – Individualized Learning Plan, Flipped – Flipped Classroom model

**
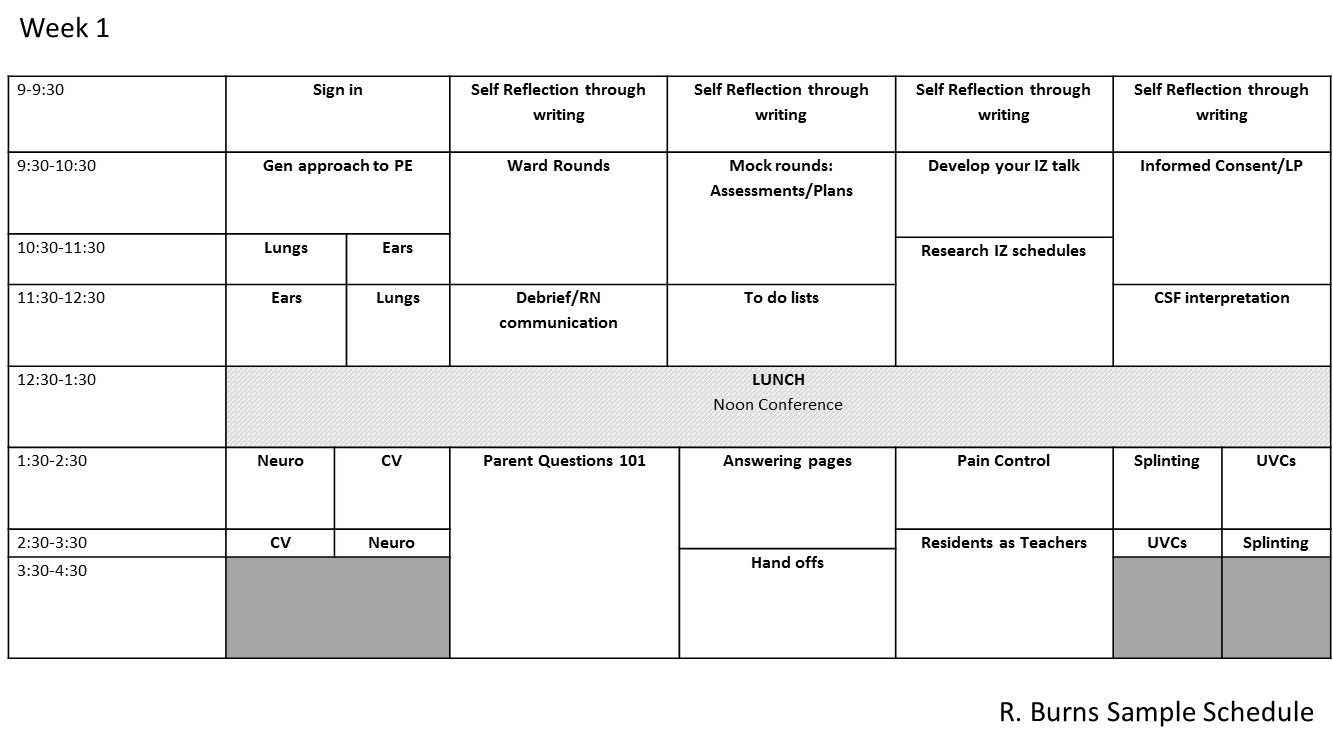
Example Boot Camp Schedule – Two Week Duration (example #2, week 1 of 2)**

*Abbreviations: IZ - Immunizations; UVC – Umbilical Venous Catheter, PE – Physical Exam

**
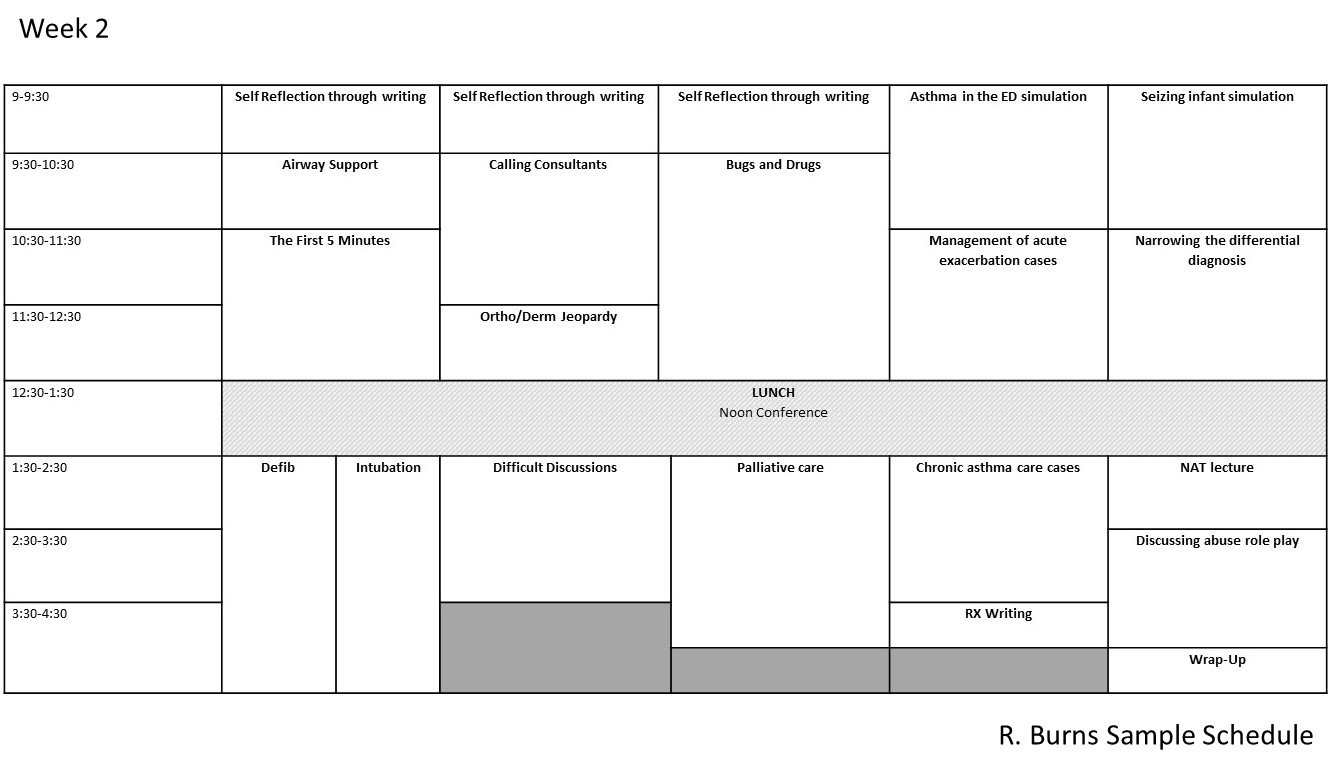
Example Boot Camp Schedule – Two Week Duration (example #2, week 2 of 2)**

*Abbreviations: NAT – Non accidental Trauma

**Example Boot Camp Schedule – Four Week Duration Including 2 Weeks of General and 2 Weeks Pediatric Content**

Topics covered in 2 weeks of general sessions attended by all students prior to specialty specific course weeks:

Specific Knowledge Areas and Simulation Topics:

Radiology: Ultrasound, CT, MRI, Plain films

Radiology-Interventions: Indications and Common Mistakes

Advanced Airway Management

ABG/Vent/Anesthesia/ICU/Respiratory Support

ECG: dysrhythmia and blocks, Ischemia and Infarcts

Advanced Pain Management

Communication Strategies and Contracts

Antibiotics

Transfusion medicine

Oxygen Delivery

Asthma/COPD/Adjuncts

IVF/Electrolytes

Stroke Cases

BLS/ACLS

Generalizable/Milestones Material:

I-PASS Handoff (Illness severity, Patient Summary, Action List, Situation Awareness/Contingency Plan, Synthesis by Receiver)

Capacity/competency

Documentation and Coding: Common Mistakes

Admissions and Discharges; Death Certificates

Medico legal: Mistakes and Communication

Medico legal: The Process and The Deposition

Conflict Management Panel

Resident efficiency/effectiveness

Stress and Fatigue Panel

Residents as Teachers

Other:

Intern panel

Integrative Medicine

Global Health

Mock Trial

Wilderness Medicine

The Physician Executive

Financial Management

A. Hartke Sample Schedule


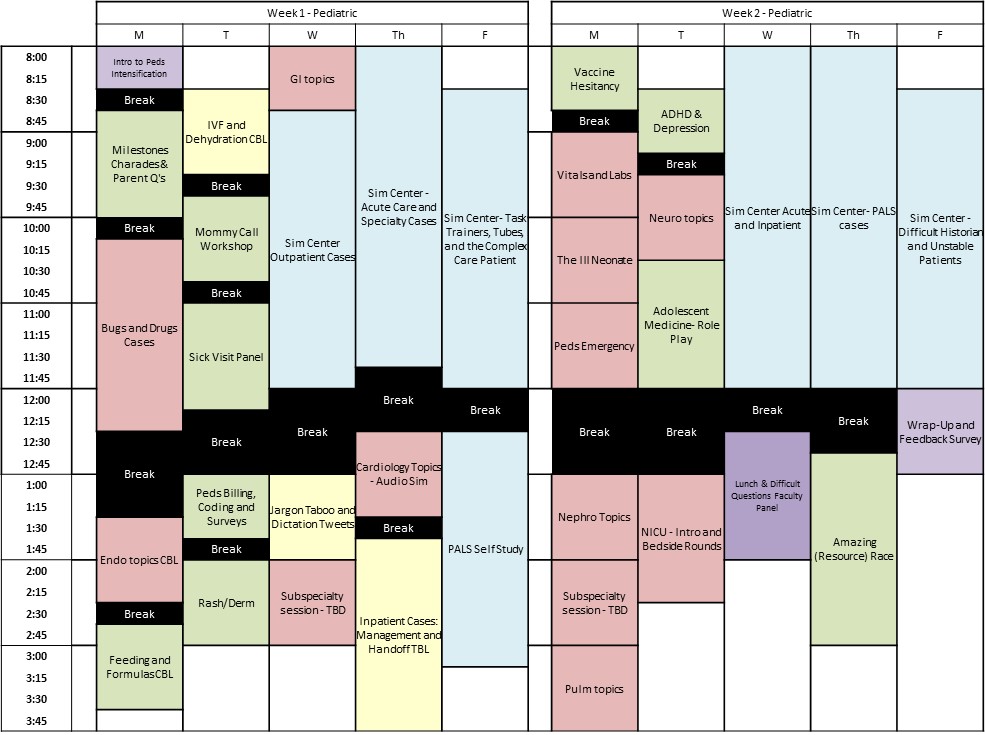
**Example Boot Camp Schedule – Four Week Duration Including 2 Weeks of General and 2 Weeks Pediatric Content**

*Abbreviations: CBL – Case Based Learning, TBL – Team Based Learning

A. Hartke Sample Schedule
