# Supplementary material for: Building a Boot Camp: Pediatric Residency Preparatory Course Design Workshop and Tool Kit
Source: MedEdPORTAL. 2019 Dec 13;15:10860. doi: 10.15766/mep_2374-8265.10860 (PMC7010200; doi:10.15766/mep_2374-8265.10860)
Supplement: Supplementary file 1 — A. Boot Camp Workshop Presentation.pptx B. Review of Existing Boot Camp Literature.docx C. Institutional Needs Assessment Worksheet.docx D. Recommended Content List and Session Prioritization Worksheet.docx E. Schedule Worksheet and Sample Schedules.docx F. Module Design Worksheet and Planning Resources.docx G. Selected MedEdPORTAL Boot Camp Resources.docx H. Workshop Feedback Surveys.docx I. Facilitator Guide.docx [file mep-15-10860-s001.zip › F. Module Design Worksheet and Planning Resources.docx]

**Module Design Worksheet Part 1**

Identify the educational methods most useful/applicable to Entrustable Professional Activity (EPA) you would like to address. On Part 2, select one (or more!) EPA and one method to design a module for a Pediatric Boot Camp.

|  | EPA | Lecture or Reading | Programmed Learning | Discussion | Reflection or Writing Experience | Game, Team or Problem Based Learning | Demonstration | Role Play Exercise | Audio or Video Review of Learner | Artificial Models/Simulation | Standardized Patients |
| --- | --- | --- | --- | --- | --- | --- | --- | --- | --- | --- | --- |
| Table 1 | 1- Gather a history and perform a physical exam |  |  |  |  |  |  |  |  |  |  |
|  | 2 - Prioritize a differential diagnosis |  |  |  |  |  |  |  |  |  |  |
|  | 3- Recommend/interpret common Tests |  |  |  |  |  |  |  |  |  |  |
| Table 2 | 4- Enter/discuss orders and Prescriptions |  |  |  |  |  |  |  |  |  |  |
|  | 5- Document a clinical encounter in the record |  |  |  |  |  |  |  |  |  |  |
|  | 6- Oral presentation of a clinical encounter |  |  |  |  |  |  |  |  |  |  |
| Table 3 | 7- Form clinical questions and retrieve evidence |  |  |  |  |  |  |  |  |  |  |
|  | 8- Give or receive handover to transition care |  |  |  |  |  |  |  |  |  |  |
| Table 4 | 9- Collaborate as part of an interprofessional team |  |  |  |  |  |  |  |  |  |  |
|  | 10- Recognize urgent/emergent care need and initiate evaluation and management |  |  |  |  |  |  |  |  |  |  |
| Table 5 | 11- Obtain informed consent for test or procedure |  |  |  |  |  |  |  |  |  |  |
|  | 12- Perform general procedures of a physician |  |  |  |  |  |  |  |  |  |  |
|  | 13- Identify system failures and contribute to safety and improvement culture |  |  |  |  |  |  |  |  |  |  |

**Module Design Worksheet Part 2**

EPA(s) selected: __________________________________________________

Educational method selected: ____________________________________________

Description of module:

(Consider learning objectives, number of learners you could accommodate, faculty/resource needs, time required, etc.)

**Module Design Worksheet Part 3**

| **Module Objective**  **(EPA/Milestone Targeted)** | **Time Available** | **Published Module Available** | **Module Leaders and Faculty Required** | **Supplies, Space, Resources Needed** | **Assessment and Evaluation Tools** |
| --- | --- | --- | --- | --- | --- |
|  |  |  |  |  |  |
|  |  |  |  |  |  |
|  |  |  |  |  |  |
|  |  |  |  |  |  |
|  |  |  |  |  |  |
|  |  |  |  |  |  |
|  |  |  |  |  |  |
|  |  |  |  |  |  |
|  |  |  |  |  |  |
|  |  |  |  |  |  |
|  |  |  |  |  |  |
|  |  |  |  |  |  |
|  |  |  |  |  |  |
|  |  |  |  |  |  |
|  |  |  |  |  |  |
|  |  |  |  |  |  |
|  |  |  |  |  |  |
|  |  |  |  |  |  |

**Matching Educational Methods to Objectives* Summary**

| **Educational Method** | **Cognitive: Knowledge** | **Cognitive:**  **Problem Solving** | **Affective: Attitudinal** | **Psychomotor:**  **Skill** | **Psychomotor: Behavior** |
| --- | --- | --- | --- | --- | --- |
| Readings | +++ | + | + | + |  |
| Lectures | +++ | + | + | + |  |
| Online learning resources | +++ | ++ | + | + |  |
| Group discussions | ++ | ++ | +++ | + | + |
| Problem-based learning | ++ | +++ | + | + | + |
| Team-based learning | +++ | +++ | ++ | + | + |
| Peer teaching | +++ | +++ | ++ | + | + |
| Real-life and supervised clinical experiences | + | ++ | ++ | +++ | ++ |
| Reflection on experience | + | + | +++ | + | ++ |
| Role models | + | + | +++ | + | ++ |
| Demonstration | + | + | +++ | ++ | + |
| Simulation and task trainers | + | ++ | ++ | +++ | ++ |
| Role-plays | + | + | +++ | +++ | ++ |
| Standardized patients | + | ++ | ++ | +++ | ++ |
| Audio or video review of learner |  |  | ++ | +++ | +++ |
| Behavioral/environmental interventions |  |  | + | + | +++ |

+ = sometimes appropriate, often as an adjunct to other methods

++ = good match

+++ = excellent match

Blank = not recommended

*Adapted from Thomas PA, Abras CN. Step 4: Educational Strategies. In: Thomas, PA, Kern DE, Hughes, MT, Chen BY, eds*. Curriculum Development of Medical Education: A Six-Step Approach*. 3rd ed. Baltimore, MD: Johns Hopkins University Press; 2015: 65-101.

**Module Design Worksheet Part 1 Example**

Identify the educational methods most useful/applicable to the EPA(s) you would like to address.

On Part 2, select one (or more!) EPAs and one method to design a module for a Pediatric Boot Camp.

|  | EPA | Lecture or Reading | Programmed Learning | Discussion | Reflection or Writing Experience | Game, Team or Problem Based Learning | Demonstration | Role Play Exercise | Audio or Video Review of Learner | Artificial Models/Simulation | Standardized Patients |
| --- | --- | --- | --- | --- | --- | --- | --- | --- | --- | --- | --- |
|  | 1- Gather a history and perform a physical exam |  |  |  |  | X | X | X | X | X | X |
|  | 2 - Prioritize a differential diagnosis |  |  | X |  | X |  |  |  | X | X |
|  | 3- Recommend/interpret common Tests |  |  | X |  | X |  |  |  | X |  |
|  | 4- Enter/discuss orders and Prescriptions |  | X |  |  | X |  | X |  |  |  |
|  | 5- Document a clinical encounter in the record |  |  | X |  |  | X |  |  |  |  |
|  | 6- Oral presentation of a clinical encounter |  |  |  |  |  | X |  | X | X | X |
|  | 7- Form clinical questions and retrieve evidence | X | X | X | X |  |  |  |  |  |  |
|  | 8- Give or receive handover to transition care |  |  |  |  |  | X | X | X | X |  |
|  | 9- Collaborate as part of an interprofessional team |  | X |  |  |  |  |  |  |  |  |
|  | 10- Recognize urgent/emergent care need and initiate evaluation and management |  |  |  |  |  |  |  |  | X |  |
|  | 11- Obtain informed consent for test or procedure |  |  |  |  |  |  | X |  | X | X |
|  | 12- Perform general procedures of a physician |  |  |  |  |  | X | X |  |  |  |
|  | 13- Identify system failures and contribute to safety and improvement culture |  |  | X | X | X |  |  |  |  |  |

**Module Design Worksheet Part 2 Example**

EPA selected: *Identify system failures and contribute to a safety and improvement culture*

Educational method selected: *Game*

Description of module:

(Consider learning objectives, number of learners you could accommodate, faculty/resource needs, time required, etc.)

*Use a patient scenario of an adverse event.*

*Set up room with multiple system failures; for example, no easily available bag valve mask ventilation system, frequent alarms, incorrect medication, sign that says “prefers xxxx language,” and a new resident who does not know the system or the team.*

*Objectives:*

1. *Reviewing the patient environment, list 5 factors that contributed to the adverse event.*
2. *Using the Swiss cheese model to analyze medical errors, identify possible systems errors contributing to the adverse event.*
3. *Write a reflection piece on examples that could improve the errors.*

*1 hour session:*

*Rotate through patient room for 10 minutes.*

*Read/watch literature on Swiss cheese model and safety language 20 minutes*

*Write for 30 minutes.*

*1 facilitator required*

*1 patient room required*

**Module Design Worksheet Part 3 Example**

| **Module Objective**  **(EPA/Milestone Targeted)** | **Time Available** | **Published Module Available** | **Module Leaders and Faculty Required** | **Supplies, Space, Resources Needed** | **Assessment and Evaluation Tools** |
| --- | --- | --- | --- | --- | --- |
| *Obtain informed Consent for test or procedure  *Perform general procedures of a physician | 1:30 | No, but video about infant LP available from INSPIRE* | 2 | Computer, projector, slide deck on informed consent, video about LP, 3 infant LP task trainers, 3 LP kits, extra LP needles, surgical gloves x 12 students, simulation lab or room with 3 tables | Students observe each other and provide feedback based in checklist developed by INSPIRE |
| *Collaborate as part of an interprofessional team *Give or receive handover to transition care | 3:00 | Yes. Burns R, et al. Pediatric boot camp series: assessment and plans, task prioritization, answering pages, handoffs. MedEdPORTAL Publications. 2015;11:10310. | 3 | Computer, projector, slide decks on calling consultants and handoffs, cases, facilitator guides, room with space for 3 small groups | Peer and facilitator feedback in real-time |
|  |  |  |  |  |  |
|  |  |  |  |  |  |
|  |  |  |  |  |  |
|  |  |  |  |  |  |
|  |  |  |  |  |  |
|  |  |  |  |  |  |
|  |  |  |  |  |  |

*INSPIRE-IPSS: International Network for Simulation-based Pediatric Innovation, Research, & Education
