# Supplementary material for: Building a Boot Camp: Pediatric Residency Preparatory Course Design Workshop and Tool Kit
Source: MedEdPORTAL. 2019 Dec 13;15:10860. doi: 10.15766/mep_2374-8265.10860 (PMC7010200; doi:10.15766/mep_2374-8265.10860)
Supplement: Supplementary file 1 — A. Boot Camp Workshop Presentation.pptx B. Review of Existing Boot Camp Literature.docx C. Institutional Needs Assessment Worksheet.docx D. Recommended Content List and Session Prioritization Worksheet.docx E. Schedule Worksheet and Sample Schedules.docx F. Module Design Worksheet and Planning Resources.docx G. Selected MedEdPORTAL Boot Camp Resources.docx H. Workshop Feedback Surveys.docx I. Facilitator Guide.docx [file mep-15-10860-s001.zip › G. Selected MedEdPORTAL Boot Camp Resources.docx]

| **Bootcamp/MS4 specific^1-6^** | |
| --- | --- |
| - [Pediatric Boot Camp Series: Obtaining a Consult, Discussing Difficult News](http://www.mededportal.org/publication/10437/) | |
| - [Pediatric Boot Camp Series: Assessment and Plans, Task Prioritization, Answering Pages, Handoffs](https://www.mededportal.org/publication/10310/)  - [Pediatric Boot Camp Series: Infant With Altered Mental Status and Seizure—A Case of Child Abuse](https://www.mededportal.org/publication/10552/) | |
| - [Interactive Pediatric Cases for Senior Capstone Courses](https://www.mededportal.org/publication/7743/) | |
| - [Surgery Boot Camp](https://www.mededportal.org/publication/9820/) (for complete surgical curriculum see also: [ACS/APDS/ASE Resident Prep Curriculum](https://www.facs.org/education/program/resident-prep)) | |
| - [One Night On-Call: A Simulation Exercise for New Interns](http://www.mededportal.org/publication/1760/) (Internal Medicine example) | |
| **Communication^7-20^** | |
| Peer/Handoff | - [Handling the Handoff: An Evidence-Based Approach](https://www.mededportal.org/publication/10035/#319526) |
|  | -I-PASS Handoff Curriculum ([Core Resident Workshop](https://www.mededportal.org/publication/9311/), [Medical Student Workshop](https://www.mededportal.org/publication/9854/)) |
|  | - [Team-Based Simulation for Medical Student Handoff Education](https://www.mededportal.org/publication/10486/#287518) |
| Inter professional | - [Crucial Conversations and Conflict Resolution Interprofessional Simulation](https://www.mededportal.org/publication/10063/#319940) |
|  | - [Interprofessional Care: an Introductory Session on the Roles of Health Professionals](https://www.mededportal.org/publication/9813/#305719) |
| Family | - [Bad News Deserves Better Communication: A Customizable Curriculum for Teaching Learners to -Share Life-Altering Information in Pediatrics](https://www.mededportal.org/publication/10438/) |
|  | - [Simulated Pediatric Physician-Parent Email Cases](https://www.mededportal.org/publication/9082/) |
|  | - [How to Communicate Value Added Care: Negotiation and Saying No](https://www.mededportal.org/publication/9894/#314042) |
|  | - [A Flipped Classroom and Case-Based Curriculum to Prepare Medical Students for Vaccine-Related Conversations with Parents](https://www.mededportal.org/publication/10582/#289624) |
|  | - [A Breaking Bad News Exercise to Assess Student Competence Prior to Graduation (Internal Medicine example)](https://www.mededportal.org/publication/10015/#314174) |
| General Communication | - [Can We Talk? - Case Studies In Communication Challenges And Conflict Management for Clinicians](https://www.mededportal.org/publication/7957/#316517) |
|  | - [Techniques for Teaching Communication Skills: Developing "Great Communicators"](https://www.mededportal.org/publication/9974/#308256) |
|  | - [Teaching Conflict Resolution in Medicine: Lessons From Business, Diplomacy, and Theatre](https://www.mededportal.org/publication/10672/) |
| **Critical Thinking/Processing^21-25^** | |
| - [Differential Diagnosis: Approaches and Pitfalls - A Pediatric Case-Based Session for 3rd Year Medical Students](https://www.mededportal.org/publication/9640/#309689) | |
| - [Interpretation of Data Workshop in the Pediatric Preclerkship Educational Exercises (PRECEDE) Curriculum](https://www.mededportal.org/publication/10496/) | |
| - [Decision - Diagnosis: An Introduction to Diagnostic Error and Medical Decision-Making](https://www.mededportal.org/publication/10378/#293646) | |
| - [Teaching Cognitive Biases in Clinical Decision Making: A Case-Based Discussion](https://www.mededportal.org/publication/10138/) (EM example) | |
| - [Efficiency in the Pediatric Emergency Department: A Choose Your Own Adventure for Pediatric Residents and Medical Students](https://www.mededportal.org/publication/10214/) | |
| **Professionalism/Burnout^26-28^** | |
| - [“Time to Talk About It: Physician Depression and Suicide” Video/Discussion Session for Interns, Residents, and Fellows](https://www.mededportal.org/publication/10508/) | |
| - [A Curriculum for Clerkship Students to Foster Professionalism Through Reflective Practice and Identity Formation](https://www.mededportal.org/publication/10416/) | |
| - [Social Media: Social Intelligence Training Module](https://www.mededportal.org/publication/10442/) | |
| **Procedures^29-34^** | |
| General | - [A Clinical Procedures Course for Medical Students](https://www.mededportal.org/publication/10524/) |
|  | - [Orientation to Emergency Pediatric Procedures](https://www.mededportal.org/publication/9852/) |
| LP | - [A Comprehensive Infant Lumber Puncture Novice Procedural Skills Training Package: An INSPIRE Simulation-Based Procedural Skills Training Package](https://www.mededportal.org/publication/9724/) |
|  | - [Lumbar Puncture Teaching Skills Objective Structured Clinical Examination (OSCE) Station](https://www.mededportal.org/publication/9800/) |
|  | - [OSATS Assessment Instrument for Neonatal Lumbar Puncture](https://www.mededportal.org/publication/9758/) |
| Intubation | - [OSATS Tool for Pediatric Rapid Sequence Intubation](https://www.mededportal.org/publication/9727/) |
|  | |
| **Medical Knowledge^35-68^** | |
| Task/Skills | - [How to Write Prescriptions](https://www.mededportal.org/publication/9982/) |
| EKG | - [Using a Case Scenario-based Self-Teaching Module to Increase Overall Skill in ECG Interpretation for Pediatric Residents](https://www.mededportal.org/publication/9648/) |
| Simulation and Podcast Series | - [PedsCases Learning Module for Medical Students](https://www.mededportal.org/search/?q=PedsCases)  ([Adol Med](https://www.mededportal.org/publication/7949/), [Development](https://www.mededportal.org/publication/7988/#264803), [Failure to Thrive](https://www.mededportal.org/publication/7912/#264423), [Stridor](https://www.mededportal.org/publication/8276/#266218), [Neonatology](https://www.mededportal.org/publication/7991/#264829), and other topics) |
|  | - [Four Core Cases: A simulation curriculum for pediatrics residents](https://www.mededportal.org/publication/9943/#314450) |
|  | - Pediatric Emergency Medicine Simulation Curriculum (Series: [Anaphylaxis](https://www.mededportal.org/publication/9638/), [Seizures](https://www.mededportal.org/publication/9794/), [Septic Shock](https://www.mededportal.org/publication/9639/), [Status Asthmaticus](https://www.mededportal.org/publication/9660/), [Bronchiolitis](https://www.mededportal.org/publication/10012/), [SVT](https://www.mededportal.org/publication/9716/#276714), and other topics) |
|  | - [Rapid Cycle Deliberate Practice Pediatric Simulation Scenarios](https://www.mededportal.org/publication/10134/) |
| AMS | - [Pediatric Altered Mental Status](https://www.mededportal.org/publication/9868/)  - [Altered Mental Status in an Adolescent Patient](https://www.mededportal.org/publication/525/) |
| Non Accidental Trauma | - [A Pediatric Death from Non-Accidental Trauma](https://www.mededportal.org/publication/10064/#319953) |
| Neonatal Fever/ Emergencies | - [Febrile Young Infant Learning Module](https://www.mededportal.org/publication/9568/) |
|  | - [Fever and Seizure in a Young Infant: A Simulation Case](https://www.mededportal.org/publication/10468/) |
|  | - [Lethargic Infant](https://www.mededportal.org/publication/755/) |
|  | - [Simulation Case: Neonate With Fever Requiring IO Line](https://www.mededportal.org/publication/8391/) |
|  | - [Rapid Cycle Deliberate Practice: Application to Neonatal Resuscitation](https://www.mededportal.org/publication/10534/) |
|  | - [Simulation Based Education in Pediatrics: The Febrile Neonate](https://www.mededportal.org/publication/9837/) |
| Jaundice | - [Infant with Jaundice: A Pediatric Standardized Patient OSCE Case](https://www.mededportal.org/publication/9921/) |
| PALS-type scenarios | -Rapid Cycle Deliberate Practice Pediatric Simulation Scenarios (see sim series above) |
|  | - [Pediatric Pulseless Ventricular Tachycardia: A Simulation Scenario for Fellows, Residents, Medical Students, and Advanced Practitioners](https://www.mededportal.org/publication/10407/) |
|  | - [Use of Simulation to Improve Cardiopulmonary Resuscitation Performance and Code Team Communication for Pediatric Residents](https://www.mededportal.org/publication/10555/) |
|  | - [Flipped Classroom Module on Shock for Medical Students](https://www.mededportal.org/publication/10542/) |
|  | - [Acute Respiratory Emergencies in Pediatrics](https://www.mededportal.org/publication/8143/) |
|  | - [Simulation of Airway Management for the Pediatric Resident](https://www.mededportal.org/publication/9881/) |
|  | - [Hypovolemic Shock in a Child: A Pediatric Simulation Case](https://www.mededportal.org/publication/10694/) |
| Seizures | - [Simulation of Seizures for the Pediatrics Resident](https://www.mededportal.org/publication/9880/) |
| Well Child/ Development | - [Pediatric Well-Child Interview Standardized Patient Scenarios](https://www.mededportal.org/publication/9725/) |
|  | - [Standardized Patient Case: Health Supervision Encounter for a Child 6-24 Months of Age](https://www.mededportal.org/publication/9192/) |
| **System based practice^69-75^** | |
| -An Integrated Practical Evidence-Based Medicine Curriculum Series([Forming and Searching PICO Questions](https://www.mededportal.org/publication/9446/), [Diagnostic tests](https://www.mededportal.org/publication/9663/#309820), [Systematic Reviews and Meta-analyses](https://www.mededportal.org/publication/9835/), [Harm/Causation and Prognosis](https://www.mededportal.org/publication/9788/) , [Therapy](https://www.mededportal.org/publication/9750/)) | |
| - [Diagnosis Coding for Clinicians: Core Knowledge and Transition to ICD-10](https://www.mededportal.org/publication/9823/) | |
| - [High Value Care Pediatric Curriculum](https://www.mededportal.org/publication/10146/) | |
|  | |

**References:**

1. Burns R, Mangold K, Adler M, Trainor J. Pediatric boot camp series: obtaining a consult, discussing difficult news. MedEdPORTAL. 2016;12:10437. <https://doi.org/10.15766/mep_2374-8265.10437>
2. Burns R, Nicholson A, Mangold K, Adler M, Trainor J. Pediatric boot camp series: assessment and plans, task prioritization, answering pages, handoffs. MedEdPORTAL. 2015;11:10310. <https://doi.org/10.15766/mep_2374-8265.10310>
3. Metz J, Stone K, Reid J, Burns R. Pediatric boot camp series: infant with altered mental status and seizure—a case of child abuse. MedEdPORTAL. 2017;13:10552. <https://doi.org/10.15766/mep_2374-8265.10552>
4. Smith S, Hayward K, Kronman M. Interactive pediatric cases for senior capstone courses. MedEdPORTAL. 2009;5:7743. <https://doi.org/10.15766/mep_2374-8265.7743>
5. Issa N, Fryer J, Swaroop M, et al. Surgery boot camp. MedEdPORTAL. 2014;10:9820. <https://doi.org/10.15766/mep_2374-8265.9820>
6. Vincent D, Berg B. One night on-call: a simulation exercise for new interns. MedEdPORTAL. 2009;5:1760. <https://doi.org/10.15766/mep_2374-8265.1760>
7. Goldsmith A. Handling the handoff: an evidence-based approach. MedEdPORTAL. 2015;11:10035. <https://doi.org/10.15766/mep_2374-8265.10035>
8. Spector N, Starner A, Allen A, et al. I-PASS handoff curriculum: core resident workshop. MedEdPORTAL. 2013;9:9311. <https://doi.org/10.15766/mep_2374-8265.9311>
9. O'Toole J, Calaman S, Everhart J, et al. I-PASS handoff curriculum: medical student workshop. MedEdPORTAL. 2014;10:9854. <https://doi.org/10.15766/mep_2374-8265.9854>
10. Higgins Joyce A. Team-based simulation for medical student handoff education. MedEdPORTAL. 2016;12:10486. <https://doi.org/10.15766/mep_2374-8265.10486>
11. Shrader S, Zaudke J. Crucial conversations and conflict resolution interprofessional simulation. MedEdPORTAL. 2015;11:10063. <https://doi.org/10.15766/mep_2374-8265.10063>
12. Durham M, Lie D, Lohenry K. Interprofessional care: an introductory session on the roles of health professionals. MedEdPORTAL. 2014;10:9813. <https://doi.org/10.15766/mep_2374-8265.9813>
13. Wolfe AD, Denniston SF, Baker J, Catrine K, Hoover-Regan M. Bad news deserves better communication: a customizable curriculum for teaching learners to share life-altering information in pediatrics. MedEdPORTAL. 2016;12:10438. <https://doi.org/10.15766/mep_2374-8265.10438>
14. Schiller J, Christner J, Keefer P, et al. Simulated pediatric physician-parent email cases. MedEdPORTAL. 2012;8:9082. <https://doi.org/10.15766/mep_2374-8265.9082>
15. Duke P, Cochran N. How to communicate value added care: negotiation and saying no. MedEdPORTAL. 2014;10:9894. <https://doi.org/10.15766/mep_2374-8265.9894>
16. Coleman A, Lehman D. A flipped classroom and case-based curriculum to prepare medical students for vaccine-related conversations with parents. MedEdPORTAL. 2017;13:10582. <https://doi.org/10.15766/mep_2374-8265.10582>
17. Clay A, Ross E, Knudsen N, Chudgar S, Engle D, Grochowski C. A breaking bad news exercise to assess student competence prior to graduation. MedEdPORTAL. 2015;11:10015. <https://doi.org/10.15766/mep_2374-8265.10015>
18. Goldberg C, Tanabe T, Plato M. Can we talk? - case studies in communication challenges and conflict management for clinicians. *MedEdPORTAL*. 2011;7:7957. <https://doi.org/10.15766/mep_2374-8265.7957>
19. Harris T, Ismail N, Loboprabhu S, et al. Techniques for teaching communication skills: developing "great communicators". MedEdPORTAL. 2014;10:9974. <https://doi.org/10.15766/mep_2374-8265.9974>
20. Wolfe AD, Hoang KB, Denniston SF. Teaching conflict resolution in medicine: lessons from business, diplomacy, and theatre. MedEdPORTAL. 2018;14:10672. <https://doi.org/10.15766/mep_2374-8265.10672>
21. Waldman Z, Ottolini M. Differential diagnosis: approaches and pitfalls - a pediatric case-based session for 3rd year medical students. MedEdPORTAL. 2013;9:9640. <https://doi.org/10.15766/mep_2374-8265.9640>

References Continued:

1. Balighian E, Barone M, Cooke D, et al. Interpretation of data workshop in the pediatric preclerkship educational exercises (PRECEDE) curriculum. MedEdPORTAL. 2016;12:10496. <https://doi.org/10.15766/mep_2374-8265.10496>
2. Ruedinger E, Mathews B, Olson A. Decision - diagnosis: an introduction to diagnostic error and medical decision-making. MedEdPORTAL. 2016;12:10378. <https://doi.org/10.15766/mep_2374-8265.10378>
3. Chew K, van Merrienboer J, Durning S. Teaching cognitive biases in clinical decision making: a case-based discussion. MedEdPORTAL. 2015;11:10138. <https://doi.org/10.15766/mep_2374-8265.10138>
4. Witkowski J, Rassbach C. Efficiency in the pediatric emergency department: a choose your own adventure for pediatric residents and medical students. MedEdPORTAL. 2015;11:10214. <https://doi.org/10.15766/mep_2374-8265.10214>
5. Nagy C, Schwabe D, Jones W, et al. “Time to Talk About It: Physician Depression and Suicide” video/discussion session for interns, residents, and fellows. MedEdPORTAL. 2016;12:10508. <https://doi.org/10.15766/mep_2374-8265.10508>
6. Glod SA, Richard D, Gordon P, et al. A curriculum for clerkship students to foster professionalism through reflective practice and identity formation. MedEdPORTAL. 2016;12:10416. <https://doi.org/10.15766/mep_2374-8265.10416>
7. Robertson M, Shoss MK, Broom MA. Social media: social intelligence training module. MedEdPORTAL. 2016;12:10442. <https://doi.org/10.15766/mep_2374-8265.10442>
8. Romeo R, Blasiole B, Chalifoux T, et al. A clinical procedures course for medical students. MedEdPORTAL. 2016;12:10524. <https://doi.org/10.15766/mep_2374-8265.10524>
9. Nestor-Arjun B, Miano M. Orientation to emergency pediatric procedures. MedEdPORTAL. 2014;10:9852. <https://doi.org/10.15766/mep_2374-8265.9852>
10. Auerbach M, Chang T, Fein D, et al. A comprehensive infant lumber puncture novice procedural skills training package: an inspire simulation-based procedural skills training package. MedEdPORTAL. 2014;10:9724. <https://doi.org/10.15766/mep_2374-8265.9724>
11. Smyth P, Jeerakathil T, Roberts T. Lumbar puncture teaching skills objective structured clinical examination (OSCE) station. MedEdPORTAL. 2014;10:9800. <https://doi.org/10.15766/mep_2374-8265.9800>
12. House J, Iyer M, Santen S, Warrier K, et al. OSATS assessment instrument for neonatal lumbar puncture. MedEdPORTAL. 2014;10:9758. <https://doi.org/10.15766/mep_2374-8265.9758>
13. House J, Dooley-Hash S, Hamstra S, Nypaver M. OSATS tool for pediatric rapid sequence intubation. MedEdPORTAL. 2014;10:9727. <https://doi.org/10.15766/mep_2374-8265.9727>
14. Ekong M, Frazier J, Oholendt K. How to write prescriptions. MedEdPORTAL. 2014;10:9982. <https://doi.org/10.15766/mep_2374-8265.9982>
15. Weinberg J, Ottolini M, Sestokas J, Greene E. Using a case scenario-based self-teaching module to increase overall skill in ECG interpretation for pediatric residents. MedEdPORTAL. 2013;9:9648. <https://doi.org/10.15766/mep_2374-8265.9648>
16. Kitney L, Gill P, Lewis M, Leslie K, Steinegger C. PedsCases - a learning module for adolescent medicine for medical students. MedEdPORTAL. 2010;6:7949. <https://doi.org/10.15766/mep_2374-8265.7949>
17. Gill P, Andrews D. PedsCases - a learning module for the developmental assessment in children for medical students. MedEdPORTAL. 2010;6:7988. <https://doi.org/10.15766/mep_2374-8265.7988>
18. MacPherson P, Lewis M. PedsCases - a learning module for the evaluation of a child with failure to thrive for medical students. MedEdPORTAL. 2010;6:7912. <https://doi.org/10.15766/mep_2374-8265.7912>
19. Gerdung C, Lewis M, Duff J, Graham T, Grewal S. PedsCases - a learning module of acute stridor for medical students. MedEdPORTAL. 2011;7:8276. <https://doi.org/10.15766/mep_2374-8265.8276>
20. Gill P, MacPherson P, Lewis M, Joynt C. PedsCases - a learning module of neonatology cases for medical students. MedEdPORTAL. 2010;6:7991. <https://doi.org/10.15766/mep_2374-8265.7991>
21. Sagalowsky S, Boyle T, Winn A, et al. Four core cases: a simulation curriculum for pediatrics residents. MedEdPORTAL. 2014;10:9943. <https://doi.org/10.15766/mep_2374-8265.9943>

References Continued:

1. Reid J, Stone K. Pediatric emergency medicine simulation curriculum: anaphylaxis. *MedEdPORTAL*. 2013;9:9638. <https://doi.org/10.15766/mep_2374-8265.9638>
2. Reid J, Stone K. Pediatric emergency medicine simulation curriculum: seizure scenario. *MedEdPORTAL*. 2014;10:9794. <https://doi.org/10.15766/mep_2374-8265.9794>
3. Reid J, Stone K. Pediatric emergency medicine simulation curriculum: septic shock. *MedEdPORTAL*. 2013;9:9639. <https://doi.org/10.15766/mep_2374-8265.9639>
4. Reid J, Stone K. Pediatric emergency medicine simulation curriculum: status asthmaticus. *MedEdPORTAL*. 2014;10:9660. <https://doi.org/10.15766/mep_2374-8265.9660>
5. Uspal N, Stone K, Reid J, Coleman-Satterfield T. Pediatric emergency medicine simulation curriculum: bronchiolitis. *MedEdPORTAL*. 2015;11:10012. <https://doi.org/10.15766/mep_2374-8265.10012>
6. Stone K, Reid J. Pediatric emergency medicine simulation curriculum: supraventricular tachycardia. *MedEdPORTAL*. 2014;10:9716. <https://doi.org/10.15766/mep_2374-8265.9716>
7. Doughty C, Welch-Horan T, Hsu D, et al. Rapid cycle deliberate practice pediatric simulation scenarios. *MedEdPORTAL*. 2015;11:10134. <https://doi.org/10.15766/mep_2374-8265.10134>
8. Wolff M, Skaugset L, Jachowski J, Rogers A. Pediatric altered mental status. *MedEdPORTAL*. 2014;10:9868. <https://doi.org/10.15766/mep_2374-8265.9868>
9. Kobayashi L, Shapiro M. Altered mental status in an adolescent patient. *MedEdPORTAL*. 2009;5:525. <https://doi.org/10.15766/mep_2374-8265.525>
10. Beattie L, Ryan M, Rowe J, Mazin R. A pediatric death from non-accidental trauma. *MedEdPORTAL*. 2015;11:10064. <https://doi.org/10.15766/mep_2374-8265.10064>
11. Wolff M, Pomeranz E, Carney M. Febrile young infant learning module. *MedEdPORTAL*. 2013;9:9568. <https://doi.org/10.15766/mep_2374-8265.9568>
12. Rideout M, Raszka W. Fever and seizure in a young infant: a simulation case. *MedEdPORTAL*. 2016;12:10468. <https://doi.org/10.15766/mep_2374-8265.10468>
13. Noeller T, Smith M. Lethargic infant. *MedEdPORTAL*. 2007;3:755. <https://doi.org/10.15766/mep_2374-8265.755>
14. Solway M, Wilbur L, Walthall J. Simulation case: neonate with fever requiring IO line. *MedEdPORTAL*. 2011;7:8391. <https://doi.org/10.15766/mep_2374-8265.8391>
15. Patricia K, Arnold J, Lemke D. Rapid cycle deliberate practice: application to neonatal resuscitation. *MedEdPORTAL*. 2017;13:10534. <https://doi.org/10.15766/mep_2374-8265.10534>
16. Dudas R, Cooke D, Stewart R, Balighian E, Golden W, Barone M. Simulation based education in pediatrics: the febrile neonate. *MedEdPORTAL*. 2014;10:9837. <https://doi.org/10.15766/mep_2374-8265.9837>
17. Steffey C, Merricks P. Infant with jaundice: a pediatric standardized patient OSCE case. *MedEdPORTAL*. 2014;10:9921. <https://doi.org/10.15766/mep_2374-8265.9921>
18. Cashen K, Petersen T. Pediatric pulseless ventricular tachycardia: a simulation scenario for fellows, residents, medical students, and advanced practitioners. *MedEdPORTAL*. 2016;12:10407. <https://doi.org/10.15766/mep_2374-8265.10407>
19. Couloures KG, Allen C. Use of simulation to improve cardiopulmonary resuscitation performance and code team communication for pediatric residents. *MedEdPORTAL*. 2017;13:10555. <https://doi.org/10.15766/mep_2374-8265.10555>
20. Hoffmann JA, Thompson RW. Flipped classroom module on shock for medical students. *MedEdPORTAL*. 2017;13:10542. <https://doi.org/10.15766/mep_2374-8265.10542>
21. Calaman S, Haines C. Acute respiratory emergencies in pediatrics. *MedEdPORTAL*. 2010;6:8143. <https://doi.org/10.15766/mep_2374-8265.8143>
22. Friedman S, Tozzi M, Siems A, Carey A, Moerdler S, Zackai S. Simulation of airway management for the pediatric resident. *MedEdPORTAL*. 2014;10:9881. <https://doi.org/10.15766/mep_2374-8265.9881>

References Continued:

1. Rideout M, Raszka W. Hypovolemic shock in a child: a pediatric simulation case. *MedEdPORTAL*. 2018;14:10694. <https://doi.org/10.15766/mep_2374-8265.10694>
2. Friedman S, Tozzi M, Shakhin V, et al. Simulation of seizures for the pediatrics resident. *MedEdPORTAL*. 2014;10:9880. <https://doi.org/10.15766/mep_2374-8265.9880>
3. Elliott E. Pediatric well-child interview standardized patient scenarios. *MedEdPORTAL*. 2014;10:9725. <https://doi.org/10.15766/mep_2374-8265.9725>
4. Balog E, Donovan M, Hanson J. Standardized patient case: health supervision encounter for a child 6-24 months of age. *MedEdPORTAL*. 2012;8:9192. <https://doi.org/10.15766/mep_2374-8265.9192>
5. Boykan R, Chitkara M, Kenefick C, Messina C. An integrated practical evidence-based medicine curriculum: two small group sessions to teach PICO question formation and searching strategies. *MedEdPORTAL*. 2013;9:9446. <https://doi.org/10.15766/mep_2374-8265.9446>
6. Chitkara M, Boykan R, Messina C. An integrated practical evidence-based medicine curriculum, critical appraisal: diagnostic test. *MedEdPORTAL*. 2014;10:9663. <https://doi.org/10.15766/mep_2374-8265.9663>
7. Chitkara M, Boykan R. An integrated practical evidence-based medicine curriculum, critical appraisal: systematic reviews and meta-analyses. *MedEdPORTAL*. 2014;10:9835. <https://doi.org/10.15766/mep_2374-8265.9835>
8. Boykan R, Chitkara M. An integrated practical evidence-based medicine curriculum, critical appraisal: harm/causation and prognosis. *MedEdPORTAL*. 2014;10:9788. <https://doi.org/10.15766/mep_2374-8265.9788>
9. Chitkara M, Boykan R. An integrated practical evidence-based medicine curriculum, critical appraisal: therapy. *MedEdPORTAL*. 2014;10:9750. <https://doi.org/10.15766/mep_2374-8265.9750>
10. Chick D, Andreae M. Diagnosis coding for clinicians: core knowledge and transition to ICD-10. *MedEdPORTAL*. 2014;10:9823. <https://doi.org/10.15766/mep_2374-8265.9823>
11. Woods S, Avery C, Bartlett K, et al. High value care pediatric curriculum. *MedEdPORTAL*. 2015;11:10146. <https://doi.org/10.15766/mep_2374-8265.10146>
