# Supplementary material for: Building a Boot Camp: Pediatric Residency Preparatory Course Design Workshop and Tool Kit
Source: MedEdPORTAL. 2019 Dec 13;15:10860. doi: 10.15766/mep_2374-8265.10860 (PMC7010200; doi:10.15766/mep_2374-8265.10860)
Supplement: Supplementary file 1 — A. Boot Camp Workshop Presentation.pptx B. Review of Existing Boot Camp Literature.docx C. Institutional Needs Assessment Worksheet.docx D. Recommended Content List and Session Prioritization Worksheet.docx E. Schedule Worksheet and Sample Schedules.docx F. Module Design Worksheet and Planning Resources.docx G. Selected MedEdPORTAL Boot Camp Resources.docx H. Workshop Feedback Surveys.docx I. Facilitator Guide.docx [file mep-15-10860-s001.zip › H. Workshop Feedback Surveys.docx]

**Building a Boot Camp:**

**Pediatric Residency Preparatory Course Design Workshop**

**Post- Workshop Survey**

Thank you for your participation in our workshop. Please take a few minutes to complete the following survey as a follow up to this workshop. Your responses will be used to improve future workshop presentations.

**1) Do you currently have a Boot Camp (Residency Preparatory Course) at your institution?**

a) Yes

b) No

**If you answered no to question 1, is your institution planning on developing a Boot Camp within the next 1-2 years?**

a) Yes

b) No

**2) What is the structure of your current or planned Boot Camp?**

- 1. Pediatric Specific
  2. General with a Pediatric Component/Section
  3. General
  4. Other (please specify)

**3) What is the duration of the pediatric component of your current or planned Boot Camp?**

- 1. 1 week or less
  2. >1 week and <4 weeks
  3. 4 or more weeks
  4. Undecided
  5. Other (please specify)

**4) What is the enrollment structure for your current or planned Boot Camp?**

- 1. Required for all M4 students
  2. Required for all M4 students entering Pediatric residency
  3. Required for all M4 students entering Pediatric and other selected residencies (EM, FM, etc)
  4. Elective for any M4 student
  5. Elective for any M4 student entering Pediatric residency
  6. Other (please specify)

**5) What is (will be) the timing of your Boot Camp?**

- 1. Longitudinal M4 course
  2. Prior to starting M4 year
  3. During M4 year but prior to Match
  4. Following Match
  5. Other (please specify)

**In reference to the workshop objectives, please answer the following questions regarding your confidence pertaining to Residency Preparatory Courses. You will be asked to rate your confidence both prior to and following participation in the workshop for each objective on a scale from 0 to 100.**

| **Component of Boot Camp Course Design** | **Your Confidence Level**  **0 (Not at all Confident)**  **100 (Extremely Confident)** | |
| --- | --- | --- |
|  | **Prior to the Workshop** | **Following the Workshop** |
| 6) Discussing the literature pertaining to residency preparatory courses |  |  |
| 7) Performing a needs assessment related to available resources, identified gaps, and institutional requirements |  |  |
| 8) Designing a residency preparatory course schedule aligned with individualized needs assessments |  |  |
| 9) Developing module ideas to use in residency preparatory courses that address core entrustable professional activities (EPA’s) |  |  |
| 10) Identifying barriers and strategies to successfully implement residency preparatory courses at your home institution |  |  |

**11) Name one thing you plan to implement from this workshop within the next year.**

**12) Is there any other input or feedback you would like to offer the presenters?**

**Thank you for your participation in both this survey and our workshop!**

**Building a Boot Camp:**

**Pediatric Residency Preparatory Course Design Workshop**

**Workshop Follow up Survey**

Thank you for your participation in our workshop. Please take a few minutes to complete the following survey as a follow up to this workshop. Your responses will be used to improve future workshop presentations.

**1) Please tell us about your institution’s boot camp course**

a) We had a boot camp before I attended the workshop

b) We are implementing a boot camp this academic year

c) We are planning a future boot camp

d) Undecided

**If you had a bootcamp before attending the workshop please answer the following, otherwise please skip to question 7:**

**2) Did you make any changes to an existing bootcamp based on information or materials from the workshop?**

a) Yes

b) No

**3) Please check any of the following that were helpful to you as you made changes to your boot camp**

a. Boot Camp Background Literature Review

b. Needs Assessment Worksheet Exercise

c. Example schedules from facilitators’ institutions

d. Sample Schedule Worksheet Exercise

e. Example MedEdPortal Boot Camp Module list

f. Module Design Worksheet Exercise based on selected EPAs

g. Implementation Strategies and Barriers Discussion

h. None

i. Other (please explain

**4) What specific changes did you make to your existing boot camp as a result of your participation in the workshop?**

**5) Which printed materials (if any) did you use following the workshop to help make changes to your existing boot camp?**

**6) How helpful do you think the materials and/or content from the workshop would be as a self-contained resource for faculty to use independently when planning a boot camp?**

| Not Helpful at all | Slightly Helpful | Moderately Helpful | Very Helpful | Extremely Helpful |
| --- | --- | --- | --- | --- |
| 1 | 2 | 3 | 4 | 5 |

**If you did not have a bootcamp before attending the workshop please answer the following questions:**

**7) Did completing the workshop assist you in planning or implementing a new boot camp?**

a) Yes

b) No

**8) Please check any of the following that were helpful to you as you planned or implemented a new boot camp**

a. Boot Camp Background Literature Review

b. Needs Assessment Worksheet Exercise

c. Example schedules from facilitators’ institutions

d. Sample Schedule Worksheet Exercise

e. Example MedEdPortal Boot Camp Module list

f. Module Design Worksheet Exercise based on selected EPAs

g. Implementation Strategies and Barriers Discussion

h. None

i. Other (please explain)

**9) Please specify how your participation in the workshop helped you develop a boot camp?**

**10) Which printed materials (if any) did you use following the workshop in developing a boot camp?**

**11) How helpful do you think the materials and/or content from the workshop would be as a self-contained resource for faculty to use independently when planning a boot camp?**

| Not Helpful at all | Slightly Helpful | Moderately Helpful | Very Helpful | Extremely Helpful |
| --- | --- | --- | --- | --- |
| 1 | 2 | 3 | 4 | 5 |

**Thank you for your participation in both this survey and our workshop!**
