# Supplementary material for: Building a Boot Camp: Pediatric Residency Preparatory Course Design Workshop and Tool Kit
Source: MedEdPORTAL. 2019 Dec 13;15:10860. doi: 10.15766/mep_2374-8265.10860 (PMC7010200; doi:10.15766/mep_2374-8265.10860)
Supplement: Supplementary file 1 — A. Boot Camp Workshop Presentation.pptx B. Review of Existing Boot Camp Literature.docx C. Institutional Needs Assessment Worksheet.docx D. Recommended Content List and Session Prioritization Worksheet.docx E. Schedule Worksheet and Sample Schedules.docx F. Module Design Worksheet and Planning Resources.docx G. Selected MedEdPORTAL Boot Camp Resources.docx H. Workshop Feedback Surveys.docx I. Facilitator Guide.docx [file mep-15-10860-s001.zip › I. Facilitator Guide.docx]

**Appendix I: Facilitator Guide for Workshop Presentation**

**Recommendations and Practical Instructions for Workshop Sections:**

1. Prior to the workshop:
   1. Review of materials: As preparation for facilitating this workshop, we suggest reviewing the Background Literature (Appendix B) and then working through the subsequent sections of the workshop as an individual user. This will give you familiarity with the resource materials and the progression of the workshop. For each of the exercises, the appendix contains both a blank and a representative completed sample. We intended these samples for use by facilitators or individuals using this toolkit outside of the workshop setting, however you may provide them to participants if you chose.
   2. Preregistration: This workshop can be scaled to accommodate up to approximately 40 participants. Larger groups may require additional time for report-out / group discussions. Since the majority of the workshop activities include handouts, we strongly suggest having participants preregister to allow for accurate printing of materials and room accommodations.
   3. Handouts: Since most workshop sections have associated handouts which can become disorganized/overwhelming if given all at once, it may be useful to hold them in reserve and distribute at the start of the section. Alternatively, handouts could be provided in a binder organized by activity.
   4. Facilities: Round tables seating 6-8 work best to allow group work and discussion including smaller 3-4 person subgroups for some activities. Each table should have an unobstructed view of the slideshow.
   5. Roles: If you have multiple facilitators conducting the workshop, consider assigning lead presenter roles for each section, as well as someone to monitor time and distribute handouts. Facilitators can also assign themselves to table(s) to visit during the exercises as a resource to the participants.
2. Introduction, Audience Identification, and Review of Objectives
   1. Facilitator experience: It may be helpful to start by describing your own interest/experience in Boot Camps. For some facilitators, your prior expertise may be in curriculum development rather than specifically in Boot Camps.
   2. Participant experience: Depending on your venue, your audience may vary in terms of knowledge of curriculum design or existing boot camp literature or may even be clinicians from different clinical fields. Getting a sense of the prior experience allows you to determine which workshop segments may need more/less emphasis and can be helpful in dividing participants for each of the exercises. Appendix A Slides 2-3 can be used to do this in a quick show of hands and to prompt expanded discussion as time / group size allows. If the audience members have existing boot camp courses and this workshop is intended to help in revising and refining these courses, please see the “Existing Boot Camps” Section at the end of this guide.
   3. Objective Review: These may be read directly from Appendix A slide 4. If a significant number of your participants have existing boot camps, you may add that they can approach the workshop content as a re-evaluation of the planning and implementation they used to identify room for improvement. Please also see the “Existing Boot Camps” Section at the end of this guide.
3. Background Literature

On Slides 5-10 of the PowerPoint presentation in Appendix A, there is a brief overview of the background literature for Boot Camps. The slide text is purposefully brief to encourage discussion, and talking points are included in the notes section within each slide. For additional details, please see the more in depth review and references in Appendix B. Appendix A Slides 11-14 include multi-institution survey results obtained by the Council on Medical Student Education in Pediatrics (COMSEP). These are included for comparison and to highlight the variety of curriculum structures in use, but may be eliminated if less applicable to your participants (eg single institution workshop with multiple specialties represented).

1. Needs Assessment

Start by show of hands (Appendix A slide 15), and if participants have previously conducted a needs assessment, discuss whether this was for a boot camp or another course. Appendix A Slides 16-18 and 20 then give additional prompts to common components of needs assessments for boot camps as an introduction to the exercise. If your workshop size is small or all from a single institution, consider expanding peer sharing to whole group discussion. Since the worksheets and exercises for this toolkit utilize the American Association of Medical Colleges (AAMC) Entrustable Professional Activities (EPAs) for entering residency, this is a good point in the workshop to assess the audience familiarity with EPAs. For either the workshop audience or individual user that is less familiar with EPAs, a slide introducing this framework has been included to discuss this topic (Appendix A Slide 19). Facilitators may skip this slide or use as a brief review for audiences more comfortable with EPAs.

1. Workshop Break

The transition from the needs assessment exercise to the sample schedule exercise is a good time for a break for the participants. This break can also be used to rearrange the participant groups when they return from the break for the sample schedule exercise.

1. Sample Schedule
   1. Dividing the groups: Depending on the number and composition of your participants you may find one of several group divisions useful for this exercise. For our workshop, we divided based on course length (3 days or less, 5 days, 10 days, >10 days). Other options include: new bootcamp vs revision of existing bootcamp, clinical specialty (consider this if participants all from single institution), role / experience (administrative, preclinical faculty, clinical faculty, etc).
   2. Introduction of the exercise: Appendix A slide 22 gives an overview of the exercise and may be left on display during small group work for reference. Since there are multiple pages of handouts provided for this exercise, it is useful to orient the group to the materials. Participants should begin by reviewing the provided course content list, adding or eliminating content based on their individual needs assessments. Next, their content “wish list” is subdivided based on the prioritization worksheet. The example schedules provide an idea of the amount of content that can be included within a given time frame. Specifically, if groups are divided by course length, you can help them identify the sample schedules provided that they are of similar length. Groups use their own prioritized content and these examples to create a rough plan for their course schedule. For groups or individuals creating schedules of more than 1 week duration, it may be useful to print extra copies of the blank boot camp schedule worksheet.
   3. Group discussion: Appendix A slide 23 lists the prompts for group discussion. This section should be focused on how the decisions were made for content and priorities as well as challenges encountered, as these may be quite different between courses of different lengths. In our experiences, content included in the shortest courses was similarly prioritized in longer courses. Additional topics for discussion include when to offer the course, length of day, and breaks.
2. Module Development
   1. Introduction of the exercise: Based on the experience of your participant group, you may choose to briefly review the steps (Appendix A slide 24) and strategies (Appendix A slide 25) useful in module development. If you choose to go into further depth about how educational methods align with learning outcomes, Appendix F includes an adapted summary chart from which facilitators can select 3 or 4 examples such as task trainers for skills vs role play and standardized patients for behavioral objectives. It is not necessary to provide this chart to all participants, but this is another available resource. Talking points for some of the more common educational methods used in Boot Camps are provided in Appendix A slide 26. Make sure to instruct the groups to consider the number of learners, faculty / facility requirements, and time requirements in their planning.
   2. Dividing the groups: Appendix A slide 27 reviews instructions for this activity. In order to ensure a variety of module content topics and educational methods, we chose to assign each group 1-2 EPAs to cover in their module. Alternative structures based on workshop size/number of tables include allowing tables to choose any EPA, challenging them to cover 2 or more EPA topics in a single module, or having them adapt a published module from the provided list to their own institutional needs (requires personal computer and download capability by participants during the session).
   3. Group discussion: At the end of the module exercise, invite groups to share their module design and objectives for input and feedback from the group. Prompt questions including how they selected their educational method and whether this would be transferable to other institutions are included in Appendix A slide 28.
3. Barriers

In this section, facilitators should engage the participants in discussion of not only predicted barriers (Appendix A slide 29) but also possible solutions (Appendix A slide 30). Within our workshop, some of the most common barriers identified were money and time, corresponding to the authors’ own experiences. We have included a list of frequently encountered barriers as well as suggested solutions in the notes section of the slide.

1. Wrap up and Assessment

After asking for any participant questions and thanking them for their time, selected summary points (Appendix A slide 31) are provided for review. You may choose to give the initial post workshop survey to participants at the end of the session, or by mail/email after the session. For the author’s workshop, the survey responses were collected via SurveyMonkey. For facilitators with SurveyMonkey accounts, it may be possible to share the templates for both the post-workshop and follow up surveys. If you would like to request these, please contact Amanda Hartke at *Amanda.Hartke@PrismaHealth.org*.

1. Facilitating workshops for faculty with existing boot camps

Depending on the setting for the workshop, it is possible to have wide variation in both clinical and educational experience within the group. If this workshop is planned with faculty members that have an existing bootcamp, some adjustments can be made to the flow and exercises to accommodate for this.

Introduction, Audience Identification, and Review of Objectives

Depending on the number of participants, consider asking the audience for 1 thing that went very well in their bootcamp and 1 thing they would like to improve. Look for identified areas of improvement that correlate to the segments of the toolkit. For example, responses such as wanting better student engagement can relate to creating more engaging modules and institutional support and resources can be related to identifying stakeholder needs.

Objective Review

Using the responses from the participants, you can frame the objectives to match the identified areas for improvement. Comments included in Appendix A Slide 4 offer one framework for this.

Background literature

During the review of Appendix A slides 11-14, the facilitator can ask participants for a show of hands if their boot camp has a corresponding structure, duration, or assessment component. This information can help the facilitator in subsequent division into exercise groups without having each participant describe their personal bootcamp.

Workshop Exercises and Toolkit components

Since participants with boot camps may still have variable experience with curriculum development, we recommend following the same sequence for the majority of the exercises. For the Needs Assessment exercise, it may be useful to prompt any that are repeating a needs assessment to consider institutional/curricular/stakeholder changes in the interim. For the Sample Schedule exercise, participants with boot camps should be better able to divide into groups based on duration of the course or role/experience in course design. Although peer sharing is useful, you may consider leaving Appendix A slide 23 showing during the small group exercise in order to focus them on the exercise goals and avoid lengthy descriptions of individual bootcamp structures during this time. For the module development exercise, groups may choose to try and improve on an existing module from one of the participants, incorporate a second EPA into that existing module, or create an entirely new module based on their selected EPA.

Barriers and Solutions

For workshops with significant experience in prior boot camps, it may be useful to allow additional time for group discussion in this section. Peer sharing in this area can lead to rich discussions of both barriers and solutions.
